# The effects of ingested cellulose nanomaterials on DNA methylation in intestinal cells

**DOI:** 10.1186/s12989-026-00672-x

**Published:** 2026-03-12

**Authors:** Nádia Vital, Célia Ventura, Camila Fernandes, Luís Vieira, Ana Valente, Michel Kranendonk, Maria João Silva, Henriqueta Louro

**Affiliations:** 1 Department of Human Genetics, National Institute of Health Doutor Ricardo Jorge, Avenida Padre Cruz, 1649-016 Lisbon, Portugal; 2 NOVA Medical School, Universidade NOVA de Lisboa, Lisbon, Portugal; 3 Comprehensive Health Research Centre (CHRC), NOVA Medical School, Universidade NOVA de Lisboa, Lisbon, Portugal

**Keywords:** Nanocellulose, Nanomaterials, In vitro digestion, DNA methylation, Gene ontology, Pathway analysis

## Abstract

**Background:**

Cellulose nanomaterials (CNMs) are emerging materials under development for various applications in biomedicine and the food industry. Consequently, their potential human health effects warrant evaluation, particularly after oral exposure. Previous studies on two innovative CNMs, CNF-TEMPO and CMF-ENZ, indicated that genotoxicity could not be ruled out, as DNA damage was observed in Caco-2 cells. Whether these CNMs may affect gene expression regulation and induce downstream effects not assessed through genotoxicity testing, is still an open question. This work presents a new approach methodology coupling in vitro simulated digestion with epigenomics, to investigate changes in DNA methylation upon exposure of intestinal cells to these CNMs.

**Results:**

DNA methylation patterns differed upon cell exposure to undigested or digested samples, showing a trend towards hypermethylation for undigested and hypomethylation for digested samples. Using Reduced Representation Bisulfite Sequencing, significant differentially methylated regions (DMR) and genes were identified in undigested and digested CNMs-exposed cells. Pathway analysis revealed that CNMs targeted multiple signal transduction pathways. CMF-ENZ and CNF-TEMPO impacted glycosaminoglycan metabolism and glycosylation pathways, possibly involved in cell proliferation. CMF-ENZ affected a DNA repair pathway, while CNF-TEMPO impacted pathways associated with glucose metabolism, independently of the digestion process. Both CNMs seemed to affect pathways implicated in overall cytoskeletal and extracellular matrix dynamics. For digested CNMs, particularly digested CNF-TEMPO, the epigenetic modifications suggested a possible deleterious disturbance given the involvement of ERBB2/PIK3 signaling.

**Conclusions:**

This study shows that CNMs may have a biological effect on Caco-2 cells, affecting DNA methylation, and highlights the importance of epigenetic analysis in pointing out the way for unraveling the mechanisms underlying their toxicity.

**Supplementary Information:**

The online version contains supplementary material available at 10.1186/s12989-026-00672-x.

## Introduction

Nanocelluloses (or cellulose nanomaterials, CNMs), such as cellulose nanofibrils (CNFs), are versatile nanomaterials (NMs), widely studied for use in multiple fields, including the food and biomedical fields [1]. The toxicological effects of these NMs have been investigated using intestinal cells and showed, in general, a lack of mutagenic and carcinogenic properties [1–3]. For two CNFs obtained from *Eucalyptus globulus* kraft pulp, no consistent evidence of mutagenic and chromosomal damage has been shown, using OECD-recommended tests [3]. However, an induction of DNA damage was observed using the comet assay in Caco-2 and HT29-MTX-E12 intestinal cells, indicating that genotoxicity cannot be excluded [3]. In addition, there is evidence that cellulose nanocrystals (CNCs) but not CNFs, may exert inflammatory effects and immunotoxicity [2, 4, 5], although contradictory outcomes were seen for CNCs [5]. Indications of barrier function impairment after exposure to CNFs and CNCs in intestinal epithelia have also been reported, however, without affecting tissue viability [4, 5]. Given these inconclusive results, safety concerns regarding CNMs and their potential effect on human health remain and require further clarification.

DNA-damaging agents can affect DNA methylation patterns, a reversible epigenetic mechanism of gene regulation, which may have an important role in DNA damage and DNA damage response [6]. DNA methylation refers to the covalent transfer of a methyl group (CH3) to the C5 position of a cytosine nucleotide ring on a DNA strand (5-methylcytosine, 5-mC) [7]. Most frequently, these methylations occur at CpG sites (CpGs), that tend to cluster together in genomic regions known as CpG islands (CGIs) [7, 8]. Methylation of CpGs located in gene promoter regions is generally associated with transcriptional gene silencing, while gene body hypermethylation correlates with gene activation [9]. The well-balanced process between DNA methylation and demethylation is critical for the regulation of multiple biological processes related to development (e.g. during embryogenesis, cell differentiation, tissue development) and cellular homeostasis and may change due to aging, or disease (including cancer) processes and progression [8, 10, 11]. Epigenetic alterations play an essential role in carcinogenesis and, for instance, changes in DNA methylation have been implicated in the initiation and progression of colorectal cancer (CRC) [12].

Exposure to several NMs such as carbon-based NMs, silica, titanium dioxide, gold, and zinc oxide have been associated with DNA methylation changes in tumour suppressor genes, inflammation-related and DNA repair genes, among others, all of relevance for cancer development [13]. Moreover, changes in DNA methylation were observed in the genome of some cell types at noncytotoxic, low doses of NMs, suggesting that DNA methylation can be an early sensitive marker for detecting cellular responses upon exposure to NMs [14]. Exposure to NMs can also induce epigenetic effects through histone modifications and non-coding RNA alterations, including the epigenetic machinery itself [15]. Although molecular epigenetic effects of NMs do not directly translate into alterations of toxicological endpoints, including genotoxicity, they can elucidate their underlying mechanisms of action of substances, particularly related to low-dose and long-term effects [13, 16, 17]. Most epigenetic studies on NMs have been conducted in vitro, the majority focusing on lung-related cell models, and very few applying intestinal cells [13]. To the best of our knowledge, there are no studies that assessed genome-wide alterations in DNA methylation patterns caused by CNMs. A single study reported the immunomodulatory properties of CNFs in immune cells, related to their microRNA epigenetic profile [18].

In the present study, the epigenetic effects of two fibrillated CNMs, differing in their physicochemical properties, CMF-ENZ and CNF-TEMPO, undigested and after simulated digestion, were investigated in intestinal cells. This evaluation was performed using a new approach methodology combining the in vitro human digestion simulation established within INFOGEST2.0 with two approaches used to analyse DNA methylation patterns: global DNA methylation and genome-wide DNA methylation using the Reduced Representation Bisulfite Sequencing (RRBS). RRBS is a high-throughput method that uses restriction enzymes in the whole genome and DNA size selection before bisulfite conversion narrowing down the analysis to the CpGs-rich regions where most DNA methylation occurs, while only sequencing approximately 3–5% of the genome [19, 20]. This approach aids in identifying, in the genome of Caco-2 cells, the potential differentially methylated region (DMR), and the genes that undergo methylation changes after exposure to these CNMs, further exploring functional pathways that might be affected by the genomic methylation changes. Adding the in vitro simulated digestion before genotoxicity testing enables to consider the potential physicochemical modifications that may occur to the NMs along digestion, which might influence their interaction with cells.

## Materials and methods

### Cellulose nanomaterials preparation

CNF-TEMPO and CMF-ENZ samples, obtained from industrial bleached *Eucalyptus globulus* kraft pulp (BEKP), were kindly provided by the University of Coimbra and already characterized [3, 21, 22]. Details on CNF-TEMPO and CMF-ENZ production are described elsewhere [21, 23]. Briefly, to obtain the CMF-ENZ, cellulose fibres were firstly suspended in water and the pH adjusted to 5.0 with sodium citrate buffer. The suspension was incubated with 5% hemicellulase, 10% endocellulase, and 10% exocellulase at a dosage of 300 g/ton of fibres (enzymatic hydrolysis treatment), and heated for 2 h at 50 °C, under constant mechanical stirring. The hydrolysis reaction was halted by increasing the temperature to 80 °C for 15 min, then cooled to room temperature and washed with demineralized water. For CNF-TEMPO, fibres were subjected to a 2 h treatment with TEMPO-mediated oxidation. Firstly, fibres were mixed in an aqueous suspension with 0.016 g of radical 2,2,6,6-tetramethyl-1-piperidinyloxy (TEMPO) and 0.1 g of NaBr, per gram of fibres, at room temperature, and stirred for 15 min for good dispersion. After this, 5 mM of NaOCl per gram of fibres was slowly added to the dispersion. The oxidation was finished when a stable pH 10 was reached (by adding drops of NaOH 0.1 M). CNF-TEMPO was filtered and washed with distilled water until 20 µS/cm conductivity. CNF-TEMPO and CMF-ENZ were then mechanically homogenized in a high-pressure homogenizer (GEA Niro Soavi, model Panther NS3006 L, GEA Group Aktiengesellschaft, Düsseldorf, Germany) with 2 passages, the first at 500 bar and the second at 1000 bar [21].

Before each biological assay, both samples, presenting a gel consistency, were diluted to prepare a stock solution of 1.5 mg/mL in phosphate buffer saline (PBS), and dispersed with magnetic stirring for 30 min. Working dilutions were then prepared in a cell culture medium. Digested samples were treated according to “Simulated in vitro digestion” section and then diluted in a cell culture medium to reach final concentrations. In the biological assays, samples with and without digestion were used.

### Simulated in vitro digestion

The in vitro digestion experiment was performed with the harmonized static INFOGEST 2.0 digestion protocol to emulates the sequential human digestion process, using different conditions of digestion time, pH, temperature, ionic strength, bile salts content, and digestive enzymes to simulate the in vivo oral, gastric, and intestinal compartments [24, 25]. Three dispersions (simulated digestion fluids) with a specific electrolyte composition and concentration, were used to simulate the three phases of digestion: oral (simulated salivary fluid, SSF), gastric (simulated gastric fluid, SGF), and intestinal (simulated intestinal fluid, SIF) phase. The components of each simulated digestion fluid are described elsewhere [24, 26]. A minor modification of the reduction of bile salt concentration from 10 to 4 mM was introduced to the INFOGEST 2.0 digestion original protocol, as previously reported [3, 26]. At each digestive phase, the different fluids with the specific electrolyte composition (SSF, SGF and SIF), as well as specific enzymes, and other constituents were sequentially added to an initial volume of 1 mL of CNF-TEMPO or CMF-ENZ, as follows: (i) 1 mL of SSF (1X) with 75 U/mL α-amylase, 1.5 mM of CaCl_2_·2H_2_O and Milli-Q water, mixed in a mechanical shaker for 2 min at 37 °C (oral phase, pH 7); (ii) 2 mL of SGF (1×) with 2000 U/mL pepsin, 0.15 mM of CaCl_2_.·2H_2_O and Milli-Q water, mixed for 120 min at 37 °C (gastric phase, pH 3); (iii) 4 mL of SIF(1×) with 100 U/mL pancreatin, bovine bile (to achieve 4 µM final concentration of bile salt in the reaction vessel), 0.6 mM of CaCl_2_·2H_2_O and Milli-Q water, mixed for 120 min at 37 °C (intestinal phase, pH 7). At the end of the intestinal phase, the enzymatic activity was stopped with Pefabloc^®^ SC (final concentration 5 mM) [3, 26]. The final product of the digestion is thereafter denominated as “digestion product”, while CNMs submitted to digestion are designated as digested CNMs (DIG CNMs).

### Cell culture and exposure conditions

The in vitro cellular model based on human colorectal adenocarcinoma Caco-2 (European Collection of Authenticated Cell Cultures, ECACC, Salisbury, UK), was used to assess DNA methylation alterations. The cell line was cultured in Dulbecco’s Modified Eagle medium (DMEM) supplemented with 10% of fetal bovine serum (FBS), 1% penicillin/streptomycin, 1% fungizone and 2.5% HEPES buffer (all from Thermo Fisher Scientific, Waltham, MA, USA), at 37 °C and 5% CO_2_. They were routinely checked for mycoplasma using PCR. Before exposure, the cells were grown 24 h in 6-well plates at 1.5 × 10^5^ cells/mL. The cells were exposed to 3.1, 14.3, and 25 µg/mL of CNMs or digested CNMs, for 24 h at 37 °C and 5% CO_2_. Untreated cells were maintained as negative control of undigested CNMs exposure. The digestion product without CNMs was used as a negative control of the digestion, in the corresponding concentrations of digested CNMs (DIG Control, C1–C3). The percentage of digestion product present in the culture medium corresponding to the CNMs concentrations are the following: 3.1 µg/mL—1.7% (C1); 14.3 µg/mL—7.6% (C2) and 25 µg/mL—13.3% (C3). All exposure conditions were performed in biological triplicate cultures for each of the 16 treatment conditions.

### DNA extraction and quantification

Genomic DNA was extracted from exposed and non-exposed Caco-2 cells using the Wizard Genomic DNA Purification kit (Promega, Madison, WI, USA), according to the manufacturer’s protocol (3.D “Isolating genomic DNA from tissue culture and Animal tissue”), with the addition of a step of proteinase K digestion for 3 h (final concentration 100 µg/mL). DNA purity was evaluated using the Nanodrop (Thermo Fisher Scientific, Waltham, MA, USA), with acceptance criteria based on general guidelines for absorbance ratios (A260/280 ~ 1.8 for DNA purity; A260/230 > 2.0 for buffer/salt contamination). dsDNA was then quantified in Qubit 3.0 (Thermo Fisher Scientific, Waltham, MA, USA) using the dsDNA Broad Range (BR) Assay Kit (Invitrogen, Carlsbad, CA, USA). DNA quality was confirmed in a 0.8% agarose gel electrophoresis.

### Global DNA methylation analysis

DNA methylation was analysed by measuring the global levels of 5-methylcytosine (5-mC) using the methylated DNA Quantification Colorimetric Kit ab117128 (Abcam, Cambridge, UK), according to the manufacturer´s instructions. Briefly, 100 ng of DNA was added to a 96-well plate previously coated with a binding solution, and left to incubate at 37° for 90 min. Then, the plates were washed, incubated with a capture antibody for 30 min, rewashed, and incubated with a detection antibody for 30 min. Finally, an enhancer solution was added for 30 min followed by the developing solution. The absorbances (optical density, OD) were measured in a SpectraMax iD3 plate reader (Molecular Devices, San Jose, California, USA) at 450 nm. Concurrent positive (methylated polynucleotide containing 50% of 5-methylcytosine) and negative (unmethylated polynucleotide containing 50% of cytosine) controls provided by the manufacturer were included and used to determine the relative methylation status of undigested and digested CNMs DNA samples. From each exposure condition—3.1, 14.3, and 25 µg/mL of CNMs or digested CNMs, negative control and digestion controls—two out of three biological replicates were used for this assay in two different days, each with two technical replicates. Calculation of the percentage of 5-mC in total DNA was carried out using the following formula (Relative Quantification of 5-mC):

$$\begin{aligned} \mathrm{5-mC}{\%} & =\frac{(\text{OD sample}-\text{OD negative Control}) \div \mathrm{S}}{\left(\mathrm{O}\mathrm{D}\text{ p}\mathrm{o}\mathrm{s}\mathrm{i}\mathrm{t}\mathrm{i}\mathrm{v}\mathrm{e}\text{ C}\mathrm{o}\mathrm{n}\mathrm{t}\mathrm{r}\mathrm{o}\mathrm{l}-\mathrm{O}\mathrm{D}\text{ N}\mathrm{e}\mathrm{g}\mathrm{a}\mathrm{t}\mathrm{i}\mathrm{v}\mathrm{e}\text{ C}\mathrm{o}\mathrm{n}\mathrm{t}\mathrm{r}\mathrm{o}\mathrm{l}\right)\times 2\div \mathrm{P}} \\ & \quad \times100\mathrm{\%},\end{aligned}$$

where S is the amount of input sample DNA. P is the amount of input positive control.

### Reduced representation bisulfite sequencing

RRBS libraries were constructed with the Premium RRBS kit V2 *Reduced Representation Bisulfite Sequencing for Illumina Platforms* (Cat. No. C02030036, v2, Agosto 2022. Diagenode, Seraing, BE), according to the manufacturer’s user guide [27]. Briefly, 100 ng of DNA obtained from Caco-2 cells exposed, for 24 h, to 14.3 µg/mL (three replicates/sample) of CNMs were digested with a restriction endonuclease *MspI* (C/CGG), to obtain DNA fragments containing CpGs. Digestion was followed by end-repairing reaction of the resulting fragments, A-tailing, and Illumina adapter ligation. Then, libraries were size selected using AMPure XP magnetic beads (Beckman Coulter, Pasadena, CA, USA #A63881) and quantified by qPCR (7500 Real-Time PCR System, Applied Biosystems), using the SyBRGreen PCR master mix (Applied Biosystems, Foster City, CA, USA). Libraries with similar qPCR Cycle thresholds (Ct) values were multiplexed in pools of three. Pooled libraries were treated with sodium bisulfite to deaminate unmethylated cytosine residues, converting them to uracil residues [28, 29]. The bisulfite-converted DNA library pools were quantified by qPCR to determine the optimal number of amplification cycles for each pool and bisulfite-converted DNA was enriched by PCR amplification. The libraries’ profile and medium size were assessed in the Fragment Analyser system (Agilent, Santa Clara, CA, USA) using the DNF-474 High Sensitivity NGS Fragment Analysis Kit (1 bp−6000 bp), and the PROSize software (Agilent). Libraries were quantified with Qubit (Thermo Fisher Scientific, Waltham, MA, USA), using the dsDNA High Sensitivity Assay Kit (Invitrogen). RRBS Library pools were pooled equimolarly and sequenced using 50 bp paired-end reads on a NextSeq 2000 instrument (Illumina, San Diego, CA, USA). The fastq files were generated automatically on-instrument following the end of the sequencing run.

### Bioinformatics analysis of RRBS data

**Fig. 1:**
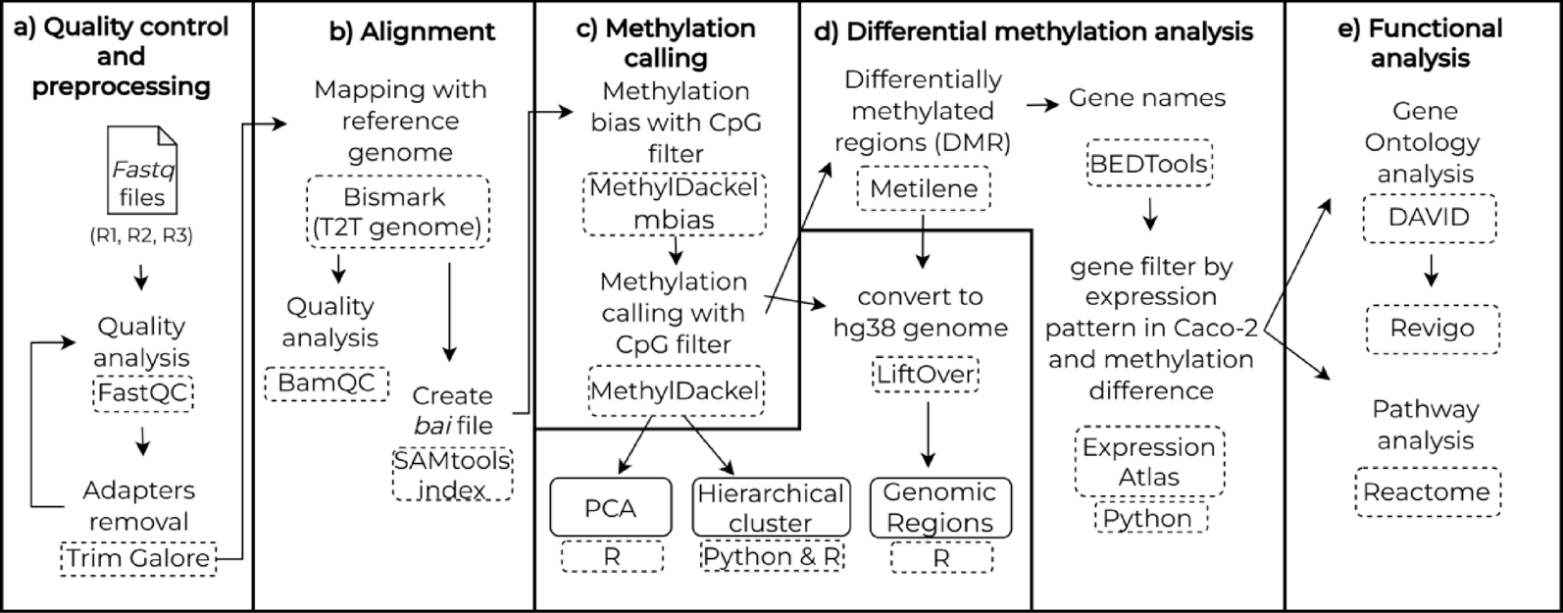
Overview of the bioinformatics analysis performed with the RRBS samples. Rounded squares with dot lines correspond to the tools used at each step of the analysis. **a**) Quality control and preprocessing, **b**) alignment, **c**) methylation calling, **d**) differential methylated analysis, **e**) functional analysis

The RRBS data analysis was based on the workflow and pipeline developed by Valente et al. [30, 31], with minor modifications. The data processing steps are represented in Fig. 1.

#### Quality control and preprocessing of reads

The data analysis started with the quality control of the Illumina sequencing reads using FastQC version 0.12.1 software [32]. Adapter sequences were removed using Trim Galore version 0.6.7 with the options –rrbs –non_directional [33]. FastQC was run on the resulting output files once trimming was completed to confirm the removal of the adapter sequences (Fig. 1a).

#### Alignment to the reference genome

Bisulfite-treated sequencing reads were mapped (Fig. 1b) to the human reference genome CHM13 2.0 (T2T genome) using the Bismark Mapper version 0.22.1, with default parameters, and applying the options –score-min ‘L,0,-0.6’ and –pbat option [34, 35]. Mapping quality control was assessed using QualiMap BamQC version 2.3 [36, 37]. After mapping, bam files were sorted and indexed (bai files) according to genomic coordinates using SAMtools program (https://www.htslib.org/) version 1.20 [38].

#### Methylation calling

Following alignment, the methylated sites were identified and quantified by estimating proportion-based identification considering the methylation levels at each site through statistical analysis using MethylDackel software version 5.2 [39]. Firstly, a methylation bias plot (MethylDackel mbias) was created, using the bam file sort and bed file of CpGs of regions for inclusion, for each of the sample replicates. The bed file of CpGs containing CGIs genomic coordinates (CpGislandext.bb) was retrieved from the UCSC Genome Browser Downloads (https://hgdownload.soe.ucsc.edu/gbdb/hs1/bbi/). Methylation calling per-base metrics (Fig. 1c) was then extracted with default parameters, using the inclusion bounds suggested in MethylDackel mbias, and filtered using the same bed file mentioned above.

#### Differential methylation analysis

After methylation calling, only the CpGs with at least 3x coverage were retained for the identification of DMR. DMR were identified by comparing non-exposed and exposed samples using the Metilene version 0.2.6 (Fig. 1d) with default parameters [40].

To identify the genes overlapping or nearest significant DMR, the coordinates of the Metilene output file were compared with the T2T-CHM13v2.0 genome annotation (https://www.ncbi.nlm.nih.gov/datasets/gene/GCF_009914755.1/) containing all gene coordinates, using the BEDTools closest program version 2.31.0 [41]. The resulting BedGraph file with the closest genes to the DMR for each sample was then compared to a file containing the number of transcripts per kilobase million (TPM) of human genes expressed in Caco-2 cells, retrieved from the European Bioinformatics Institute: https://www.ebi.ac.uk/gxa/experiments/E-MTAB-2770/Results), using a Python script [31]. A filter was applied to include in the downstream analysis, all the genes shown to be hypomethylated, and only the hypermethylated genes expressed above 5 TPM. A gene list (txt file) was produced for each CNMs with or without digestion.

#### Functional analyses

Pathway and Gene Ontology (GO) analysis of the gene lists was performed to predict the impact of methylation changes in pathways and biological processes (Fig. 1e). The retrieved genes were classified according to GO terms, using the DAVID (Database for Annotation, Visualization and Integrated Discovery) tool [42, 43], with the options “GOTERM_MF_ALL”, “GOTERM_CC_AL” and “GOTERM_BP_ALL”, concerning the molecular function of the gene products, the cellular component where they are active, and the biological process made up of the activities of multiple gene products, respectively, provided by the Gene Ontology Consortium (http://www.geneontology.org/). Then, redundant GO terms were reduced and summarized with REVIGO (REduce and VIsualize Gene Ontology) [44], using the Homo sapiens model (9606), and a semantic similarity cutoff of C = 0.70. The same gene lists obtained for each CNMs were uploaded to the Reactome web server (https://reactome.org/) for the pathway analysis [45]. This software performs an enrichment analysis of the predicted target genes. As this is an exploratory study, in both analyses, top-ranking pathways were retrieved based on raw entities p value lower than 0.05 without correction for multiple comparisons, since after increasing stringency, many pathways did not meet the False Discovery Rate (FDR) threshold of 0.05.

### Statistical analysis and graphical representations

Statistical analyses and graphs were performed using GraphPad Prism (GraphPad version 5, San Diego, CA, USA) for the global methylation analysis, with results presented as the mean ± standard deviation, unless specified otherwise. To determine the type of data distribution (parametric or nonparametric), the Shapiro–Wilk normality test and the test for the equality of variances were applied. The differences in means between samples and the respective negative control were then analyzed with one-way analysis of variance (ANOVA), followed by Dunnett’s Multiple Comparison Test *post-hoc* test. For comparing digested vs. undigested samples, the unpaired Student’s *t*-test was used.

Concerning the statistical analysis of genome-wide methylation data, a one-way ANOVA was used to compare the mean methylation metrics obtained with MethylDackel. The result was considered significant if the *p* value was equal to or lower than 0.05.

To visualize and identify patterns among all replicates, correlation analysis and unsupervised principal component analysis (PCA) were performed in R, with the output bedGraph files from the MethylDackel, after methylation calling with the CpG filter and the 3× coverage filter. As input to the PCA plot, the top 10% of CpGs with the highest variance among the mean of the methylation frequency (MF) of each CpGs were selected for each CNMs replicate [46–48]. The prcomp and ggplot2 [49] libraries were used to perform and visualize the PCA components. The similarity of sample replicates in common sites was checked by calculating pairwise Pearson’s correlation using the package Corrgram [50].

Hierarchical clustering analysis and genomic region annotations were obtained from bedGraph files from the MethylDackel, after methylation calling and CpGs filter, using two scripts in Python and R [31]. For the hierarchical clustering analysis, the Python script function received a unified DataFrame with the identified CpGs and respective MF filtering the CpGs having at least a 50% difference between the mean MF of the non-exposed replicates and the mean MF any of the replicates of CMF-ENZ or CNF-TEMPO, separately for undigested and digested samples. The output csv file was then imported into R to plot the heatmap using the gplots library [51]. The CpGs and the DMR were used for genomic region annotations using the R (v. 4.2.2) package “annotatr” version 1.24.0 [52]. The coordinates from the CpGs and DMR in the T2T-CHM13v2.0 genome were first converted to the human genome GRCh38, using the alignment LiftOver tool (https://genome.ucsc.edu/cgi-bin/hgLiftOver, retrieved in 2024-07-23), with the default parameters. This step was performed because the annotations for the T2T-CHM13v2.0 genome are still not available in the in the annotatr. For the CpGs, a cutoff of 50% between the mean MF of controls and each CNMs, was applied in the Dataframe resulting in a different number of CpGs for each CNMs. All the hypo- and hypermethylated DMR were used as input for the analysis of the genomic region annotations using the BedGraph files from Metilene.

## Results

### Effects of CNMs exposure on global DNA methylation

**Fig. 2:**
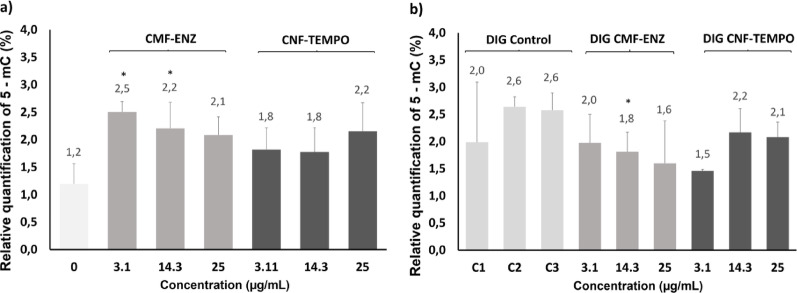
Percentage of 5-methylcytosine (5-mC) content in Caco-2 cells following 24 h exposure to (**a**) undigested and (**b**) digested CMF-ENZ and CNF-TEMPO. Baseline control values are represented by 0—negative control and C1–C3—DIG 0 controls (digestion vehicle controls matching the respective exposure volume fractions: 1.7%, 7.6%, and 13.3%). Results are presented as mean ± SD (*n* = 3). *Significantly different from the respective negative controls (*p <* 0.05, one-way ANOVA and post-hoc tests; Student’s t-test was utilized for digested vs. undigested comparisons

Quantification of 5-methylcytosine revealed significant differences after exposure to undigested CMF-ENZ (*p* = 0.0143; one way-ANOVA) when compared to the control, with increased levels after exposure to the two lowest concentrations of undigested CMF-ENZ (Fig. 2). For the digested counterpart, DIG CMF-ENZ, a slight decrease was observed compared to the respective control, especially at the two highest concentrations tested, but the difference was only significant for the 14.3 µg/mL concentration (*p* = 0.0127; Student’s *t* test). CNF-TEMPO, either digested or not, had no impact on global DNA methylation.

### RRBS

#### Correlation and PCA analysis

The average quality score per read was Q33 or higher and the mode of sequence quality across all sequences was Q33, for every sample, with an average percentage of CG content of 30% and adapter content below 5%, for all samples (Supplementary file Fig. S1 and Table S1). Following read mapping to the reference genome after CGIs filter (Supplementary file Table S2), the methylation calling resulted in approximately 3–4% (1201315–13089 CpG Sites) methylation sites compared to the total number of CpGs (32.28 Million) present in the human T2T-CHM13v2.0 sequence [53]. Differences were observed between the mean genome methylation levels of the CGIs in DNA from CNMs-treated cells compared with those from non-exposed cells (one-way ANOVA, *p* = 0.0005). For digested samples, no differences were observed between the genome methylation levels of CNMs-exposed cells and those from DIG control samples (one-way ANOVA, *p* = 0.4252). Considering only the common CpGs amongst all undigested (989353 CpGs) sample replicates, a strong positive correlation in the MF was found between each CNMs (CMF-ENZ: 0.94 ≤ *R* ≥ 0.97; CNF-TEMPO: 0.95 ≤ *R* ≥ 0.97) and the negative control, and in between replicates (Supplementary file Fig. S2). Likewise, amongst the common CpGs of all digested (1015823 CpGs) sample replicates, exposed cells showed a strong positive correlation in the MF among replicates and when compared to DIG control samples (CMF-ENZ: 0.97 ≤ *R* ≥ 0.98; CNF-TEMPO: 0.95 ≤ *R* ≥ 0.97).

For undigested samples, the PCA analysis of 10% of CpGs with the highest variance among undigested samples showed no variation among CNF-TEMPO replicates, negative control replicates or between the two (Supplementary file Fig. S3 and Table S3). CMF-ENZ replicates showed some variation among replicates. Digested samples also showed similarities among replicates and between CNMs replicates and control replicates, despite a small variance observed in one of the replicates for each DIG CNMs.

#### Hierarchical clustering

**Fig. 3:**
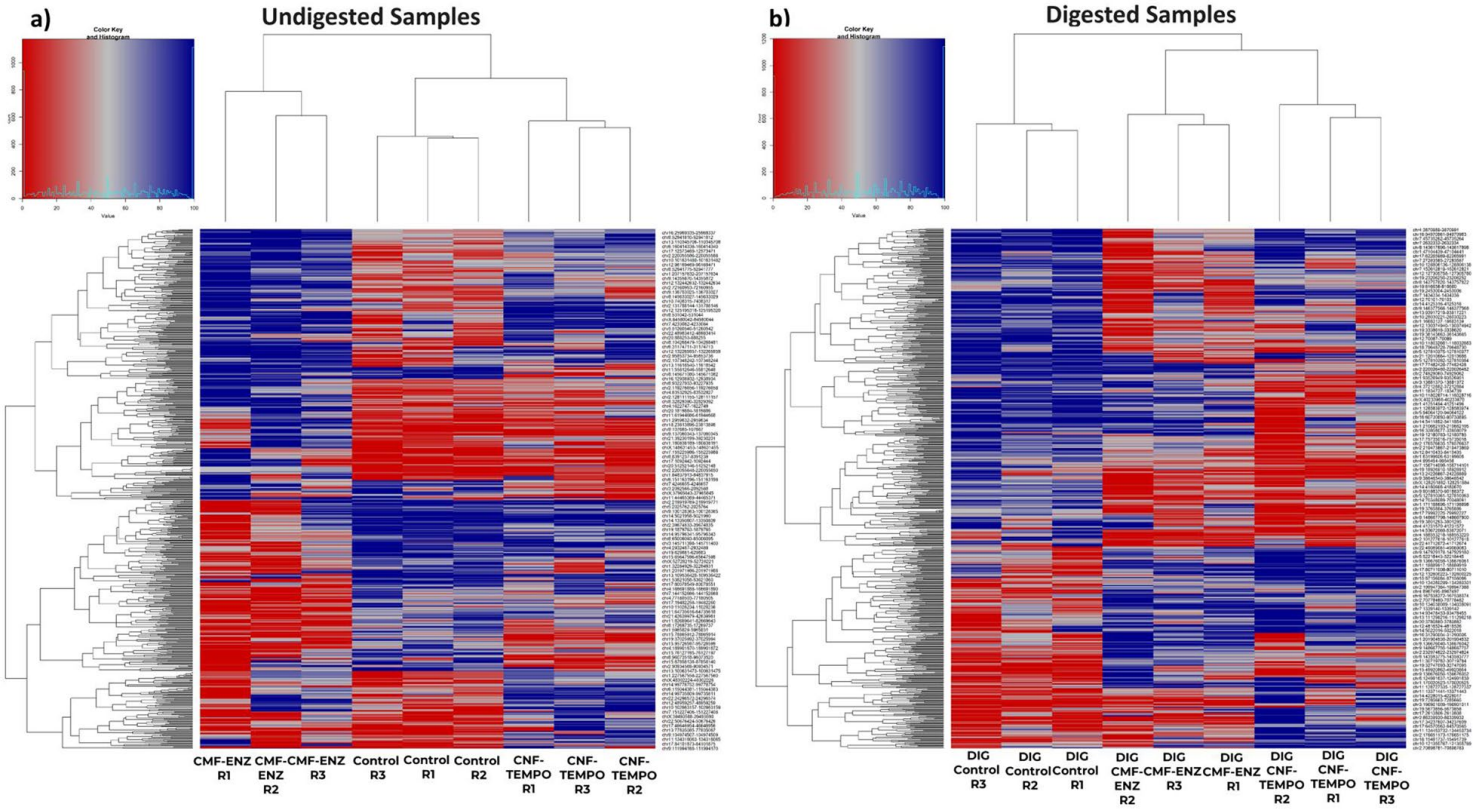
Heatmaps showing the results of hierarchical clustering analysis between CpGs from Caco-2 cells exposed and non-exposed to CNMs. **a** Undigested samples, **b** digested samples. The MF ranges from red (hypomethylation) to blue (hypermethylation). Samples are represented as columns and CpGs are represented as lines

Narrowing the analysis to consider the difference of 50% in the MF between the controls and at least one of the CNMs samples, hierarchical clustering analysis revealed unique methylation patterns among the undigested or digested CNMs, similar among replicates (Fig. 3). In undigested samples, in the heatmap containing 684 CpGs, CMF-ENZ replicates presented a distinct pattern compared to the negative control and CNF-TEMPO replicates (Fig. 3a). Regarding digested samples, the heatmap containing 717 CpGs showed that DIG CMF-ENZ and DIG CNF-TEMPO samples presented a distinct pattern to DIG control (Fig. 3b).

#### CpG sites and differentially methylated regions genomic annotation

Exposure of cells to undigested or digested samples led to a similar number of CpGs having MF differences higher than 50% when compared with the respective negative controls – 498 methylated CpGs for CMF-ENZ, 224 for CNF-TEMPO, 349 for DIG CMF ENZ, and 415 for DIG CNF-TEMPO (Table 1 and Supplementary file Fig. S4). Cells exposed to undigested CMF-ENZ showed higher MF differences in the genome compared to the control than those exposed to the digested counterpart. Undigested CNF-TEMPO produced fewer CpGs with a MF difference higher than 50% compared to the control. After exposure to DIG CNF-TEMPO, the MF difference increased. Genomic annotation analysis using these CpGs showed a similar distribution of the CpGs along the genome, for undigested and digested CNMs, suggesting that differences in methylation upon exposure to either one of the CNMs occurred independently of the genomic context.

**Fig. 4:**
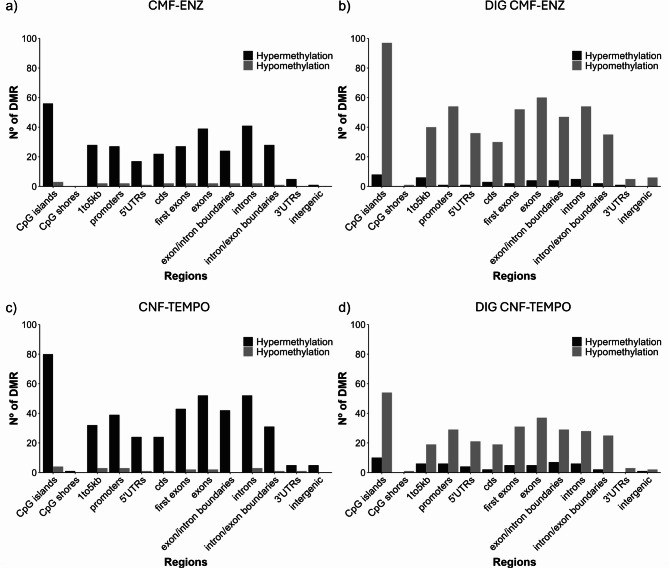
Distribution of identified differentially methylated regions (DMR) according to genomic annotations in Caco-2 cells after a 24 h exposure to the undigested (**a**, **c**) and digested (**b**, **d**) CMF-ENZ or CNF-TEMPO

At the regional level, comparison of each treatment against its specific control identified distinct profiles of DMR (Table 1; Supplementary Files: Tables S4–S7). Both undigested and digested CNMs had similar numbers of DMR. The undigested CNF-TEMPO generated a higher number of DMR than CMF-ENZ, whereas the inverse was found for digested CNMs (Table 1 and Supplementary file Tables S4, S5, S6 and S7). It can be noted that the digested samples produced a very different methylation profile than the undigested samples, with much more hypomethylated regions. However, the DMR were similarly distributed across the genomic annotations for the two CNMs and for both undigested and digested samples, with no preferential or disproportionate enrichment in gene promoter sequences (Fig. 4). No evident enrichment of gene promoter regions could be detected compared with other gene regions.

**Table 1:** Number of CpGs with a difference ≥ 50% in the MF and differentially methylated genomic regions (DMR) induced by exposure to the two types of undigested or undigested CNMs

	Undigested		Digested	
	CMF-ENZ	CNF-TEMPO	DIG CMF-ENZ	DIG CNF-TEMPO
Number of CpGs	498	224	349	415
Hypomethylated	3	4	97	54
Hypermethylated	56	80	8	10
Total	59	84	105	64

#### Genes in the differentially methylated regions

**Fig. 5:**
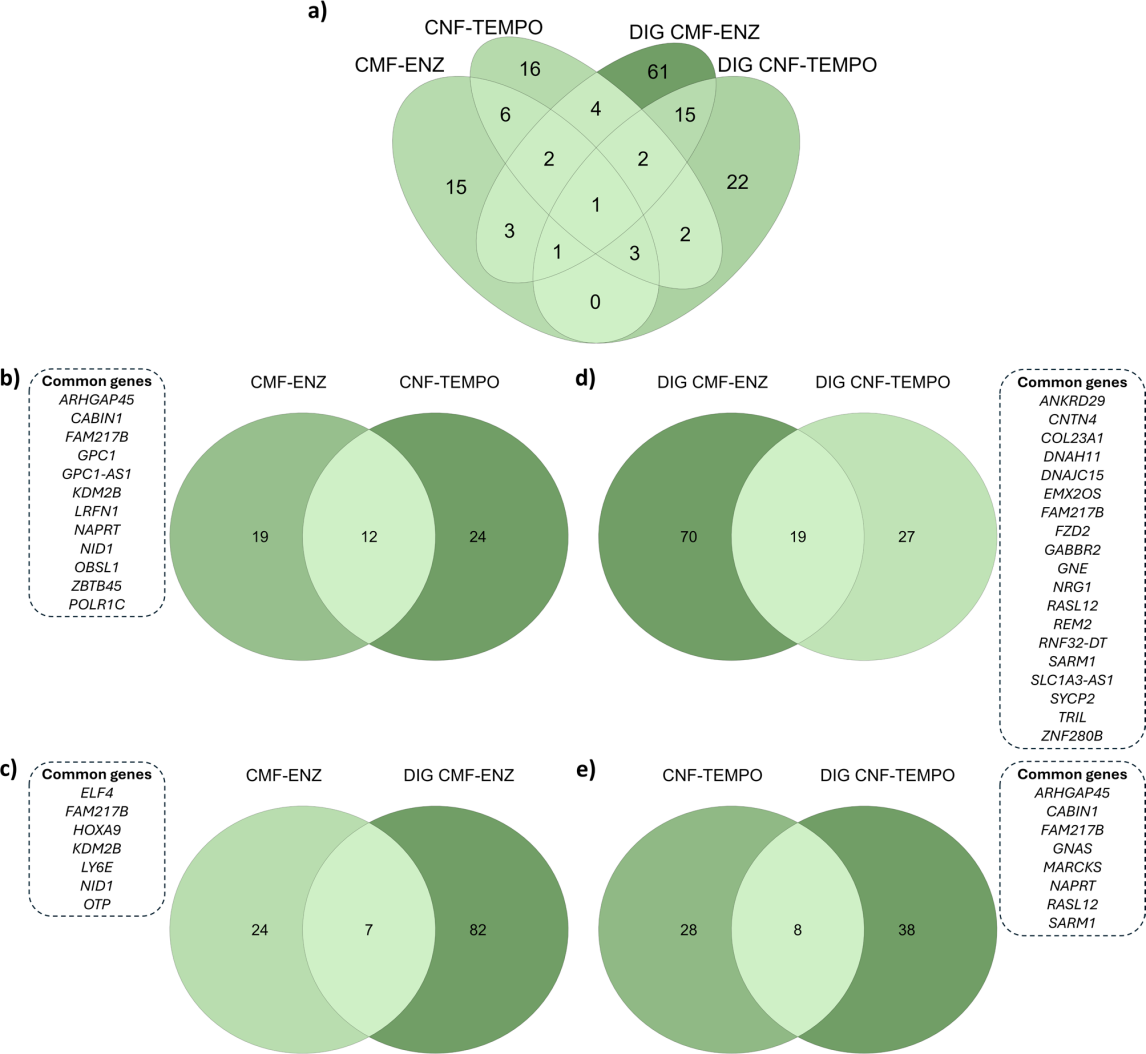
Venn diagram showing the genes identified in the DMR after exposure to CMF-ENZ, CNF-TEMPO, DIG CMF-ENZ, and DIG CNF-TEMPO

A total of 246 genes were identified overlapping or adjacent to DMRs across all conditions, with 70 genes shared between multiple treatment groups (CMF-ENZ: 68; CNF-TEMPO: 88; DIG CMF-ENZ: 116; DIG CNF-TEMPO: 65). These 246 genes were filtered for genes expressed in Caco-2 cells, which resulted in 153 genes, with 53 shared genes (CMF-ENZ: 31; CNF-TEMPO: 36; DIG CMF-ENZ: 89; and DIG CNF-TEMPO: 46), as represented in Fig. 5. The genes with the absolute MF difference between CNMs and the respective control are presented in Supplementary file Tables S4–8.

### Functional analysis

**Fig. 6:**
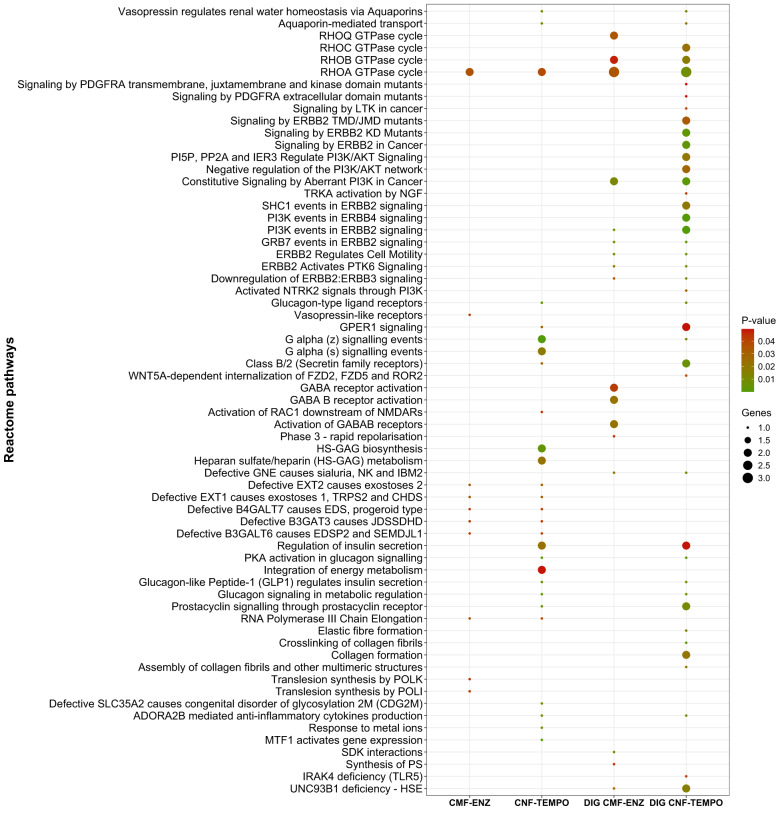
Pathway-enrichment analysis for the target genes significantly differentially methylated after exposure to undigested and digested CNMs. Pathways were selected based on *p* < 0.05, ranging from green (lower) to red (higher)

The analysis of Reactome pathways of the genes associated with the DMR resulted in 10, 27, 17, and 41 pathways having entities p values < 0.05, in CMF-ENZ, CNF-TEMPO, DIG-CMF ENZ, and DIG CNF-TEMPO exposed cells. The Supplementary file Table S9 details the functional pathways for all CNMs samples and the corresponding genes. Table 2 presents the identified genes in DMR that participate in the identified Reactome pathways enriched after exposure to undigested and digested CNMs. A total of 65 pathways were identified for both undigested and digested samples, as presented in Fig. 6.

**Fig. 7:**
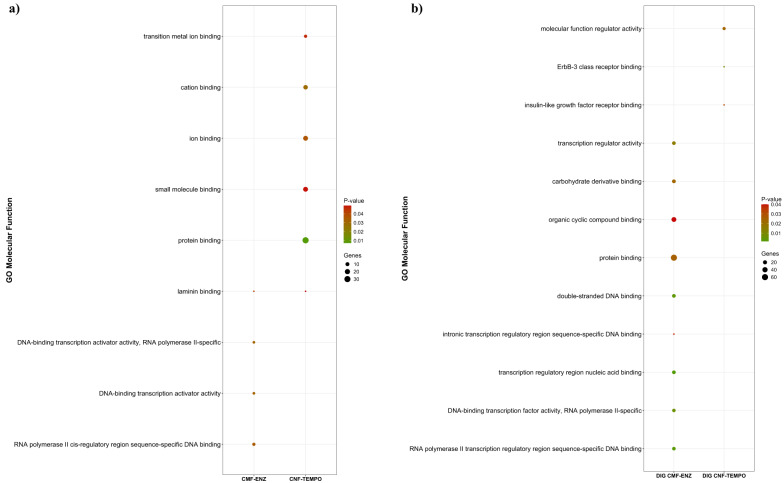
GO molecular function enriched with the genes significantly differentially methylated after exposure to (**a**) undigested and (**b**) digested CMF-ENZ and CNF-TEMPO. GO terms were selected based on *p* ≤ 0.05, ranging from green (lower) to red (higher)

Gene ontology analysis resulted in 25, 12, 74, and 9 enriched biological process (BP) clusters affected by the treatments with CMF-ENZ, CNF-TEMPO, DIG CMF-ENZ, and DIG CNF-TEMPO, respectively (Supplementary file Tables S10 and S11). For the cellular components (CC) undigested CMF-ENZ, CNF-TEMPO and DIG CMF-ENZ exposure enriched 10, 3 and 3 terms, respectively, while no CC terms were enriched after DIG CNF-TEMPO exposure (Supplementary file Tables S12 and 13). Enriched GO terms related to the molecular function (MF) are presented in Fig. 7 and detailed in Supplementary file Tables S14 and S15.

**Table 2:** Annotated genes in the DMR genes identified in the Reactome for each condition tested

Undigested				Digested			
CMF-ENZ		CNF-TEMPO		DIG CMF-ENZ		DIG CNF-TEMPO	
Gene	MF difference	Gene	MF difference	Gene	MF difference	Gene	MF difference
*FAAP20*	43.7	***MARCKS***	39.8	*ADCY1*	− 36.7	*LOXL2*	− 35.3
*OXTR*	33.9	*TIMM17B*	34.5	***TRIL***	− 34.7	***TRIL***	− 34.2
*PLEKHG5*	20.6	*HS6ST1*	31.3	*STOM*	− 34.5	***MARCKS***	− 31.5
***ARHGAP45***	15.4	*RGS20*	− 29.9	*FGF8*	− 30.7	***ARHGAP45***	27.0
***GPC1***	− 12.2	*CCDC106*	21.8	*GABBR2*	− 30.1	***GNAS***	− 26.2
***POLR1C***	11.8	***ARHGEF1***	18.9	***ARHGEF1***	− 28.7	*PIK3R1*	− 25.0
		***POLR1C***	17.3	*OBSCN*	− 28.0	*FZD2*	− 24.0
		***GNAS***	16.6	*KCNE3*	− 21.8	***NRG1***	− 24.2
		*PDE8B*	− 15.9	***GNE***	− 20.4	*PKN3*	− 23.0
		***ARHGAP45***	13.3	*SDK1*	− 18.8	*ZNF597*	− 20.6
		***GPC1***	− 12.1	***NRG1***	− 17.8	***GNE***	− 19.5
		*CSRP1*	6.2			*COL23A1*	− 18.6

In bold, genes shared between 2 or 3 CNMs

The signalling transduction by RHOA GTPases pathway was an enriched pathway common to all samples. GTPases are small signalling G proteins from the RHO family that switch “on” or “off” signal transduction pathways in response to chemical or mechanical stimuli. The *PLEKHG5*, *ARHGAP45*, and *ARHGEF1* genes, methylated after exposure to undigested or digested CNMs, are involved in the regulation of the RHOA GTPases activation/deactivation. DIG CMF-ENZ and DIG CNF-TEMPO enriched additional pathways related to other GTPases—the RHOB, RHOQ GTPase cycle, and RHOC GTPase, the latter only by DIG CNF-TEMPO.

The pathways that were common to CMF-ENZ and CNF-TEMPO were mostly related to the hypomethylation of the *GPC1* gene, which encodes the heparan sulfate proteoglycan (HSPGs) Glypican-1 (GPC-1). GPC-1 is composed by Heparan sulfate (HS) glycosaminoglycans (GAG) attached to a core protein. These pathways are related to the metabolism of carbohydrates, particularly the glycosaminoglycan metabolism in a disease context (5 enriched pathways associated with the *GPC1* gene). CNF-TEMPO enriched two additional associated pathways, particularly the Heparan sulfate/heparin (HS-GAG) metabolism (genes *GPC1* and *HS6ST1*), related to post-translational modifications to the sulfation of the 6-O positions by the 6-O sulfotransferases *(HS6ST1)*. One molecular function term was commonly enriched in Caco-2 cells after undigested CMF-ENZ and CMF-TEMPO exposure, namely, laminin-binding (GO:0043236), a major glycoprotein constituent of the cell basement membrane, to which the *GPC1* and *NID1* genes were assigned. In addition, cells exposure to both CNMs altered the pathway “RNA Polymerase III Chain Elongation” during gene transcription (with the participation of the *POLRIC 1* gene).

Concerning the undigested CMF-ENZ, few pathways were found to be enriched. Two pathways were associated with DNA Damage bypass (Translesion synthesis by POLK and Translesion synthesis by POLI) in which the Fanconi anemia core complex-associated protein 20 (FAAP20) *encoded by the* gene *FAAP20* participates. One pathway was related to RNA polymerase III chain elongation during gene transcription (with the participation of the *POLR1C* gene). Several molecular function and BP enriched terms were associated with binding to transcription regulatory regions (GO:0000977, GO:0000981, GO:0001067, GO:0001161, GO:0003690, GO:0140110), involving the methylated genes *POLR1C*, *HOXA9*,* KDM2B*,* ELF4*,* ZBTB45*,* ZNF280B and SOX12*, among others. These encode transcription factors or components of the transcription regulation machinery. CMF-ENZ exposure also enriched the “Vasopressin-like receptors” pathway (gene *OXTR)*, belonging to the G-protein-coupled receptors (GPCRs) family leading to the activation of several signalling pathways, such as the activation of the RhoA/Rho-associated pathways. In the GO analysis the *PLEKHG5* and *OXTR* genes were assigned to BP terms associated with the positive regulation of cellular processes (GO:0048522; GO:0048518). Other enriched BP were mostly related to tissue/cell development and differentiation.

CNF-TEMPO exposure enriched 10 pathways related to GPCRs downstream signalling and ligand binding, with the participation of the hypermethylated *GNAS*, *MARCKS*,* RGS20* genes, and the hypomethylated *PDE8B* gene. The *GNAS* gene (Nucleotide binding protein, alpha-stimulating activity polypeptide) encodes the G-protein (s) stimulatory alpha subunit (Gαs) of the G proteins complex. Six of these pathways are related to energy metabolism—glucagon and the regulation of insulin secretion (*MARCKS* and *GNAS* genes). These pathways include the class B/2 (Secretin family receptors), signalling by the Gαs (*GNAS* and *PDE8B* gene*)* and by G-protein (z) alpha subunit (Gαz) (*GNAS* and *RGS20* gene*).* CNF-TEMPO also enriched the “ADORA2B mediated anti-inflammatory cytokines production” (*GNAS* gene), two pathways related to the transport of small molecules (aquaporin-mediated transport), and two related to the cellular response to metal ions (*CSRP1* gene). The enriched molecular function terms after CNF-TEMPO exposure were related to molecular binding (protein, GO:0005515; cation, GO:0043169; ion, GO:0043167; small molecule, transition metal ion, GO:0046914). Fewer BP terms were retrieved, related to the regulation of signal transduction (GO:0010646, GO:0023051), and cell communication (GO:0010646). The hypermethylated genes *GNAS*,* ARHGAP45*, *ARHGEF1*,* NID1*, and the hypomethylated genes *RGS20*,* PDE8B*,* GPC1* were assigned to these terms.

After digestion, DIG CMF-ENZ and CNF-TEMPO shared several pathways related to the signal transduction by receptor Tyrosine Kinases (TK) ERBB family, involving Erbb2:Erbb3 or Erbb2:Erbb4 heterodimerization. The proteins encoded by *NRG1* and *PIK3R1 genes*, hypomethylated after exposure to DIG-CMF-ENZ and CNF-TEMPO participate in these pathways. Through GO analysis, after exposure to DIG CNF-TEMPO, these genes were assigned to the molecular function term “ErbB-3 class receptor binding” (GO:0043125).

After exposure to DIG CMF-ENZ, the *NRG1* genes were allocated to BP or molecular function related to positive regulation of cell population proliferation (GO:0008284; GO:0008283), cell differentiation (GO:0030154), cell communication (GO:0007154), and cell surface receptor signalling pathway (GO:0007166). DIG CMF-ENZ enriched molecular function terms related to ligand binding to the cell surface (including carbohydrate derivative binding). As seen after CMF-ENZ exposure, a few enriched molecular function terms were related to transcription - binding to transcription regulatory regions (GO:0000977, GO:0000981, GO:0001067, GO:0001161, GO:0140110, GO:0003690), to which the *HOXA9*,* KDM2B*,* ELF4*,* ZBTB45*,* SOX12*,* ZNF280B*,* ZNF664*,* ZNF662*, among other genes, were assigned. The enriched BP terms were broader and less informative, many of which related to tissue/cell development and differentiation.

**Fig. 8:**
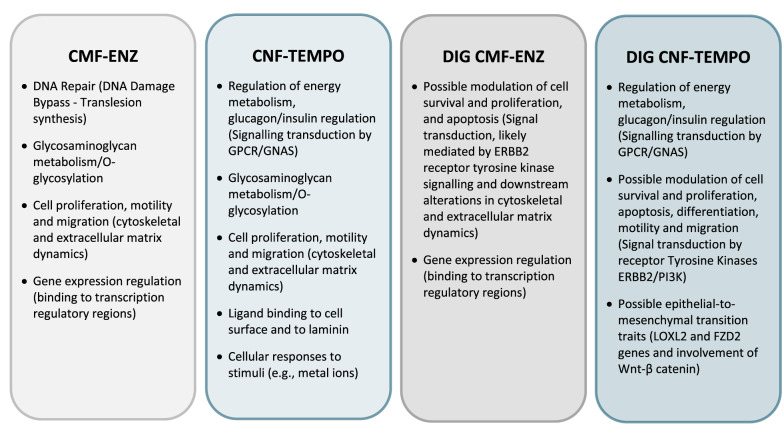
Summary of the main findings from Reactome and GO functional analysis

Exposure to DIG CNF-TEMPO enriched pathways related to the extracellular matrix (ECM) organization including collagen and elastic fiber formation (genes *COL23A1* and *LOXL2*), and Wnt-mediated signal transduction (WNT5A-dependent internalization of FZD2, FZD5 and ROR2). Moreover, DIG CNF-TEMPO and CNF-TEMPO shared the above-mentioned pathways related to the *GNA*S gene, i.e., energy metabolism of glucagon and regulation of insulin secretion, class B/2 Secretin family receptors, signalling by G-protein Gαz, and ADORA2B mediated anti-inflammatory cytokines production. DIG-CNF-TEMPO enriched the molecular function insulin-like growth factor receptor binding (GO:0005159, assigned to *GNAS*,* PIK3R1* genes). Figure 8 summarizes the main findings for each CNMs, listed in no order.

## Discussion

Epigenetic effects of two CNMs with different physicochemical properties, CMF-ENZ and CNF-TEMPO, were studied by investigating alterations in DNA methylation in Caco-2 cells. Global DNA methylation was generally increased upon cells exposure to CNMs, particularly for CMF-ENZ. Conversely, a trend toward hypomethylation was observed upon exposure to digested CNMs, especially to 14.3 µg/mL of DIG CMF-ENZ. With RRBS, a similar number of methylated CpGs and DMR was identified in undigested and digested CNMs-treated cells. In line with the previous results, treatment with undigested CNMs resulted in a clear tendency to DNA hypermethylation of Caco-2 cells while digested CNMs samples produced more hypomethylated regions. For both undigested CNMs, the DMR distribution was similar across the genome, suggesting that the genomic context did not influence the effect on methylation.

Pathway and Gene Ontology analyses of the differentially methylated genes encompassed by the DMR showed that both undigested CNMs induced alterations in pathways related to GAG metabolism, particularly the biosynthesis of heparan sulfate GAG chains constituents of HSPGs. GPC-1, encoded by the *GPC1* gene (found to be hypomethylated in this study), is typically anchored to the outer surface of the plasma membrane, and functions as a co-receptor for a range of signalling molecules, such as growth factors, morphogens, and chemokines [54]. It modulates signalling pathways associated with cell adhesion, proliferation, motility, migration, survival, or differentiation, such as fibroblast growth factor (FGF), transforming growth factor-beta (TGF-β), BMP-2, VEGF-A, mitogen-activated protein kinase (MAPK), or PI3K/Akt/mTOR and the Wnts/β-catenin pathways [54]. Moreover, GPC-1 may function as a tumor promoter gene in CRC and when mutated promotes the TGF-β1/SMAD2 signalling pathway [54, 55]. The other enriched pathways were mostly related to defects during post-translation O-glycosylation involving sequential enzymatic reactions that catalyse the heparan sulfate chains polymerization, the formation of a tetrasaccharide linker sequence to which the heparan sulfate chains are covalently attached to the core protein [54–56]. Given the structural analogies between CNMs and HS chains, the enrichment of these pathways may suggest a physical interaction between CNMs and GAG or competition between CNMs to GAG ligands to the core protein, impacting GAG regulation. CNMs have been studied as reinforcing materials for tissue engineering or as cell culture nanostructured scaffolds, given their mechanical strength and ECM-mimicking capacity with stimulation of cell adhesion and tissue growth support [57, 58]. Cells cultured attached to nanostructured scaffolds, particularly hydrogels, such as CNMs, were found to proliferate faster and to secrete more ECM-related proteins such as GAGs, than when grown on ordinary scaffolds [57, 58]. On the other hand, excessive GAG production/ storage or deficient lysosomal degradation with increased GAG fragments may lead to autophagy disruption or abnormal interaction of GAG with other cell receptors [59]. GAGs are known to interact with OXTR (which is found to be expressed in the enterocytes), Notably, OXTR mapped to an identified DMR after CMF-ENZ exposure. Abnormal GAG-OXTR interactions drive the formation of large aggregates and leading to OXTR impairment in lysosomal storage diseases, in other cell types [59]. OXTR is involved in gastrointestinal motility, contractility, and epithelium homeostasis, and increased expression has been associated with poor prognosis for colon adenocarcinoma and CRC [59–61].

Cells exposure to CMF-ENZ resulted in enriched DNA repair pathways, with the participation of the *FAAP20* gene encoding the Fanconi anemia core complex associated protein 20, a subunit of a complex that participates in distinct DNA repair mechanisms, including DNA interstrand crosslink repair, TLS of single strand and homology-directed repair of DNA double-strand breaks [62–64]. The TLS enables DNA replication by DNA damage bypass but is error-prone which may lead to point mutation, and damage-inducible mutagenesis [62, 65, 66]. Downregulation of FAAP20 results in loss of TLS (decreased point mutagenesis) and increased chromosome breakage [62, 65, 66]. Our previous study indicated that both CNMs were able to induce DNA damage (single and double DNA strand breaks and alkali-labile sites) in Caco-2 cells, as assessed by the Comet assay, but not chromosomal damage or mutagenicity [3]. However, the effect of *FAAP20* down- or up-regulation on mutagenesis or chromosomal damage would likely be noted if cells were challenged, e.g., with an alkylating agent forming DNA adducts and not directly upon CNMs exposure. Thus, the potential impact of CNMs on this DNA repair mechanism deserves further investigation.

Exposure to CNF-TEMPO enriched pathways related to the Gαs (encoded by the *GNAS gene)* activation and downstream regulation by adenylyl cyclase and activation of protein kinase A (PKA). Enriched pathways were particularly related to Glucagon-like peptides-1 (GLP-1), produced and secreted by enteroendocrine cells (L-cells) in the intestine in response to food consumption, known to also regulate glucose homeostasis [67]. This occurs by enhancing pancreatic insulin secretion, suppressing pancreatic glucagon secretion, slowing gastric emptying and increasing satiety, to lower sugar levels [68–70]. GLP-1 has also been related to the phosphorylation and acetylation of MARCKS, an actin filament crosslinking protein, involved in cell adhesion, polarity, vesicular trafficking, cell motility, and barrier function [71]. GLP-1 is also involved in the downregulation of inflammation pathways, such as the nuclear factor kappa B (NF-κB) [70]. The *GNAS* and *MARCKS* genes were hypermethylated after exposure to CNF-TEMPO and hypomethylated after digestion suggesting a possible modulatory effect of glucose. CNFs have been shown to retard glucose diffusion, due to its viscosity, being studied as functional dietary fibres to modulate the absorption of glucose and gastric emptying [72, 73].

After digestion, DIG CMF-ENZ and DIG CNF-TEMPO did not elicit the enrichment of pathways related to the GAG. However, DIG CNF-TEMPO maintained the same GNAS-enriched pathways as CNF-TEMPO. Both digested CNMs, enriched signalling pathways by RHO GTPase families—RHOA (Common to all undigested and digested CNMs), RHOB, RHOC and RHOQ. These are RHO GTPases activated/deactivated in response to chemical or mechanical stimuli leading to the modulation of downstream effectors, involved in the regulation of cytoskeleton dynamics, cell-cell adhesion, and adhesion to the extracellular matrix, impacting cell polarity, motility, migration, and cell cycle, among other functions important to maintaining cells normal physiology [74–77]. The *PLEKHG5* gene, for instance, involved in the RHOA pathway, has been related to the activation of the canonical TNF-α /nuclear factor kappa B (NF-kB) signalling pathway [78, 79]. RHOB is involved in the modulation of inflammatory intestinal diseases and intestinal cancer, possibly having a protecting role in epithelial integrity and tumor suppressor activity [77]. Reduced RhoA expression has been associated with poor prognosis in colorectal cancer, possibly through the TGF-β and Wnt-β-catenin pathway [80, 81]. In turn, the RHOQ GTPase has been associated with insulin-stimulated glucose uptake possibly downstream of phosphatidylinositol 3-kinase (PI3K) [82]. The RHOA and RHOC (the latter only enriched in DIG CNF-TEMPO) have been reported as reciprocally regulating the epithelial-mesenchymal transition (EMT), with RHOC contributing to CRC invasion and metastatic potential [77, 83]. Two other genes involved in other affected pathways after DIG CNF-TEMPO exposure, *FZD2* and *LOXL2*, have also been associated with EMT [84–86]. FZD2 participates in the Wnt-β-catenin independent pathways (“WNT5A-dependent internalization of FZD2, FZD5 and ROR2”), required for the activation of RAC, a member of the Rho family [87], also possibly implicated in different stages of tumorigenesis [88]. LOXL2 is involved in ECM and cytoskeletal remodelling and stabilization, catalyzing the crosslinking of elastin and collagen, and is regulated by ECM stiffness and increased activation of the PI3K pathway, contributing to intestinal fibrosis and impaired gut motility [89]. LOXL2 has been implicated in tumor progression and poor colon cancer survival [89]. It can influence pro-tumorigenic actions in different tumour contexts by interacting with various receptors such as tyrosine kinase receptor ERBB2, through reactive oxygen species (ROS) generation [85]. *COL23A1*, another gene identified in DMR after exposure to DIG CNF-TEMPO, encodes a transmembrane type 23 collagen protein believed to facilitate cell-cell adhesion and cell‐matrix adhesion during cell migration necessary for intestinal mucosal remodelling, possibly having a role in intestinal chronic inflammation when impaired [90]. Both digested samples enriched pathways related to the ERBB2 tyrosine kinase receptor, but only DIG CNF-TEMPO affected *PIK3R1*. The activation of ERBB2 signalling initiates multiple downstream reactions, including the RAS/mitogen-activated protein kinase (MAPK)-dependent pathway and the PI3K-dependent pathway involved in mitogenesis, apoptosis, cellular motility, and differentiation. *NRG1* encodes for Neuregulin 1, a sulfated glycoprotein ligand for the ERBB family, which interacts with several other components of basement membrane, such as laminin. Dysregulation of the NRG1/ERBB pathway has been associated with a developmental disorder presenting gastrointestinal dysmotility [91]. *PIK3R1* encodes the regulatory subunit of PI3K, being involved in several enriched pathways related to the PI3K/AKT signalling by ERBB, amplifying its regulatory influence.

After exposure to undigested or digested CMF-ENZ, the GO analysis shows molecular function terms related to RNA polymerase II and III and transcription-factor binding, with the various genes found associated with these terms (*POLR1C*,* KDM2B*,* ELF4*,* HOXA9*,* ZBTB45*,* SOX12*,* ZNF280B*,* ZNF664*,* ZNF662*). Some of the genes are associated with DNA methylation machinery, such as the Lysine demethylase 2B, encoded by *KDM2B* that recognizes and binds non-methylated DNA in CGIs, limiting the binding of RNA polymerase II and transcription factors, therefore leading to gene repression [92].

The fact that the global DNA methylation pattern was different between digested and undigested CNMs may be related to physicochemical modifications resulting from CNMs interaction with the digestive fluids, possibly leading to agglomeration or formation of a corona, modulating CNMs contact with the matrix and cells [93–96].

Overall, exposure to both CNMs, either undigested or digested, affected the methylation of genes encoding for cell surface effectors involved in multiple pathways. Both undigested CNMs affected pathways related to glycosylation. CMF-ENZ exposure also affected DNA repair pathways. Undigested or digested CNF-TEMPO impacted pathways involved in GNAS signalling, such as the glucagon and insulin regulation pathways, related to energy metabolism. To clarify the potential implication of CNF-TEMPO on glucose/insulin metabolism, particular focus should be given to genes involved in glucose and insulin-like growth factor receptor binding, such as *GNAS*,* PIK3R1*, or *GLP-1.* All CNMs samples tested seemed to affect pathways implicated in overall cytoskeletal and extracellular matrix dynamics, possibly associated with cell proliferation and motility. However, concerning digested CNMs, particularly DIG CNF-TEMPO, this impact points to a possible deleterious disturbance given the involvement of several ERBB family pathways. Although some of the hypomethylated genes after DIG CNF-TEMPO exposure have been implicated in EMT, this is less probable given the highly variable and intricated pathways potentially involved in these responses [97].

The statistical thresholds implemented for the bioinformatic data analysis represents a balanced, exploratory hypothesis-generating framework focuses on further studies. Given that this study constitutes a screening of CNMs-induced epigenomics, multiple-testing correction was not applied, due to the stringent nature of False Discovery Rate (FDR) corrections, which risked masking biologically relevant target pathways. Therefore, pathways were prioritized based on uncorrected raw *p*-values < 0.05, used solely to rank pathways for qualitative biological interpretation rather than for confirmatory statistical inference. Similarly, a minimum mapping coverage of 3x was retained for CpG filtering to maximize genomic representation in high-throughput RRBS. While these parameters are optimized for exploratory hypothesis generation, this approach may increase the risk of false positives, therefore the results should be interpreted with caution and view as candidate signals requiring subsequent targeted validations.

Further functional studies addressing the affected pathways would be valuable to confirm these results, including targeted gene or protein expression analysis through RT-qPCR, RNA-seq or western-blot. It should be noted that a limitation of the present study is the fact Caco-2 cells are derived from a colon adenocarcinoma. It is recognized that cancer cells undergo widespread demethylation across the genome typically, with hypermethylated CGIs in gene promoter regions [98]. Although the comparison of methylation patterns of CNMs-treated cells with that of non-exposed cells account for the background status of Caco-2 cells, it would be worth confirming the present findings using non-cancer-derived cells.

## Conclusions

The results from this study suggest that CNMs, either directly or after digestion, have a biological effect by inducing changes in DNA methylation in intestinal cells. With the potential expansion of CNMs in the food, our findings highlight that further studies are warranted to disclose their potential biological outcomes and ensure their safety. The new approach methodology herein presented, based on in vitro simulated digestion coupled with epigenomic analysis, provided insights into candidate biological pathways and functions that can be affected and are not captured by the classical toxicity assessment of substances to be used in food. The results obtained helped to better understand the effects of CNMs on the first site of contact upon ingestion and to set the basis for further mechanistic and toxicological research.

## Supplementary Information

Below is the link to the electronic supplementary material.

Supplementary Material 1

## Data Availability

The datasets generated during and/or analysed during the current study are available from the corresponding author on reasonable request.

## Abbreviations

5-mC: 5-Methylcytosine
AKT: AKT serine/threonine kinase
ARHGAP45: Rho GTPase activating protein 45
ARHGEF1: Rho guanine nucleotide exchange factor 1
BP: Biological process
CC: Cellular components
CGIs: CpG islands
CNCs: Cellulose nanocrystals
CNF: Cellulose nanofibrils
CNMs: Cellulose nanomaterials
CMF: Cellulose microfibrils
COL23A1: Collagen type XXIII alpha 1 chain
CpGs: CpG sites
CRC: Colorectal cancer
CSRP1: Cysteine and glycine rich protein 1
DIG: Digested
DMR: Differentially methylated regions
ECACC: European Collection of Authenticated Cell Cultures
ECM: Extracellular matrix
ELF4: E74 like ETS transcription factor 4
EMT: Epithelial-mesenchymal transition
ENZ: Enzymatic
ERBB: erb-b receptor tyrosine kinase
FAAP20: Fanconi anemia core complex-associated protein 20
FGF: Fibroblast growth factor
FZD: Frizzled class receptor
GAG: glycosaminoglycans
GLP-1: Glucagon-like peptide 1
GNAS: GNAS complex locus
GO: Gene Ontology
GPC1: Glypican 1 (gene)
GPC-1: Glypican-1 (protein)
GPCRs: G-protein-coupled receptors
HOXA9: Homeobox A9
HS: Heparan sulfate
HSPGs: Heparan sulfate proteoglycan
HS6ST1: 6-O sulfotransferases
KDM2B: Lysine demethylase 2B
LOXL2: Lysyl oxidase like 2
MAPK: Mitogen-activated protein kinase
MARCKS: Myristoylated alanine rich protein kinase C substrate
MF: Methylation frequency
NMs: Nanomaterials
NID1: Nidogen 1
NRG1: Neuregulin 1
OXTR: Oxytocin receptor
PCA: Principal component analysis
PDE8B: Phosphodiesterase 8B
PIK3R1: Phosphoinositide-3-kinase regulatory subunit 1
PI3K: Phosphoinositide 3-kinase
PKA: Protein kinase A
PLEKHG5: Pleckstrin homology and RhoGEF domain containing G5
POLR1C: RNA polymerase I and III subunit C
RAC: Rac family small GTPase
RGS20: Regulator of G protein signaling 20
RHO: Ras homolog family
RRBS: Reduced Representation Bisulfite Sequencing
SOX12: SRY-box transcription factor 12
TEMPO: 2,2,6,6-tetramethyl-1-piperidinyloxy
TGF-β: Transforming growth factor-beta
TLS: Translesion synthesis
TPM: Transcripts per kilobase million
ZBTB45: Zinc finger and BTB domain containing 45
ZNF280B: Zinc finger protein 280B
ZNF662: Zinc finger protein 662
ZNF664: Zinc finger protein 664

## References

[1] Vital N, Ventura C, Kranendonk M, Silva MJ, Louro H. Toxicological assessment of cellulose nanomaterials: oral exposure. Nanomaterials (Basel). 2022;12(19):3375. 10.3390/nano12193375. 36234501 10.3390/nano12193375 PMC9565252

[2] Brand W, van Kesteren PCE, Swart E, Oomen AG. Overview of potential adverse health effects of oral exposure to nanocellulose. Nanotoxicology. 2022;16(2):217–46. 10.1080/17435390.2022.2069057. 35624082 10.1080/17435390.2022.2069057

[3] Vital N, Cardoso M, Kranendonk M, Silva MJ, Louro H. Evaluation of the cyto- and genotoxicity of two types of cellulose nanomaterials using human intestinal cells and in vitro digestion simulation. Arch Toxicol. 2025;99(2):575–96. 10.1007/s00204-024-03911-2. 39718590 10.1007/s00204-024-03911-2 PMC11775080

[4] Vincentini O, Blier AL, Bogni A, Brun M, Cecchetti S, De Battistis F, Denis S, Etienne-Mesmin L, Ferraris F, Sirio Fumagalli F, Hogeveen K, Iacoponi F, Moracci G, Raggi A, Siciliani L, Stanco D, Verleysen E, Fessard V, Mast J, Blanquet-Diot S, Bremer-Hoffmann S, Cubadda F. EFSA Project on the use of New Approach Methodologies (NAMs) for the hazard assessment of nanofibres. Lot 1, nanocellulose oral exposure: gastrointestinal digestion, nanofibres uptake and local effects. EFSA Supporting Publications. 49 pp. 2023;20(9):EN–8258. 10.2903/sp.efsa.2023.EN-8258.

[5] Italiani P, Paulis M, De Luca AC, Corteggio A, Mangini M, Mantero S, Villa A, Boraschi D, Cassani B. EFSA Pilot Project on NAMs for the hazard assessment of nanofibers. Lot 2: ‘Exploring the use of gut-on‐a‐chip models for risk assessments of nanofibers. External Sci Rep. 2023;20(11):EN-8230. 10.2903/sp.efsa.2023.EN-8230.

[6] Sriraman A, Debnath TK, Xhemalce B, Miller KM. Making it or breaking it: DNA methylation and genome integrity. Essays Biochem. 2020;64(5):687–703. 10.1042/EBC20200009. 32808652 10.1042/EBC20200009 PMC7606623

[7] Bird A. DNA methylation patterns and epigenetic memory. Genes Dev. 2002;16(1):6–21. 10.1101/gad.947102. 11782440 10.1101/gad.947102

[8] Gu J, Stevens M, Xing X, Li D, Zhang B, Payton JE, Oltz EM, Jarvis JN, Jiang K, Cicero T, Costello JF, Wang T. Mapping of variable DNA methylation across multiple cell types defines a dynamic regulatory landscape of the human genome. G3. 2016;6(4):973–86. 10.1534/g3.115.025437 10.1534/g3.115.025437 PMC4825665 26888867

[9] Jones PA. Functions of DNA methylation: islands, start sites, gene bodies and beyond. Nat Rev Genet. 2012;13(7):484–92. 10.1038/nrg3230. 22641018 10.1038/nrg3230

[10] Skvortsova K, Stirzaker C, Taberlay P. The DNA methylation landscape in cancer. Essays Biochem. 2019;63(6):797–811. 10.1042/EBC20190037. 31845735 10.1042/EBC20190037 PMC6923322

[11] Sheaffer KL, Kim R, Aoki R, Elliott EN, Schug J, Burger L, Schübeler D, Kaestner KH. DNA methylation is required for the control of stem cell differentiation in the small intestine. Genes Dev. 2014;28(6):652–64. 10.1101/gad.230318.113. 24637118 10.1101/gad.230318.113 PMC3967052

[12] Wang Y, Wang C, Zhong R, Wang L, Sun L. Research progress of DNA methylation in colorectal cancer (Review). Mol Med Rep. 2024;30(3):154. 10.3892/mmr.2024.13278. 38963030 10.3892/mmr.2024.13278 PMC11240861

[13] Valente A, Vieira L, Silva MJ, Ventura C. The effect of nanomaterials on DNA methylation. Rev Nanomater. 2023;13(12):1880. 10.3390/nano13121880. 10.3390/nano13121880 PMC10305477 37368308

[14] Lu X, Li J, Lou H, Cao Z, Fan X. Genome-wide DNA methylation alterations and potential risk induced by subacute and subchronic exposure to food-grade nanosilica in mice. ACS Nano. 2021;15(7):12449–50. 10.1021/acsnano.1c04677. Erratum for: ACS Nano. 2021;15(5):8225–8243. https://doi.org/10.1021/acsnano.0c07323. 33938728 10.1021/acsnano.0c07323

[15] Moreira L, Costa C, Pires J, Teixeira JP, Fraga S. How can exposure to engineered nanomaterials influence our epigenetic code? A review of the mechanisms and molecular targets. Mutat Res Rev Mutat Res. 2021;788:108385. 10.1016/j.mrrev.2021.108385. 34893164 10.1016/j.mrrev.2021.108385

[16] Pogribna M, Hammons G. Epigenetic effects of nanomaterials and nanoparticles. J Nanobiotechnol. 2021;19(1):2. 10.1186/s12951-020-00740-0. 10.1186/s12951-020-00740-0 PMC7789336 33407537

[17] Le Goff A, Louvel S, Boullier H, Allard P. Toxicoepigenetics for risk assessment: bridging the gap between basic and regulatory science. Epigenet Insights. 2022;15:25168657221113149. 10.1177/25168657221113149. 35860623 10.1177/25168657221113149 PMC9290111

[18] Erdem JS, Závodná T, Ervik TK, Skare Ø, Hron T, Anmarkrud KH, Kuśnierczyk A, Catalán J, Ellingsen DG, Topinka J, Zienolddiny-Narui S. High aspect ratio nanomaterial-induced macrophage polarization is mediated by changes in miRNA levels. Front Immunol. 2023;14:1111123. 10.3389/fimmu.2023.1111123. 36776851 10.3389/fimmu.2023.1111123 PMC9911541

[19] Wreczycka K, Gosdschan A, Yusuf D, Grüning B, Assenov Y, Akalin A. Strategies for analyzing bisulfite sequencing data. J Biotechnol. 2017;261:105–15. 10.1016/j.jbiotec.2017.08.007. 28822795 10.1016/j.jbiotec.2017.08.007

[20] Grunau C, Clark SJ, Rosenthal A. Bisulfite genomic sequencing: systematic investigation of critical experimental parameters. Nucleic Acids Res. 2001;29(13):E65–5. 10.1093/nar/29.13.e65. 11433041 10.1093/nar/29.13.e65 PMC55789

[21] Pinto F, Lourenço AF, Pedrosa JFS, Gonçalves L, Ventura C, Vital N, Bettencourt A, Fernandes SN, da Rosa RR, Godinho MH, Louro H, Ferreira PJT, Silva MJ. Analysis of the in vitro toxicity of nanocelluloses in human lung cells as compared to multi-walled carbon nanotubes. Nanomaterials. 2022;12(9):1432. 10.3390/nano12091432. 35564141 10.3390/nano12091432 PMC9104944

[22] Lourenço AF, Gamelas JAF, Nunes T, Amaral J, Mutjé P, Ferreira PJ. Influence of TEMPO-oxidised cellulose nanofibrils on the properties of filler-containing papers. Cellulose. 2017;24(1):349–62. 10.1007/s10570-016-1121-9.

[23] Lourenço AF, Gamelas JAF, Sarmento P, Ferreira PJT. Enzymatic nanocellulose in papermaking—the key role as filler flocculant and strengthening agent. Carbohydr Polym. 2019;224:115200. 10.1016/j.carbpol.2019.115200. 31472843 10.1016/j.carbpol.2019.115200

[24] Brodkorb A, Egger L, Alminger M, Alvito P, Assunção R, Ballance S, Bohn T, Bourlieu-Lacanal C, Boutrou R, Carrière F, Clemente A, Corredig M, Dupont D, Dufour C, Edwards C, Golding M, Karakaya S, Kirkhus B, Le Feunteun S, Lesmes U, Macierzanka A, Mackie AR, Martins C, Marze S, McClements DJ, Ménard O, Minekus M, Portmann R, Santos CN, Souchon I, Singh RP, Vegarud GE, Wickham MSJ, Weitschies W, Recio I. INFOGEST static in vitro simulation of gastrointestinal food digestion. Nat Protoc. 2019;14(4):991–1014. 10.1038/s41596-018-0119-1. 30886367 10.1038/s41596-018-0119-1

[25] Minekus M, Alminger M, Alvito P, Ballance S, Bohn T, Bourlieu C, Carrière F, Boutrou R, Corredig M, Dupont D, Dufour C, Egger L, Golding M, Karakaya S, Kirkhus B, Le Feunteun S, Lesmes U, Macierzanka A, Mackie A, Marze S, McClements DJ, Ménard O, Recio I, Santos CN, Singh RP, Vegarud GE, Wickham MS, Weitschies W, Brodkorb A. A standardised static in vitro digestion method suitable for food - an international consensus. Food Funct. 2014;5(6):1113–24. 10.1039/c3fo60702j. 24803111 10.1039/c3fo60702j

[26] Vital N, Gramacho AC, Silva M, Cardoso M, Alvito P, Kranendonk M, Silva MJ, Louro H. Challenges of the application of in vitro digestion for nanomaterials safety assessment. Foods. 2024;13(11):1690. 10.3390/foods13111690. 38890918 10.3390/foods13111690 PMC11171843

[27] Veillard AC, Datlinger P, Laczik M, Squazzo S, Bock C. Diagenode^®^ Premium RRBS technology: cost-effective DNA methylation mapping with superior coverage. Nat Methods. 2016;13(2):i–ii. 10.1038/NMETH.F.391.

[28] Olova N, Krueger F, Andrews S, Oxley D, Berrens RV, Branco MR, Reik W. Comparison of whole-genome bisulfite sequencing library preparation strategies identifies sources of biases affecting DNA methylation data. Genome Biol. 2019;20(1):43. 10.1186/s13059-019-1656-9. Erratum for: Genome Biol. 2018;19(1):33. https://doi.org/10.1186/s13059-018-1408-2. 10.1186/s13059-018-1408-2 PMC5856372 29544553

[29] Li Y, Tollefsbol TO. DNA methylation detection: bisulfite genomic sequencing analysis. Methods Mol Biol. 2011;791(3):11–21. 10.1007/978-1-61779-316-5_2 21913068 10.1007/978-1-61779-316-5_2 PMC3233226

[30] Valente A. In vitro effects of titanium dioxide nanoparticles on genome-wide methylation. 2023. 10.1234/56789

[31] Valente A. DNA methylation analysis pipeline v1. 0. Zenodo, 1–5. 2025. https://doi.org/https://doi.org/10.5281/zenodo.14711920. Accessed 7 Jan 2025.

[32] Andrews S. A Quality control tool for high-throughput sequence data (Version 0.12.0). 2023. https://www.bioinformatics.babraham.ac.uk/projects/fastqc/. Accessed 7 July 2024.

[33] Krueger F. Trim galore. Wrapper Tool Cutadapt FastQC Consistently Apply Qual Adapt Trimming FastQ Files. 2021. 10.5281/zenodo.7598955. Accessed 7 July 2024.

[34] Krueger F, Andrews SR, Bismark. A flexible aligner and methylation caller for Bisulfite-Seq applications. Bioinformatics. 2011;27(11):1571–2. 10.1093/bioinformatics/btr167. 21493656 10.1093/bioinformatics/btr167 PMC3102221

[35] Krueger F. Bismark - A tool to map bisulfite converted sequence reads and determine cytosine methylation states. 2019. https://felixkrueger.github.io/Bismark/. Accessed 7 July 2024.

[36] García-Alcalde F, Okonechnikov K, Carbonell J, Cruz LM, Götz S, Tarazona S, Dopazo J, Meyer TF, Conesa A. Qualimap: evaluating next-generation sequencing alignment data. Bioinformatics. 2012;28(20):2678–9. 10.1093/bioinformatics/bts503. 22914218 10.1093/bioinformatics/bts503

[37] Okonechnikov K, Conesa A, García-Alcalde F. Qualimap 2: advanced multi-sample quality control for high-throughput sequencing data. Bioinformatics. 2016;32(2):292–4. . 10.1093/bioinformatics/btv566 26428292 10.1093/bioinformatics/btv566 PMC4708105

[38] Danecek P, Bonfield JK, Liddle J, Marshall J, Ohan V, Pollard MO, Whitwham A, Keane T, McCarthy SA, Davies RM, Li H. Twelve years of SAMtools and BCFtools. Gigascience. 2021;10(2):giab008. 10.1093/gigascience/giab008. 33590861 10.1093/gigascience/giab008 PMC7931819

[39] Ryan D. MethylDackel - A (mostly) universal methylation extractor for BS-seq experiments. 2021. https://github.com/dpryan79/MethylDackel. Accessed 7 July 2024.

[40] Jühling F, Kretzmer H, Bernhart SH, Otto C, Stadler PF, Hoffmann S. Metilene: fast and sensitive calling of differentially methylated regions from bisulfite sequencing data. Genome Res. 2016;26(2):256–62. 10.1101/gr.196394.115. 26631489 10.1101/gr.196394.115 PMC4728377

[41] Quinlan AR, Hall IM. BEDTools: a flexible suite of utilities for comparing genomic features. Bioinformatics. 2010;26(6):841–2. 10.1093/bioinformatics/btq033. 20110278 10.1093/bioinformatics/btq033 PMC2832824

[42] Huang da W, Sherman BT, Lempicki RA. Systematic and integrative analysis of large gene lists using DAVID bioinformatics resources. Nat Protoc. 2009;4(1):44–57. 10.1038/nprot.2008.211. 19131956 10.1038/nprot.2008.211

[43] Sherman BT, Hao M, Qiu J, Jiao X, Baseler MW, Lane HC, Imamichi T, Chang W. DAVID: a web server for functional enrichment analysis and functional annotation of gene lists (2021 update). Nucleic Acids Res. 2022;50(W1):W216–21. 10.1093/nar/gkac194. 35325185 10.1093/nar/gkac194 PMC9252805

[44] Supek F, Bošnjak M, Škunca N, Šmuc T. REVIGO summarizes and visualizes long lists of gene ontology terms. PLoS ONE. 2011;6(7):e21800. 10.1371/journal.pone.0021800. 21789182 10.1371/journal.pone.0021800 PMC3138752

[45] Milacic M, Beavers D, Conley P, Gong C, Gillespie M, Griss J, Haw R, Jassal B, Matthews L, May B, Petryszak R, Ragueneau E, Rothfels K, Sevilla C, Shamovsky V, Stephan R, Tiwari K, Varusai T, Weiser J, Wright A, Wu G, Stein L, Hermjakob H. D’Eustachio P. The Reactome pathway knowledgebase 2024. Nucleic Acids Res. 2024;52(D1):D672–8. 10.1093/nar/gkad1025. 37941124 10.1093/nar/gkad1025 PMC10767911

[46] Boison D, Masino SA, Lubin FD, Guo K, Lusardi T, Sanchez R, Ruskin DN, Ohm J, Geiger JD, Hur J. The impact of methodology on the reproducibility and rigor of DNA methylation data. Sci Rep. 2022;12(1):380. 10.1038/s41598-021-04346-w. 35013473 10.1038/s41598-021-04346-w PMC8748700

[47] Perzel Mandell KA, Eagles NJ, Wilton R, Price AJ, Semick SA, Collado-Torres L, Ulrich WS, Tao R, Han S, Szalay AS, Hyde TM, Kleinman JE, Weinberger DR, Jaffe AE. Genome-wide sequencing-based identification of methylation quantitative trait loci and their role in schizophrenia risk. Nat Commun. 2021;12(1):5251. 10.1038/s41467-021-25517-3. 34475392 10.1038/s41467-021-25517-3 PMC8413445

[48] Zhou J, Xiong Y, Dong X, Wang H, Qian Y, Ma D, Li X. Genome-wide methylation analysis reveals differentially methylated CpG sites and altered expression of heart development-associated genes in fetuses with cardiac defects. Exp Ther Med. 2021;22(3):1032. 10.3892/etm.2021.10464. 34373718 10.3892/etm.2021.10464 PMC8343574

[49] Wickham H. Ggplot2: elegant graphics for data analysis. New York: Springer; 2009. https://ggplot2-book.org/. Accessed 7 July 2024.

[50] Friendly M. Corrgrams-exploratory displays for correlation matrices. Am Stat. 2012;56(4):316–24. 10.1198/000313002533.

[51] Warnes GR, Bolker B, Bonebakker L, Gentleman R, Huber W, Liaw A, Lumley T, Maechler M, Magnusson A, Moeller S. Package ‘gplots’ —various R programming tools for plotting data. 2024. https://rdrr.io/cran/gplots/. Accessed 7 July 2024.

[52] Cavalcante RG, Sartor MA. annotatr: genomic regions in context. Bioinformatics. 2017;33(15):2381–3. 10.1093/bioinformatics/btx183. 28369316 10.1093/bioinformatics/btx183 PMC5860117

[53] Gershman A, Sauria MEG, Guitart X, Vollger MR, Hook PW, Hoyt SJ, Jain M, Shumate A, Razaghi R, Koren S, Altemose N, Caldas GV, Logsdon GA, Rhie A, Eichler EE, Schatz MC, O’Neill RJ, Phillippy AM, Miga KH, Timp W. Epigenetic patterns in a complete human genome. Science. 2022;376(6588):eabj5089. 10.1126/science.abj5089. 35357915 10.1126/science.abj5089 PMC9170183

[54] Pan J, Ho M. Role of glypican-1 in regulating multiple cellular signaling pathways. Am J Physiol Cell Physiol. 2021;321(5):C846–58. 10.1152/ajpcell.00290.2021. 34550795 10.1152/ajpcell.00290.2021 PMC8616591

[55] Lu F, Chen S, Shi W, Su X, Wu H, Liu M. GPC1 promotes the growth and migration of colorectal cancer cells through regulating the TGF-β1/SMAD2 signaling pathway. PLoS ONE. 2022;17(6):e0269094. 10.1371/journal.pone.0269094. 35671267 10.1371/journal.pone.0269094 PMC9173621

[56] Annaval T, Wild R, Crétinon Y, Sadir R, Vivès RR, Lortat-Jacob H. Heparan sulfate proteoglycans biosynthesis and post-synthesis mechanisms combine few enzymes and few core proteins to generate extensive structural and functional diversity. Molecules. 2020;25(18):4215. 10.3390/molecules25184215. 32937952 10.3390/molecules25184215 PMC7570499

[57] Zhou Z, Zhang Y, Zeng Y, Yang D, Mo J, Zheng Z, Zhang Y, Xiao P, Zhong X, Yan W. Effects of nanomaterials on synthesis and degradation of the extracellular matrix. ACS Nano. 2024;18(11):7688–710. 10.1021/acsnano.3c09954. 38436232 10.1021/acsnano.3c09954

[58] Jovic TH, Nicholson T, Arora H, Nelson K, Doak SH, Whitaker IS. A comparative analysis of pulp-derived nanocelluloses for 3D bioprinting facial cartilages. Carbohydr Polym. 2023;321:121261. 10.1016/j.carbpol.2023.121261. 37739492 10.1016/j.carbpol.2023.121261

[59] Pierzynowska K, Gaffke L, Żabińska M, Cyske Z, Rintz E, Wiśniewska K, Podlacha M, Węgrzyn G. Roles of the oxytocin receptor (OXTR) in human diseases. Int J Mol Sci. 2023;24(4):3887. 10.3390/ijms24043887. 36835321 10.3390/ijms24043887 PMC9966686

[60] Liu H, Yang G, Wang H. Oxytocin/oxytocin receptor signalling in the gastrointestinal system: mechanisms and therapeutic potential. Int J Mol Sci. 2024;25(20):10935. 10.3390/ijms252010935. 39456718 10.3390/ijms252010935 PMC11508134

[61] Danhof HA, Lee J, Thapa A, Britton RA, Di Rienzi SC. Microbial stimulation of oxytocin release from the intestinal epithelium via secretin signaling. Gut Microbes. 2023;15(2):2256043. 10.1080/19490976.2023.2256043. 37698879 10.1080/19490976.2023.2256043 PMC10498800

[62] Kim H, Yang K, Dejsuphong D, D’Andrea AD. Regulation of Rev1 by the Fanconi anemia core complex. Nat Struct Mol Biol. 2012;19(2):164–70. 10.1038/nsmb.2222. 22266823 10.1038/nsmb.2222 PMC3280818

[63] Ma X, Tang TS, Guo C. Regulation of translesion DNA synthesis in mammalian cells. Environ Mol Mutagen. 2020;61(7):680–92. 10.1002/em.22359. 31983077 10.1002/em.22359

[64] Palovcak A, Yuan F, Verdun R, Luo L, Zhang Y. Fanconi anemia-associated protein 20 (FAAP20) plays an essential role in homology-directed repair of DNA double-strand breaks. Commun Biol. 2023;6(1):873. 10.1038/s42003-023-05252-9. 37620397 10.1038/s42003-023-05252-9 PMC10449828

[65] Nayak S, Calvo JA, Cong K, Peng M, Berthiaume E, Jackson J, Zaino AM, Vindigni A, Hadden MK, Cantor SB. Inhibition of the translesion synthesis polymerase REV1 exploits replication gaps as a cancer vulnerability. Sci Adv. 2020;6(24):eaaz7808. 10.1126/sciadv.aaz7808. 32577513 10.1126/sciadv.aaz7808 PMC7286678

[66] Tonzi P, Huang TT. Role of Y-family translesion DNA polymerases in replication stress: Implications for new cancer therapeutic targets. DNA Repair. 2019;78:20–6. 10.1016/j.dnarep.2019.03.016. 30954011 10.1016/j.dnarep.2019.03.016 PMC6534436

[67] Nadkarni P, Chepurny OG, Holz GG. Regulation of glucose homeostasis by GLP-1. Prog Mol Biol Transl Sci. 2014;121:23–65. 10.1016/B978-0-12-800101-1.00002-8. 24373234 10.1016/B978-0-12-800101-1.00002-8 PMC4159612

[68] Spreckley E, Murphy KG. The L-cell in nutritional sensing and the regulation of appetite. Front Nutr. 2015;2:23. 10.3389/fnut.2015.00023. 26258126 10.3389/fnut.2015.00023 PMC4507148

[69] Zhang J, Zheng Y, Martens L, Pfeiffer AFH. The regulation and secretion of glucagon in response to nutrient composition: unraveling their intricate mechanisms. Nutrients. 2023;15(18):3913. 10.3390/nu15183913. 37764697 10.3390/nu15183913 PMC10536047

[70] Mehdi SF, Pusapati S, Anwar MS, Lohana D, Kumar P, Nandula SA, Nawaz FK, Tracey K, Yang H, LeRoith D, Brownstein MJ, Roth J. Glucagon-like peptide-1: a multi-faceted anti-inflammatory agent. Front Immunol. 2023;14:1148209. 10.3389/fimmu.2023.1148209. 37266425 10.3389/fimmu.2023.1148209 PMC10230051

[71] Van Itallie CM, Tietgens AJ, Aponte A, Gucek M, Cartagena-Rivera AX, Chadwick RS, Anderson JM. MARCKS-related protein regulates cytoskeletal organization at cell–cell and cell–substrate contacts in epithelial cells. J Cell Sci. 2018;131(3):jcs210237. 10.1242/jcs.210237. 29222109 10.1242/jcs.210237 PMC5826046

[72] Liu L, Kerr WL, Kong F, Dee DR, Lin M. Influence of nano-fibrillated cellulose (NFC) on starch digestion and glucose absorption. Carbohydr Polym. 2018;196:146–53. 10.1016/j.carbpol.2018.04.116. 29891281 10.1016/j.carbpol.2018.04.116

[73] Liu L, Kong F. In vitro investigation of the influence of nano-cellulose on starch and milk digestion and mineral adsorption. Int J Biol Macromol. 2019a;137:1278–85. 10.1016/j.ijbiomac.2019.06.194. 31271795 10.1016/j.ijbiomac.2019.06.194

[74] Huang JP, Lin J, Tzen CY, Huang WY, Tsai CC, Chen CJ, Lu YJ, Chou KF, Su YW. FANCA D1359Y mutation in a patient with gastric polyposis and cancer susceptibility: a case report and review of literature. World J Gastroenterol. 2018;24(38):4412–8. 10.3748/wjg.v24.i38.4412. 30344425 10.3748/wjg.v24.i38.4412 PMC6189845

[75] Iwatake M, Nishishita K, Okamoto K, Tsukuba T. The Rho-specific guanine nucleotide exchange factor Plekhg5 modulates cell polarity, adhesion, migration, and podosome organization in macrophages and osteoclasts. Exp Cell Res. 2017;359(2):415–30. 10.1016/j.yexcr.2017.08.025. 28847484 10.1016/j.yexcr.2017.08.025

[76] Hetmanski JHR, de Belly H, Busnelli I, Waring T, Nair RV, Sokleva V, Dobre O, Cameron A, Gauthier N, Lamaze C, Swift J, Del Campo A, Starborg T, Zech T, Goetz JG, Paluch EK, Schwartz JM, Caswell PT. Membrane tension orchestrates rear retraction in matrix-directed cell migration. Dev Cell. 2019;51(4):460–e47510. 10.1016/j.devcel.2019.09.006. 31607653 10.1016/j.devcel.2019.09.006 PMC6863396

[77] Pradhan R, Ngo PA, Martínez-Sánchez LD, Neurath MF, López-Posadas R. Rho GTPases as Key Molecular Players within Intestinal Mucosa and GI Diseases. Cells. 2021;10(1):66. 10.3390/cells10010066. 33406731 10.3390/cells10010066 PMC7823293

[78] Cordero RY, Cordero JB, Stiemke AB, Datta LW, Buyske S, Kugathasan S, McGovern DPB, Brant SR, Simpson CL. Trans-ancestry, Bayesian meta-analysis discovers 20 novel risk loci for inflammatory bowel disease in an African American, East Asian and European cohort. Hum Mol Genet. 2023;32(5):873–82. 10.1093/hmg/ddac269. 36308435 10.1093/hmg/ddac269 PMC9941836

[79] Tong L, Tergaonkar V. Rho protein GTPases and their interactions with NFκB: crossroads of inflammation and matrix biology. Biosci Rep. 2014;34(3):e00115. 10.1042/BSR20140021. 24877606 10.1042/BSR20140021 PMC4069681

[80] Dopeso H, Rodrigues P, Bilic J, Bazzocco S, Cartón-García F, Macaya I, de Marcondes PG, Anguita E, Masanas M, Jiménez-Flores LM, Martínez-Barriocanal Á, Nieto R, Segura MF, Schwartz S Jr, Mariadason JM, Arango D. Mechanisms of inactivation of the tumour suppressor gene RHOA in colorectal cancer. Br J Cancer. 2018;118(1):106–16. 10.1038/bjc.2017.420. 29206819 10.1038/bjc.2017.420 PMC5765235

[81] Rodrigues P, Macaya I, Bazzocco S, Mazzolini R, Andretta E, Dopeso H, Mateo-Lozano S, Bilić J, Cartón-García F, Nieto R, Suárez-López L, Afonso E, Landolfi S, Hernandez-Losa J, Kobayashi K, Ramón S, Tabernero J, Tebbutt NC, Mariadason JM, Schwartz S, Arango D. RHOA inactivation enhances Wnt signalling and promotes colorectal cancer. Nat Commun. 2014;5:5458. . 10.1038/ncomms6458 25413277 10.1038/ncomms6458 PMC4255233

[82] Møller LLV, Klip A, Sylow L. Rho GTPases-emerging regulators of glucose homeostasis and metabolic health. Cells. 2019;8(5):434. 10.3390/cells8050434. 31075957 10.3390/cells8050434 PMC6562660

[83] Thomas P, Pranatharthi A, Ross C, Srivastava S. RhoC: a fascinating journey from a cytoskeletal organizer to a cancer stem cell therapeutic target. J Experimental Clin Cancer Research: CR. 2019;38(1):328. 10.1186/s13046-019-1327-4. 10.1186/s13046-019-1327-4 PMC6651989 31340863

[84] Zhou M, Sun X, Zhu Y. Analysis of the role of Frizzled 2 in different cancer types. FEBS Open Bio. 2021;11(4):1195–208. 10.1002/2211-5463.13111. 33565732 10.1002/2211-5463.13111 PMC8016138

[85] Cano A, Eraso P, Mazón MJ, Portillo F. LOXL2 in cancer: a two-decade perspective. Int J Mol Sci. 2023;24(18):14405. 10.3390/ijms241814405. 37762708 10.3390/ijms241814405 PMC10532419

[86] Wen B, Xu LY, Li EM. LOXL2 in cancer: regulation, downstream effectors and novel roles. Biochim Biophys Acta Rev Cancer. 2020;1874(2):188435. 10.1016/j.bbcan.2020. 32976981 10.1016/j.bbcan.2020.188435

[87] Chen Y, Chen Z, Tang Y, Xiao Q. The involvement of noncanonical Wnt signaling in cancers. Biomed Pharmacother. 2021;133:110946. 10.1016/j.biopha.2020.110946. 33212376 10.1016/j.biopha.2020.110946

[88] Mack NA, Whalley HJ, Castillo-Lluva S, Malliri A. The diverse roles of Rac signaling in tumorigenesis. Cell Cycle. 2011;10(10):1571–81. 10.4161/cc.10.10.15612. 21478669 10.4161/cc.10.10.15612 PMC3127158

[89] Torres S, Garcia-Palmero I, Herrera M, Bartolomé RA, Peña C, Fernandez-Aceñero MJ, Padilla G, Peláez-García A, Lopez-Lucendo M, Rodriguez-Merlo R, García de Herreros A, Bonilla F, Casal JI. LOXL2 is highly expressed in cancer-associated fibroblasts and associates to poor colon cancer survival. Clin Cancer Res. 2015;21(21):4892–902. 10.1158/1078-0432.CCR-14-3096. 26206869 10.1158/1078-0432.CCR-14-3096

[90] Manon-Jensen T, Sun S, Lindholm M, Domislović V, Giuffrida P, Brinar M, Mazza G, Pinzani M, Krznarić Z, Di Sabatino A, Karsdal MA, Mortensen JH. Elevated ectodomain of type 23 collagen is a novel biomarker of the intestinal epithelium to monitor disease activity in ulcerative colitis and Crohn’s disease. United Eur Gastroenterol J. 2021;9(2):268–78. 10.1177/2050640620977371. 10.1177/2050640620977371 PMC8259268 33351719

[91] Le TL, Galmiche L, Levy J, Suwannarat P, Hellebrekers DM, Morarach K, Boismoreau F, Theunissen TE, Lefebvre M, Pelet A, Martinovic J, Gelot A, Guimiot F, Calleroz A, Gitiaux C, Hully M, Goulet O, Chardot C, Drunat S, Capri Y, Bole-Feysot C, Nitschké P, Whalen S, Mouthon L, Babcock HE, Hofstra R, de Coo IF, Tabet AC, Molina TJ, Keren B, Brooks A, Smeets HJ, Marklund U, Gordon CT, Lyonnet S, Amiel J, Bondurand N. Dysregulation of the NRG1/ERBB pathway causes a developmental disorder with gastrointestinal dysmotility in humans. J Clin Invest. 2021;131(6):e145837. 10.1172/JCI145837. 33497358 10.1172/JCI145837 PMC7954599

[92] Farcas AM, Blackledge NP, Sudbery I, Long HK, McGouran JF, Rose NR, Lee S, Sims D, Cerase A, Sheahan TW, Koseki H, Brockdorff N, Ponting CP, Kessler BM, Klose RJ. KDM2B links the Polycomb Repressive Complex 1 (PRC1) to recognition of CpG islands. Elife. 2012;11:e00205. 10.7554/eLife.00205. 10.7554/eLife.00205 PMC3524939 23256043

[93] Lin YJ, Shatkin JA, Kong F. Evaluating mucoadhesion properties of three types of nanocellulose in the gastrointestinal tract in vitro and ex vivo. Carbohydr Polym. 2019;210:157–66. 10.1016/j.carbpol.2019.01.029. 30732748 10.1016/j.carbpol.2019.01.029

[94] Liu L, Kong F. Influence of nanocellulose on in vitro digestion of whey protein isolate. Carbohydr Polym. 2019;210:399–411. 10.1016/j.carbpol.2019.01.071. 30732777 10.1016/j.carbpol.2019.01.071

[95] Duan Y, Coreas R, Liu Y, Bitounis D, Zhang Z, Parviz D, Strano M, Demokritou P, Zhong W. Prediction of protein corona on nanomaterials by machine learning using novel descriptors. NanoImpact. 2020;17:10. 10.1016/j.impact.2020.100207. 10.1016/j.impact.2020.100207 PMC7043407 32104746

[96] Coreas R, Cao X, Deloid GM, Demokritou P, Zhong W. Lipid and protein corona of food-grade TiO_2_ nanoparticles in simulated gastrointestinal digestion. NanoImpact. 2020;20:100272. 10.1016/j.impact.2020.100272. 33344797 10.1016/j.impact.2020.100272 PMC7742882

[97] Cook DP, Vanderhyden BC. Context specificity of the EMT transcriptional response. Nat Commun. 2020;11(1):2142. 10.1038/s41467-020-16066-2. 32358524 10.1038/s41467-020-16066-2 PMC7195456

[98] Reddington JP, Sproul D, Meehan RR. DNA methylation reprogramming in cancer: does it act by re-configuring the binding landscape of Polycomb repressive complexes? BioEssays. 2014;36(2):134–40. 10.1002/bies.201300130. 24277643 10.1002/bies.201300130 PMC4225474

