# Supplementary material for: The effects of ingested cellulose nanomaterials on DNA methylation in intestinal cells
Source: Part Fibre Toxicol. 2026 Mar 12;23:37. doi: 10.1186/s12989-026-00672-x (PMC13335175; doi:10.1186/s12989-026-00672-x)
Supplement: Supplementary file 1 — Supplementary Material 1 [file 12989_2026_672_MOESM1_ESM.docx]

**Supplementary File**

**The effects of ingested cellulose nanomaterials on DNA methylation in intestinal cells**

Nádia Vital^1,2,3^, Célia Ventura^1,3^, Camila Fernandes^1^, Luís Vieira^1,3^, Ana Valente^1,3^, Michel Kranendonk^2,3^, Maria João Silva^1,3*^, Henriqueta Louro^1,3^

1 Department of Human Genetics, National Institute of Health Dr. Ricardo Jorge (INSA),

Lisbon, Portugal

2 NOVA Medical School, Universidade NOVA de Lisboa, Lisbon, Portugal

3 Comprehensive Health Research Centre (CHRC), NOVA Medical School, Universidade NOVA de Lisboa, Lisbon, Portugal

* Corresponding author:

Maria João Silva

Department of Human Genetics, Research and Development Unit

National Institute of Health Doutor Ricardo Jorge

Avenida Padre Cruz, 1649-016 Lisboa, PORTUGAL

TEL +351 217 519 234

[m.joao.silva@insa.min-saude.pt](mailto:m.joao.silva@insa.min-saude.pt)

**Quality Control**

**
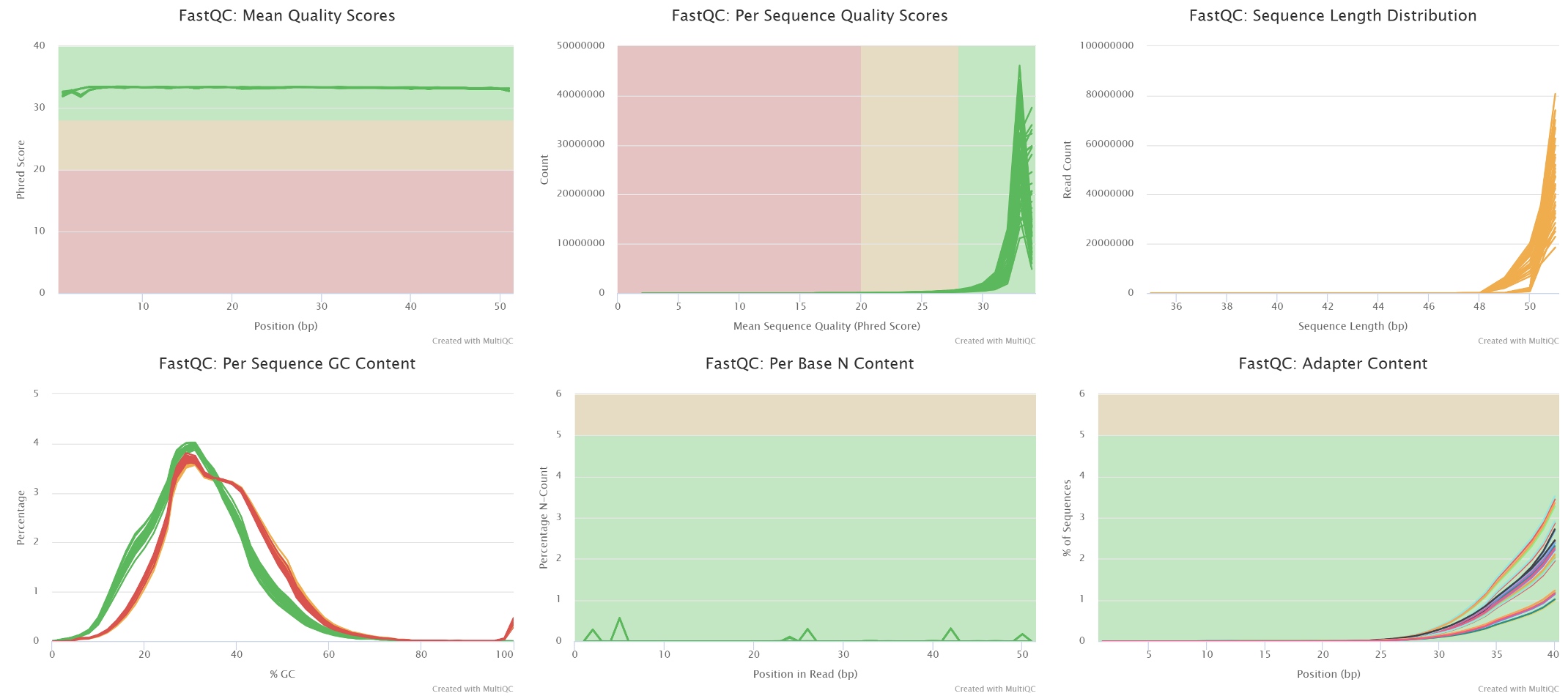
**

**Fig. S1** Sequencing quality analyses using FASTQC quality control tool.

|  | **BAMQC Report** | | | | | | | | | | | | |
| --- | --- | --- | --- | --- | --- | --- | --- | --- | --- | --- | --- | --- | --- |
|  | **Reference T2T genome** | | | | | | | **Reference T2T genome and GpG islands bed file** | | | | | |
| Sample ID | Mapped reads | Duplication  (%) | Coverage  Mean | Mapping  Quality | Insert size | | error rate  (%) | Mapped reads | Duplication (%) | Coverage | Mapping Quality | Insert size | error rate  (%) |
| Control R1 | 117926046 | 80,02 | 1,8238 | 37,31 | 133 | 27,84 | | 36350628 | 87,03 | 64,6167 | 39,59 | 96 | 34,46 |
| Control R2 | 130234986 | 79,93 | 2,0273 | 37,22 | 150 | 27,02 | | 32935064 | 87,27 | 59,0191 | 39,56 | 110 | 33,86 |
| Control R3 | 105520706 | 80,24 | 1,6265 | 37,18 | 133 | 27,44 | | 30871481 | 87,56 | 54,3841 | 39,61 | 94 | 34,01 |
| CMF-ENZ R1 | 43415662 | 81,49 | 0,6718 | 37,46 | 146 | 27,42 | | 12750108 | 88,39 | 22,5739 | 40,04 | 105 | 34,24 |
| CMF-ENZ R2 | 76735344 | 80,86 | 1,1947 | 37,13 | 162 | 26,12 | | 15537876 | 88,72 | 27,6682 | 39,85 | 117 | 33,06 |
| CMF-ENZ R3 | 81319394 | 80,08 | 1,2667 | 36,85 | 167 | 25,92 | | 14255892 | 87,89 | 25,3292 | 39,89 | 119 | 32,88 |
| CNF-TEMPO R1 | 69347880 | 80,23 | 1,0739 | 37,06 | 151 | 26,77 | | 16121235 | 87,31 | 28,4487 | 39,88 | 104 | 33,65 |
| CNF-TEMPO R2 | 108075676 | 80,18 | 1,6838 | 36,98 | 155 | 26,43 | | 22679430 | 87,99 | 40,5696 | 39,71 | 112 | 33,45 |
| CNF-TEMPO R3 | 93979060 | 80,33 | 1,4514 | 37,02 | 134 | 27,20 | | 24550124 | 87,74 | 43,3154 | 39,67 | 95 | 33,88 |
| DIG Control R1 | 58512522 | 80,31 | 0,9077 | 36,89 | 146 | 26,31 | | 12122456 | 88,32 | 21,451 | 39,99 | 103 | 33,05 |
| DIG Control R2 | 87284520 | 79,79 | 1,352 | 36,94 | 151 | 26,40 | | 18275316 | 87,54 | 32,1685 | 39,8 | 103 | 33,32 |
| DIG Control R3 | 99239792 | 80,72 | 1,5369 | 37,02 | 139 | 26,90 | | 25427590 | 88,30 | 45,0703 | 39,67 | 100 | 33,57 |
| DIG CMF-ENZ R1 | 113045620 | 80,25 | 1,7506 | 37,29 | 139,29 | 27,44 | | 32375382 | 87,28 | 57,5359 | 39,64 | 100,59 | 34,17 |
| DIG CMF-ENZ R2 | 71245562 | 81,46 | 1,1028 | 37,3 | 145,1 | 27,16 | | 11292297 | 87,33 | 19,9585 | 39,99 | 105,55 | 33,44 |
| DIG CMF-ENZ R3 | 119055802 | 79,71 | 1,8471 | 37,11 | 151 | 26,73 | | 27770996 | 87,19 | 49,2454 | 39,62 | 106 | 33,75 |
| DIG CNF-TEMPO R1 | 56190768 | 79,91 | 0,8658 | 37,18 | 141 | 27,22 | | 14941722 | 87,14 | 26,1774 | 39,92 | 96 | 33,97 |
| DIG CNF-TEMPO R2 | 62651160 | 81,16 | 0,9671 | 37 | 122 | 27,46 | | 18396534 | 88,58 | 32,6242 | 39,86 | 90 | 33,75 |
| DIG CNF-TEMPO R3 | 51590602 | 79,74 | 0,8 | 37,04 | 152 | 26,54 | | 11292297 | 87,33 | 19,9585 | 39,99 | 106 | 33,44 |

**Table S1.** *BAMQC Report*

**Table S2.** *Number of CpG sites per replicate of undigested and digested CNMs samples*

| **Sample** | **Undigested** | | **Digested** | |
| --- | --- | --- | --- | --- |
|  | **Filter for CpGs islands** | **Filter CpG common matrix (coverage 3x)** | **Filter for CpGs islands** | **Filter CpG common matrix (coverage 3x)** |
| Control Replicate 1 | 1302622 | 989353 | 1204962 | 1015823 |
| Control Replicate 2 | 1287391 |  | 1237456 |  |
| Control Replicate 3 | 1267854 |  | 1250393 |  |
| CMF-ENZ Replicate 1 | 1208442 |  | 1308959 |  |
| CMF-ENZ Replicate 2 | 1201315 |  | 1263381 |  |
| CMF-ENZ Replicate 3 | 1217852 |  | 1205047 |  |
| CNF-TEMPO Replicate 1 | 1245238 |  | 1241415 |  |
| CNF-TEMPO Replicate 2 | 1239487 |  | 1202606 |  |
| CNF-TEMPO Replicate 3 | 1244965 |  | 1217224 |  |

**Correlation and PCA analysis**


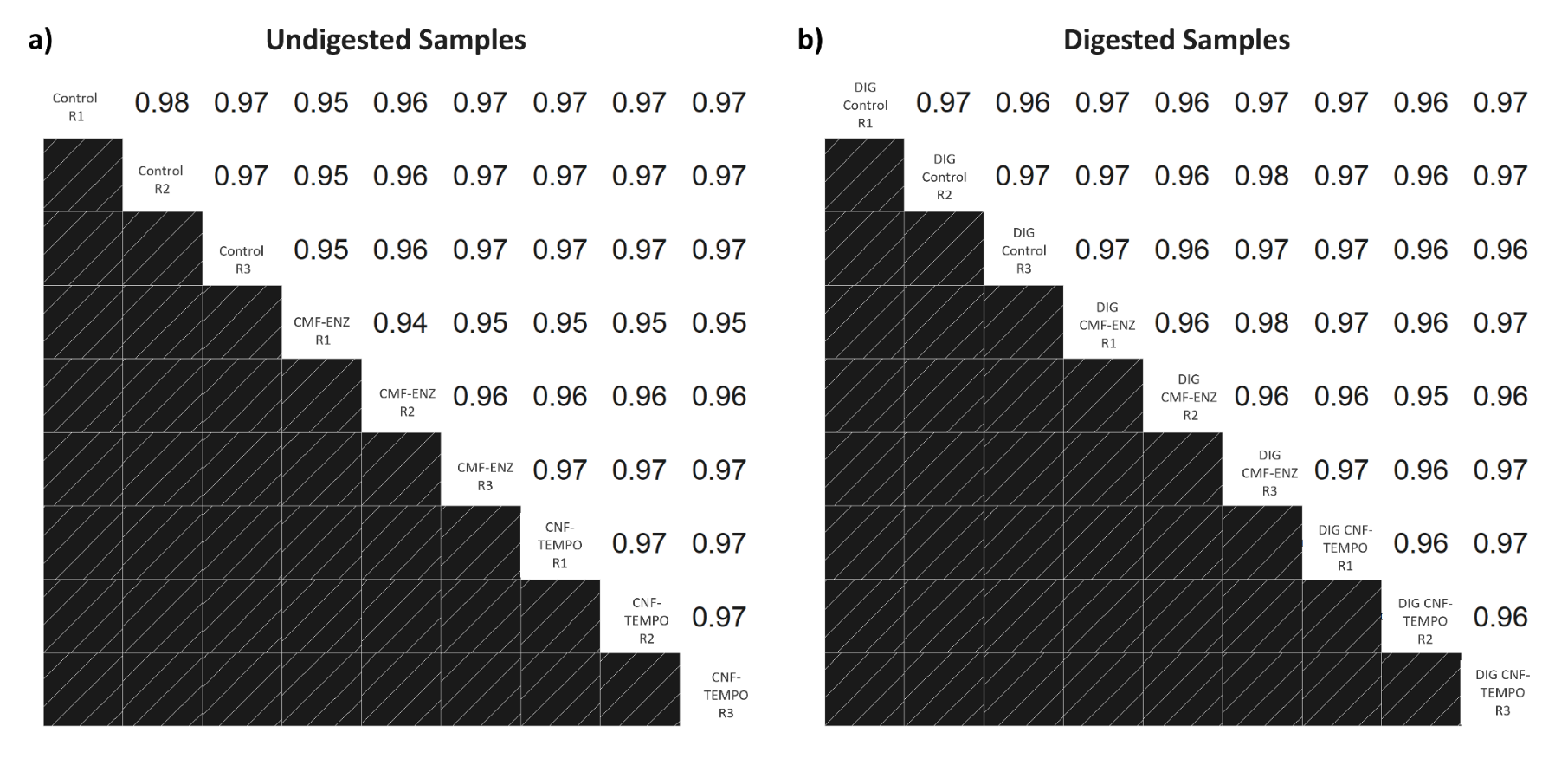


**Fig. S2** Replicate correlation analysis between all samples using Correlogram in a) undigested and b) digested samples


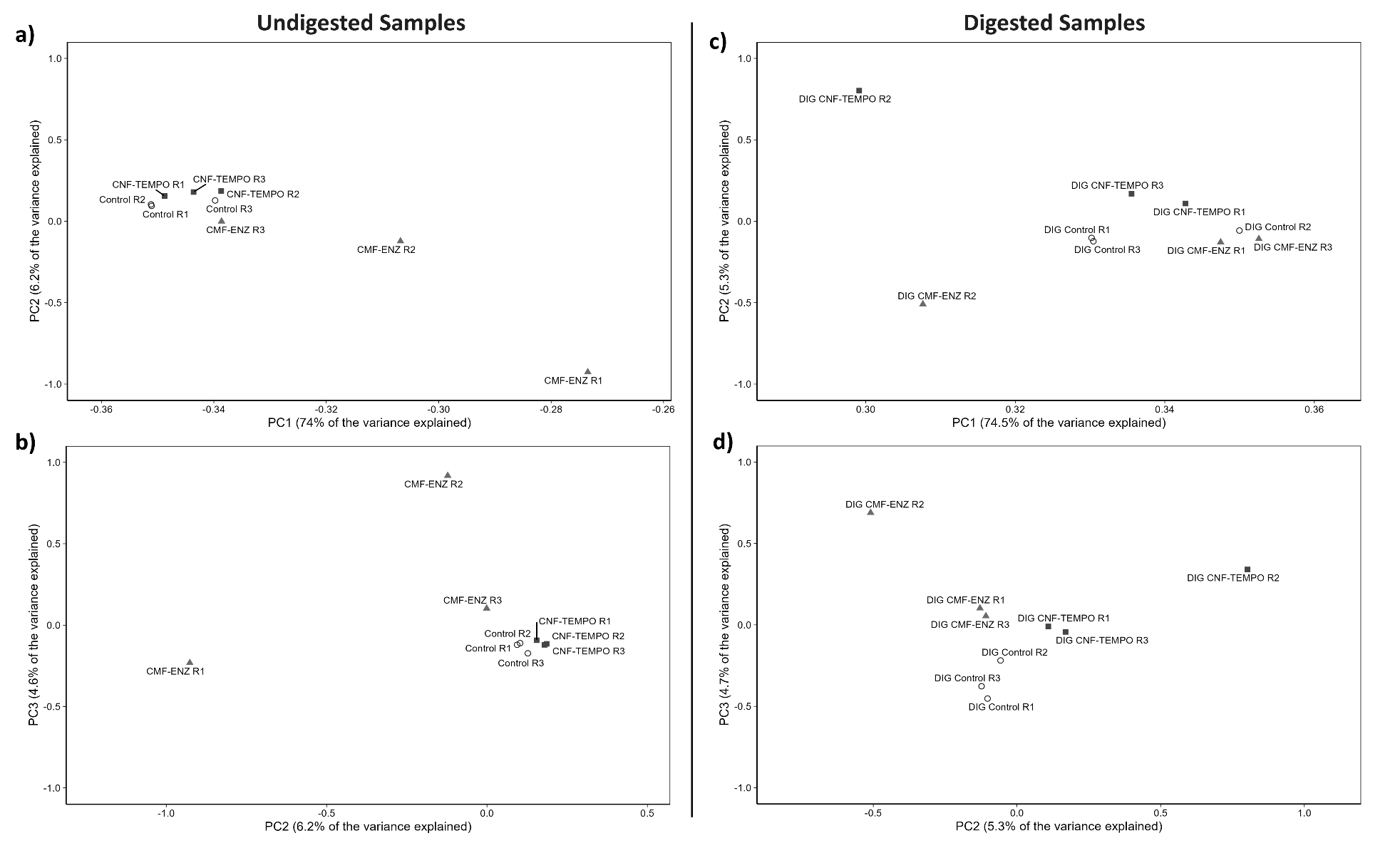


**Fig. S3** Principal components analysis of PC1 and PC2 for a) undigested and b) digested samples; D) Principal components analysis of PC2 and PC3 for c) undigested and d) digested samples.

**Table S3.** *Principal component analysis (PCA) scores and importance of components*

|  | **Undigested samples** | | | **Digested samples** | | |
| --- | --- | --- | --- | --- | --- | --- |
|  | PC1 | PC2 | PC3 | PC1 | PC2 | PC3 |
| Control R1 | -0.3510722 | 0.0939979 | -0.1207613 | 0.3302068 | -0.1022836 | -0.4525036 |
| Control R2 | -0.3511724 | 0.1034562 | -0.1107913 | 0.3499842 | -0.0571154 | -0.2185452 |
| Control R3 | -0.3397711 | 0.1275126 | -0.1730404 | 0.3304557 | -0.1232022 | -0.3768587 |
| CMF-ENZ R1 | -0.2734779 | -0.9265646 | -0.2317638 | 0.3474712 | -0.1286266 | 0.1023115 |
| CMF-ENZ R2 | -0.3067992 | -0.1223592 | 0.9178397 | 0.3076515 | -0.5091992 | 0.6887252 |
| CMF-ENZ R3 | -0.3386298 | -0.0009495 | 0.1027816 | 0.3525809 | -0.1081118 | 0.0540595 |
| CNF-TEMPO R1 | -0.3487322 | 0.1549340 | -0.0929300 | 0.3427783 | 0.1090334 | -0.0097392 |
| CNF-TEMPO R2 | -0.3386949 | 0.1858805 | -0.1149741 | 0.2991313 | 0.8019761 | 0.3402396 |
| CNF-TEMPO R3 | -0.3436095 | 0.1793102 | -0.1209784 | 0.3355563 | 0.1688979 | -0.0431930 |
|  | **Importance of components** | | | | | |
| Standard deviation | 2.580181 | 0.7447601 | 0.6464533 | 0.6899822 | 0.6485637 | 0.5461254 |
| Proportion of variance | 0.739700 | 0.0616300 | 0.0464300 | 0.745310 | 0.0529000 | 0.0467400 |
| Cumulative variation | 0.739700 | 0.8013300 | 0.8477700 | 0.745310 | 0.7982100 | 0.8449400 |

**CpG sites**

**
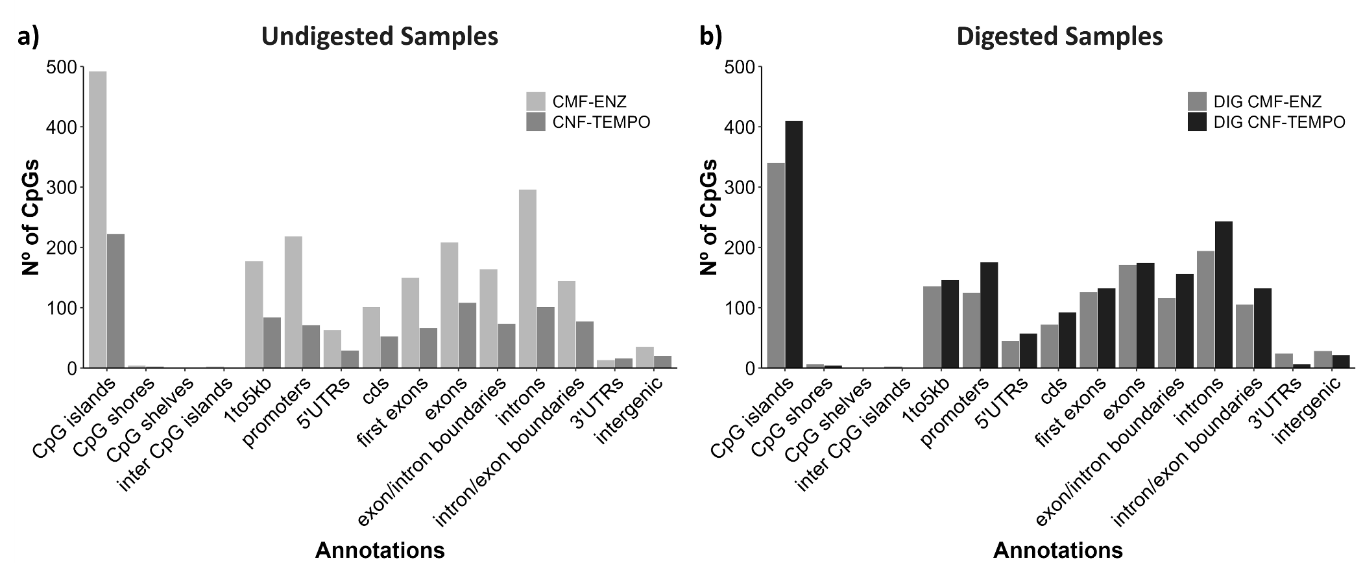
**

**Fig.  *S4*** Distribution of CpG sites along the genomic annotations for undigested and digested samples.

**Differentially methylated regions and functional analysis**

**Table S4.** *Differentially methylated regions (DMR) containing genes after exposure to CMF-ENZ*

| **Chromosome** | **DMR Start** | **DMR End** | **Methylation difference** | **Gene name** | **Distance** |
| --- | --- | --- | --- | --- | --- |
| chr1 | 1640449 | 1640492 | 43.766667 | *FAAP20* | 0 |
| chr1 | 24160100 | 24160465 | 30.083333 | *GRHL3* | 0 |
| chr1 | 235460809 | 235460907 | 29.400000 | *NID1* | 0 |
| chr1 | 235461167 | 235461399 | 18.629630 | *NID1* | 0 |
| chr1 | 235461528 | 235461745 | 16.363636 | *NID1* | -126 |
| chr1 | 5997060 | 5997186 | 20.604167 | *PLEKHG5* | 0 |
| chr1* | 160577724 | 160577914 | 10.366667 | *TRNAD-GUC* | 0 |
| chr2* | 131469462 | 131469602 | 17.098039 | *ARHGEF4* | 0 |
| chr2* | 172581271 | 172581497 | 17.280702 | *DLX2* | 4185 |
| chr2 | 240946446 | 240946530 | -12.250000 | *GPC1* | 0 |
| chr2 | 240946446 | 240946530 | -12.250000 | *GPC1-AS1* | 0 |
| chr2* | 90934721 | 90934805 | 21.520833 | *IGKV1OR2-118* | -7989 |
| chr2* | 74506621 | 74507010 | 21.795699 | *LBX2* | 0 |
| chr2 | 220055574 | 220055664 | 56.404762 | *OBSL1* | 0 |
| chr2* | 85439720 | 85439831 | 16.222222 | *SH2D6* | -675 |
| chr2* | 131469462 | 131469602 | 17.098039 | *SMIM39* | 0 |
| chr3 | 8759307 | 8759384 | 33.944444 | *OXTR* | 0 |
| chr3* | 145710848 | 145710952 | 18.365079 | *PAQR9* | 0 |
| chr3* | 139559747 | 139560095 | 17.460317 | *SLC35G2* | 0 |
| chr4 | 86438670 | 86439161 | 23.228070 | *PLAC8* | 0 |
| chr5 | 78112431 | 78112510 | 26.250000 | *OTP* | 0 |
| chr6* | 43834468 | 43834554 | 11.859649 | *LINC03040* | 0 |
| chr6 | 43834468 | 43834554 | 11.859649 | *POLR1C* | 0 |
| chr6* | 43834468 | 43834554 | 11.859649 | *SCIRT* | 0 |
| chr6* | 27627101 | 27627163 | 29.266667 | *TRV-AAC1-5* | -3938 |
| chr7* | 27301150 | 27301388 | 12.733333 | *HOXA10-HOXA9* | 0 |
| chr7 | 27301150 | 27301388 | 12.733333 | *HOXA9* | 0 |
| chr8* | 42170252 | 42170415 | 25.194444 | *ANK1* | 0 |
| chr8* | 100064963 | 100065231 | 21.638889 | *LOC112268016* | 0 |
| chr8 | 144162346 | 144162399 | 44.366667 | *LY6E* | 0 |
| chr8 | 144727130 | 144727196 | 20.566667 | *NAPRT* | 0 |
| chr8* | 32829642 | 32829812 | 18.724638 | *NRG1* | 0 |
| chr8 | 100064963 | 100065231 | 21.638889 | *STK3* | 0 |
| chr9* | 110880384 | 110880501 | 6.424242 | *GABBR2* | 0 |
| chr9* | 140907489 | 140907565 | 27.422222 | *PKN3* | 0 |
| chr9* | 140907666 | 140907759 | 17.128205 | *PKN3* | 0 |
| chr9* | 150415595 | 150415676 | 12.547619 | *TUBBP5* | 0 |
| chr11 | 64261597 | 64261703 | 18.200000 | *BAD* | 0 |
| chr11 | 64261597 | 64261703 | 18.200000 | *GPR137* | 0 |
| chr11* | 119189385 | 119189480 | 25.566667 | *NLRX1* | 0 |
| chr12 | 24818947 | 24819108 | 14.033333 | *BCAT1* | 0 |
| chr12 | 121531002 | 121531164 | 14.000000 | *KDM2B* | 0 |
| chr14* | 95941319 | 95941406 | 39.200000 | *LINC02320* | 0 |
| chr15* | 87532280 | 87532493 | 30.166667 | *MESP2* | 0 |
| chr16* | 25968958 | 25969013 | 26.833333 | *HS3ST4* | 0 |
| chr16 | 64259252 | 64259309 | 28.700000 | *NDRG4* | 0 |
| chr17 | 82027725 | 82027962 | 31.583333 | *AATK* | 0 |
| chr17* | 41643898 | 41644095 | 30.947368 | *KRT27* | 0 |
| chr17 | 33768670 | 33768868 | 13.492063 | *MYO1D* | 0 |
| chr18* | 74684612 | 74684795 | -6.470588 | *DIPK1C* | 0 |
| chr19* | 1438181 | 1438261 | 16.700000 | *APC2* | 0 |
| chr19 | 1031571 | 1031702 | 15.476190 | *ARHGAP45* | 0 |
| chr19* | 42311269 | 42311642 | 19.383838 | *DLL3* | 0 |
| chr19 | 50574188 | 50574433 | 15.541667 | *EHD2* | -243 |
| chr19* | 38301089 | 38301229 | 26.187500 | *HSPB6* | 0 |
| chr19 | 42112131 | 42112401 | 20.641975 | *LRFN1* | 0 |
| chr19* | 14598943 | 14599497 | 26.724138 | *PTGER1* | 0 |
| chr19 | 61610147 | 61610256 | 14.177778 | *ZBTB45* | 0 |
| chr20 | 5951793 | 5952012 | -7.166667 | *CHGB* | 0 |
| chr20 | 61717720 | 61718061 | 9.752688 | *FAM217B* | 0 |
| chr20* | 37322484 | 37322742 | 19.047619 | *FER1L4* | 0 |
| chr20* | 45247909 | 45248126 | 14.526316 | *L3MBTL1* | 0 |
| chr20* | 37322484 | 37322742 | 19.047619 | *LOC124904891* | 0 |
| chr20 | 370736 | 370843 | 19.900000 | *SOX12* | 0 |
| chr20* | 61717720 | 61718061 | 9.752688 | *SYCP2* | 0 |
| chr22 | 24618091 | 24618342 | 27.400000 | *CABIN1* | 0 |
| chr22* | 20842131 | 20842210 | 23.900000 | *SCARF2* | -35 |
| chr22* | 43824001 | 43824200 | 20.275362 | *SCUBE1* | 0 |
| chr22* | 29141228 | 29141413 | 28.911111 | *TTC28* | 0 |
| chrX | 128429910 | 128429972 | 40.933333 | *ELF4* | 0 |
| chrX* | 128429910 | 128429972 | 40.933333 | *RAB33A* | 0 |

*Removed after filter with Caco-2.

**Table S5.** *Differentially methylated regions (DMR) containing genes after exposure to DIG CMF-ENZ*

| **Chromosome** | **DMR Start** | **DMR End** | **Methylation difference** | **Gene name** | **Distance** |
| --- | --- | --- | --- | --- | --- |
| chr1 | 153462845 | 153462898 | -37.133333 | *ATP8B2* | 0 |
| chr1 | 159037203 | 159037281 | -26.966667 | *CFAP45* | 0 |
| chr1 | 151312417 | 151312829 | -23.877193 | *FLG-AS1* | 42 |
| chr1 | 60932659 | 60932909 | -26.407407 | *NFIA* | 23111 |
| chr1 | 235461122 | 235461249 | -18.547619 | *NID1* | 0 |
| chr1 | 235461528 | 235461745 | -12.075758 | *NID1* | -126 |
| chr1 | 227567521 | 227567698 | -28.019608 | *OBSCN* | 0 |
| chr1 | 152673216 | 152673319 | -17.055556 | *S100A6* | 0 |
| chr1* | 23258816 | 23258977 | 10.388889 | *TCEA3* | 0 |
| chr1 | 35436566 | 35436608 | -29.233333 | *TFAP2E* | 0 |
| chr1 | 35436566 | 35436608 | -29.233333 | *TFAP2E-AS1* | 0 |
| chr1* | 160577724 | 160577914 | -16.200000 | *TRNAD-GUC* | 0 |
| chr2* | 218853776 | 218854150 | -17.950000 | *CATIP-AS1* | 0 |
| chr2 | 218853776 | 218854150 | -17.950000 | *CATIP* | 0 |
| chr2 | 55220682 | 55220910 | -15.214286 | *CLHC1* | 0 |
| chr2* | 119281158 | 119281356 | -16.861111 | *EN1* | -151 |
| chr2 | 9499626 | 9499822 | -9.606061 | *IAH1* | 0 |
| chr2* | 90934568 | 90934752 | -20.075758 | *IGKV1OR2-118* | -7836 |
| chr2* | 74524739 | 74524785 | 17.800000 | *TLX2* | 0 |
| chr2 | 70912387 | 70912760 | -13.412281 | *VAX2* | 0 |
| chr3 | 190557520 | 190557612 | 17.500000 | *BCL6* | 0 |
| chr3 | 2093475 | 2093663 | -25.400000 | *CNTN4* | 0 |
| chr3* | 187145109 | 187145186 | 39.102564 | *FAM131A* | 1 |
| chr3* | 44014890 | 44015009 | -24.537037 | *LOC124909489* | 0 |
| chr3 | 46908840 | 46908892 | -14.366667 | *PTH1R* | 0 |
| chr3 | 42921593 | 42921689 | -21.566667 | *ZNF662* | 35 |
| chr4 | 111475272 | 111475519 | -27.600000 | *LEF1-AS1* | 0 |
| chr5 | 179142174 | 179142269 | -21.433333 | *COL23A1* | 0 |
| chr5 | 78112381 | 78112823 | -20.750000 | *OTP* | 0 |
| chr5 | 36940147 | 36940345 | -8.410256 | *SLC1A3-AS1* | 0 |
| chr6 | 90326646 | 90326722 | -30.333333 | *SRSF12* | 0 |
| chr7 | 45735218 | 45735295 | -36.666667 | *ADCY1* | 0 |
| chr7 | 21679029 | 21679175 | -12.512821 | *DNAH11* | 10 |
| chr7* | 27301150 | 27301328 | 13.611111 | *HOXA10-HOXA9* | 0 |
| chr7 | 27301150 | 27301328 | 13.611111 | *HOXA9* | 0 |
| chr7* | 66458090 | 66458294 | 9.416667 | *LOC124906646* | 8886 |
| chr7 | 157804518 | 157804850 | -14.047619 | *RNF32-DT* | 0 |
| chr7 | 3415690 | 3415796 | -18.833333 | *SDK1* | 0 |
| chr7 | 3415690 | 3415796 | -18.833333 | *SDK1-AS1* | 0 |
| chr7 | 29093567 | 29093852 | -34.739130 | *TRIL* | 0 |
| chr7 | 29094110 | 29094535 | -30.491228 | *TRIL* | 0 |
| chr8 | 144162218 | 144162311 | -18.933333 | *LY6E* | 0 |
| chr8 | 32830488 | 32830675 | -17.794872 | *NRG1* | 0 |
| chr8 | 31192935 | 31193245 | -16.393939 | *TEX15* | 0 |
| chr9 | 110880716 | 110880765 | -30.133333 | *GABBR2* | -122 |
| chr9 | 36280503 | 36280559 | -20.400000 | *GNE* | 0 |
| chr9 | 38448232 | 38448344 | -15.916667 | *IGFBPL1* | 0 |
| chr9* | 133566054 | 133566212 | -34.500000 | *LOC102723324* | 0 |
| chr9* | 36280503 | 36280559 | -20.400000 | *LOC124902150* | 0 |
| chr9* | 149368138 | 149368423 | -36.222222 | *LOC124902353* | -6662 |
| chr9 | 101340979 | 101341157 | -15.770833 | *SHC3* | 0 |
| chr9 | 133566054 | 133566212 | -34.500000 | *STOM* | 0 |
| chr9 | 150415561 | 150415676 | -9.291667 | *TUBBP5* | 0 |
| chr10 | 87640826 | 87640898 | -3.422222 | *BMPR1A* | 0 |
| chr10 | 132922052 | 132922516 | -20.580645 | *BNIP3* | 0 |
| chr10 | 11753585 | 11754002 | -13.404762 | *ECHDC3* | 0 |
| chr10 | 118429745 | 118429873 | -26.395833 | *EMX2OS* | 0 |
| chr10 | 102662630 | 102662719 | -30.666667 | *FGF8* | 0 |
| chr10* | 100417292 | 100417517 | 15.539683 | *NKX2-3* | 0 |
| chr11 | 46503714 | 46503820 | -14.947368 | *DGKZ* | 0 |
| chr11 | 111987781 | 111987857 | -14.358974 | *DIXDC1* | 0 |
| chr11 | 74396853 | 74397019 | -21.766667 | *KCNE3* | 0 |
| chr11 | 602393 | 602540 | -27.800000 | *LMNTD2* | 0 |
| chr11* | 67109686 | 67109780 | -31.400000 | *LOC107984341* | 0 |
| chr11 | 126448139 | 126448259 | -14.833333 | *ST3GAL4* | -2064 |
| chr11 | 45443824 | 45443916 | -27.166667 | *SYT13* | 0 |
| chr11* | 76165149 | 76165574 | -21.194444 | *TRP-AGG2-4* | 0 |
| chr11* | 76165149 | 76165574 | -21.194444 | *TRP-TGG2-1* | 0 |
| chr12 | 6657717 | 6657854 | -23.583333 | *ACRBP* | 0 |
| chr12 | 1792660 | 1792935 | -11.400000 | *CACNA2D4* | 0 |
| chr12 | 1793489 | 1793636 | -22.214286 | *CACNA2D4* | 0 |
| chr12 | 132938029 | 132938260 | -24.981481 | *CHFR* | 0 |
| chr12 | 53939060 | 53939169 | -19.466667 | *HOTAIR* | 0 |
| chr12 | 53939060 | 53939169 | -19.466667 | *HOXC11* | 0 |
| chr12* | 70029 | 70121 | -51.250000 | *IQSEC3* | 0 |
| chr12 | 121531064 | 121531160 | -13.492063 | *KDM2B* | 0 |
| chr12* | 116489592 | 116489700 | -14.921569 | *LOC105370008* | 0 |
| chr12* | 132959255 | 132959644 | -21.222222 | *LOC124903065* | 0 |
| chr12 | 51778168 | 51778271 | -15.923077 | *TMDD1* | 0 |
| chr12 | 123974760 | 123974789 | -13.000000 | *ZNF664* | 0 |
| chr12* | 123974760 | 123974789 | -13.000000 | *ZNF664-RFLNA* | 0 |
| chr13 | 42241918 | 42242032 | -20.000000 | *DNAJC15* | 0 |
| chr14 | 90270521 | 90270815 | -13.000000 | *C14orf132* | 0 |
| chr14 | 99331978 | 99332102 | -25.400000 | *LINC02298* | 6445 |
| chr14 | 18514620 | 18514977 | -20.954955 | *LTB4R* | 0 |
| chr14 | 17084856 | 17085226 | -14.449275 | *REM2* | 0 |
| chr15* | 20429651 | 20429944 | -30.526316 | *LOC283683* | 0 |
| chr15 | 87532505 | 87532681 | -15.933333 | *MESP2* | 0 |
| chr15 | 62877298 | 62877420 | -23.396825 | *RASL12* | 0 |
| chr15 | 74218474 | 74218563 | -25.800000 | *SCAPER* | 505 |
| chr15 | 73496029 | 73496111 | -23.566667 | *SNUPN* | 0 |
| chr16 | 3332804 | 3333020 | -32.933333 | *ZNF75A* | 0 |
| chr17 | 2613694 | 2613787 | -10.666667 | *CCDC92B* | 0 |
| chr17 | 45411419 | 45411528 | -32.857143 | *FZD2* | 0 |
| chr17 | 82301929 | 82302004 | -23.512821 | *LINC03048* | -1383 |
| chr17* | 59624077 | 59624294 | -26.435897 | *LOC105371843* | 0 |
| chr17 | 59624077 | 59624294 | -26.435897 | *PPM1E* | 0 |
| chr17 | 29313872 | 29313953 | -28.800000 | *SARM1* | 0 |
| chr17 | 62265935 | 62266097 | -32.391304 | *TBX2-AS1* | 0 |
| chr18 | 23813771 | 23813939 | -39.355556 | *ANKRD29* | 0 |
| chr19 | 1478389 | 1478530 | -22.119048 | *ADAMTSL5* | 0 |
| chr19 | 44702605 | 44702666 | -26.200000 | *ARHGEF1* | 6 |
| chr19 | 44702867 | 44703042 | -28.733333 | *ARHGEF1* | 0 |
| chr19 | 17161694 | 17161811 | -32.666667 | *CPAMD8* | 0 |
| chr19 | 53563940 | 53564140 | -17.526316 | *EMC10* | 0 |
| chr19 | 53563940 | 53564140 | -17.526316 | *GARIN5A* | 0 |
| chr19* | 52385531 | 52385638 | -23.352941 | *LOC101928295* | -21 |
| chr19* | 44343974 | 44344043 | -27.250000 | *PLEKHA3P1* | -275 |
| chr19 | 48326034 | 48326311 | -31.083333 | *PPM1N* | 0 |
| chr19 | 48326034 | 48326311 | -31.083333 | *RTN2* | 0 |
| chr19* | 33869376 | 33869524 | -23.907407 | *TSHZ3-AS1* | 577 |
| chr20 | 6811339 | 6811574 | -20.688889 | *BMP2* | 0 |
| chr20 | 61717443 | 61718061 | -21.157895 | *FAM217B* | 0 |
| chr20 | 25650185 | 25650215 | 16.000000 | *NINL* | 0 |
| chr20 | 61717443 | 61718061 | -21.157895 | *SYCP2* | 0 |
| chr22* | 22931525 | 22931722 | -24.138889 | *IGL* | 0 |
| chr22 | 42145826 | 42145929 | -26.800000 | *XRCC6* | -2810 |
| chr22 | 22931525 | 22931722 | -24.138889 | *ZNF280B* | 0 |
| chrX | 128429946 | 128430009 | -28.523810 | *ELF4* | 0 |
| chrX | 128429946 | 128430009 | -28.523810 | *RAB33A* | 0 |

*removed after filter with Caco-2.

**Table S6.** *Differentially methylated regions (DMR) containing genes after exposure to CNF-TEMPO*

| **Chromosome** | **DMR Start** | **DMR End** | **Methylation difference** | **Gene name** | **Distance** |
| --- | --- | --- | --- | --- | --- |
| chr1 | 200764789 | 200764914 | 6.214286 | *CSRP1* | 0 |
| chr1* | 58693014 | 58693230 | 19.650794 | *JUN-DT* | 0 |
| chr1* | 58693014 | 58693230 | 19.650794 | *LOC124905711* | 0 |
| chr1 | 235461167 | 235461361 | 16.229167 | *NID1* | 0 |
| chr1 | 235461590 | 235461683 | 17.487179 | *NID1* | -188 |
| chr1* | 227567521 | 227567562 | 55.866667 | *OBSCN* | 0 |
| chr1 | 152673283 | 152673402 | 17.500000 | *S100A6* | -37 |
| chr2* | 96031853 | 96032035 | 21.066667 | *ANKRD33BP1* | 0 |
| chr2 | 88181768 | 88181882 | 14.435897 | *FABP1* | 0 |
| chr2* | 96031853 | 96032035 | 21.066667 | *GPAT2* | 0 |
| chr2 | 240946471 | 240946530 | -12.051282 | *GPC1* | 0 |
| chr2 | 240946471 | 240946530 | -12.051282 | *GPC1-AS1* | 0 |
| chr2 | 128754157 | 128754296 | 31.254902 | *HS6ST1* | 0 |
| chr2 | 9499397 | 9499774 | 9.508772 | *IAH1* | 0 |
| chr2* | 90934709 | 90934805 | 21.313725 | *IGKV1OR2-118* | -7977 |
| chr2* | 27758547 | 27758827 | 13.160920 | *LINC01460* | 0 |
| chr2* | 96031853 | 96032035 | 21.066667 | *LINC03052* | 0 |
| chr2 | 220055235 | 220055370 | 25.175439 | *OBSL1* | 0 |
| chr2* | 85439756 | 85439874 | 14.025641 | *SH2D6* | -711 |
| chr2 | 88181768 | 88181882 | 14.435897 | *THNSL2* | 0 |
| chr2* | 74525099 | 74525206 | 22.366667 | *TLX2* | 0 |
| chr2* | 42816541 | 42816696 | 17.200000 | *TRI-TAT2-1* | -344 |
| chr3* | 139560006 | 139560126 | 16.700000 | *SLC35G2* | 0 |
| chr5* | 11841659 | 11841786 | 13.537037 | *CTNND2* | 0 |
| chr5* | 44437 | 44479 | 20.700000 | *LOC105374602* | -2850 |
| chr5 | 77692815 | 77692850 | -15.933333 | *PDE8B* | 0 |
| chr6* | 80045475 | 80045699 | 14.866667 | *IRAK1BP1* | 0 |
| chr6* | 43834468 | 43834604 | 17.303030 | *LINC03040* | 0 |
| chr6 | 115044162 | 115044398 | 39.800000 | *MARCKS* | 0 |
| chr6 | 43834468 | 43834604 | 17.303030 | *POLR1C* | 0 |
| chr6* | 43834468 | 43834604 | 17.303030 | *SCIRT* | 0 |
| chr7* | 35395124 | 35395247 | 17.933333 | *TBX20* | 0 |
| chr8 | 61137927 | 61138032 | 12.033333 | *CHD7* | 0 |
| chr8* | 33870842 | 33870966 | 25.100000 | *DUSP26* | 0 |
| chr8* | 23678468 | 23678594 | 33.133333 | *LOXL2* | 0 |
| chr8 | 145692526 | 145692682 | 15.555556 | *LRRC14* | 0 |
| chr8 | 145692759 | 145692868 | -12.300000 | *LRRC14* | 0 |
| chr8* | 145692526 | 145692682 | 15.555556 | *LRRC24* | 0 |
| chr8* | 145692759 | 145692868 | -12.300000 | *LRRC24* | 0 |
| chr8 | 144727619 | 144727727 | 29.358974 | *NAPRT* | 0 |
| chr8* | 54258456 | 54258576 | 22.600000 | *RGS20* | 0 |
| chr8 | 54259461 | 54259637 | -29.900000 | *RGS20* | 0 |
| chr8* | 31193133 | 31193321 | 8.500000 | *TEX15* | 0 |
| chr9* | 33542494 | 33542699 | 29.666667 | *ANKRD18B* | 54 |
| chr9* | 149657692 | 149657754 | 12.277778 | *EXD3* | 0 |
| chr9* | 147941289 | 147941348 | 6.866667 | *KCNT1* | 0 |
| chr9* | 140135026 | 140135216 | 20.025641 | *PIP5KL1* | 0 |
| chr9* | 140907428 | 140907764 | 11.087302 | *PKN3* | 0 |
| chr9* | 101340853 | 101341005 | 25.954545 | *SHC3* | 0 |
| chr10 | 134232372 | 134232532 | 12.882353 | *ADAM8* | 0 |
| chr10* | 49373367 | 49373443 | 11.527778 | *ARHGAP22* | 0 |
| chr10* | 102663085 | 102663468 | 45.066667 | *FGF8* | 0 |
| chr10 | 93568860 | 93569131 | 10.944444 | *HHEX* | 472 |
| chr10* | 28029864 | 28030055 | 36.974359 | *ODAD2* | 0 |
| chr10* | 111556259 | 111556354 | 12.738095 | *RBM20* | 0 |
| chr11 | 65586630 | 65586760 | 23.372549 | *EHBP1L1* | 0 |
| chr12 | 125057558 | 125057677 | 18.923077 | *BRI3BP* | 0 |
| chr12* | 1792784 | 1792991 | 11.383333 | *CACNA2D4* | 0 |
| chr12 | 121531178 | 121531354 | 13.476190 | *KDM2B* | 0 |
| chr12* | 65708518 | 65708799 | 23.833333 | *RPSAP52* | 28702 |
| chr13* | 22116681 | 22116772 | 22.142857 | *LINC00621* | 0 |
| chr13* | 22116681 | 22116772 | 22.142857 | *NUS1P2* | 0 |
| chr14* | 99736675 | 99736731 | 13.733333 | *CRIP1* | 0 |
| chr14* | 99331978 | 99332102 | 24.222222 | *LINC02298* | 6445 |
| chr14* | 17084845 | 17085226 | 19.652778 | *REM2* | 0 |
| chr15* | 64443922 | 64443997 | 21.000000 | *LINC01169* | 0 |
| chr15* | 4701958 | 4702030 | 14.566667 | *LOC124907653* | 2820 |
| chr15* | 20429741 | 20429934 | 16.692308 | *LOC283683* | 0 |
| chr15* | 71744599 | 71744792 | 9.771930 | *LOXL1* | 0 |
| chr15* | 71744599 | 71744792 | 9.771930 | *LOXL1-AS1* | 0 |
| chr15* | 87532453 | 87532608 | 13.636364 | *MESP2* | 0 |
| chr15 | 62877105 | 62877299 | 19.871795 | *RASL12* | 0 |
| chr15 | 62877300 | 62877511 | 9.011905 | *RASL12* | 0 |
| chr16 | 10421813 | 10421883 | 24.733333 | *ATF7IP2* | 0 |
| chr16* | 52586954 | 52587246 | 18.791667 | *LOC100130180* | 0 |
| chr17 | 29313908 | 29313993 | 22.363636 | *SARM1* | 0 |
| chr19 | 1031625 | 1031714 | 13.333333 | *ARHGAP45* | 0 |
| chr19 | 44702867 | 44703076 | 18.923077 | *ARHGEF1* | 0 |
| chr19 | 44759475 | 44759611 | 15.041667 | *ARHGEF1* | -10052 |
| chr19 | 58744198 | 58744408 | 21.789474 | *CCDC106* | 0 |
| chr19 | 42112198 | 42112295 | 17.454545 | *LRFN1* | 0 |
| chr19* | 53784092 | 53784270 | 15.000000 | *SHANK1* | 0 |
| chr19 | 61610133 | 61610221 | 18.564103 | *ZBTB45* | 0 |
| chr20 | 61717942 | 61718074 | 19.785714 | *FAM217B* | 0 |
| chr20 | 60673468 | 60673558 | 16.642857 | *GNAS* | 0 |
| chr20* | 45240938 | 45241102 | 15.121212 | *L3MBTL1* | 0 |
| chr20* | 45247909 | 45248126 | 18.859649 | *L3MBTL1* | 0 |
| chr20 | 25650062 | 25650178 | 21.897436 | *NINL* | 0 |
| chr20 | 65883012 | 65883211 | 15.666667 | *TCEA2* | 0 |
| chr20 | 65883448 | 65883530 | 13.861111 | *TCEA2* | 0 |
| chr20* | 33914152 | 33914328 | 17.866667 | *TSPY26P* | 0 |
| chr22 | 24618110 | 24618342 | 25.770833 | *CABIN1* | 0 |
| chr22* | 22931397 | 22931496 | 15.642857 | *IGL* | 0 |
| chr22* | 22931525 | 22931714 | 10.606061 | *IGL* | 0 |
| chr22* | 22931397 | 22931496 | 15.642857 | *ZNF280B* | 0 |
| chr22* | 22931525 | 22931714 | 10.606061 | *ZNF280B* | 0 |
| chrX* | 2541714 | 2541801 | 32.777778 | *ARSD* | 0 |
| chrX | 48302108 | 48302226 | 34.487179 | *TIMM17B* | 1703 |

*removed after filter with Caco-2.

**Table S7.** *Differentially methylated regions (DMR) containing genes after exposure to DIG CNF-TEMPO*

| **Chromosome** | **DMR Start** | **DMR End** | **Methylation difference** | **Gene name** | **Distance** |
| --- | --- | --- | --- | --- | --- |
| chr1 | 44684714 | 44685012 | -23.683333 | *BTBD19* | 0 |
| chr1* | 160236021 | 160236221 | 20.666667 | *KLHDC9* | 0 |
| chr2 | 73306349 | 73306425 | -19.633333 | *EGR4* | 0 |
| chr2* | 119281158 | 119281356 | -16.361111 | *EN1* | -151 |
| chr2 | 20704359 | 20704405 | -23.266667 | *GDF7* | 0 |
| chr2 | 96831931 | 96832082 | -25.309524 | *ITPRIPL1* | 0 |
| chr2* | 206756525 | 206756679 | 22.800000 | *ZDBF2* | 0 |
| chr2 | 206756696 | 206756780 | -20.555556 | *ZDBF2* | 0 |
| chr3 | 2092592 | 2092674 | -23.410256 | *CNTN4* | 99 |
| chr3 | 2093475 | 2093663 | -17.466667 | *CNTN4* | 0 |
| chr3* | 187145109 | 187145186 | 36.846154 | *FAM131A* | 1 |
| chr3* | 44014890 | 44014939 | -19.566667 | *LOC124909489* | 0 |
| chr3* | 44015019 | 44015078 | -18.266667 | *LOC124909489* | 0 |
| chr3 | 46862468 | 46862567 | -18.866667 | *MYL3* | 11704 |
| chr3* | 139559606 | 139559790 | 9.176471 | *SLC35G2* | 0 |
| chr5 | 179142172 | 179142262 | -18.600000 | *COL23A1* | 0 |
| chr5* | 77692986 | 77693115 | 22.642857 | *PDE8B* | 0 |
| chr5 | 69110827 | 69110907 | -25.033333 | *PIK3R1* | 0 |
| chr5 | 36940147 | 36940345 | -8.692308 | *SLC1A3-AS1* | 0 |
| chr6 | 115044397 | 115044443 | -31.500000 | *MARCKS* | 0 |
| chr7 | 21679169 | 21679459 | -20.074074 | *DNAH11* | 0 |
| chr7 | 27305412 | 27305961 | -16.692308 | *HOXA10-AS* | 0 |
| chr7* | 27305412 | 27305961 | -16.692308 | *HOXA10-HOXA9* | 0 |
| chr7* | 521025 | 521193 | -24.800000 | *LOC116435278* | 3797 |
| chr7* | 66458157 | 66458294 | 10.500000 | *LOC124906646* | 8886 |
| chr7* | 27305412 | 27305961 | -16.692308 | *MIR196B* | 0 |
| chr7 | 157804248 | 157804759 | -22.358974 | *RNF32-DT* | 0 |
| chr7 | 29093589 | 29093782 | -34.196078 | *TRIL* | 0 |
| chr7 | 29093829 | 29094059 | -19.651515 | *TRIL* | 0 |
| chr8 | 145697482 | 145697624 | -24.755556 | *C8orf82* | 0 |
| chr8 | 23678655 | 23678908 | -35.270833 | *LOXL2* | 0 |
| chr8 | 144426052 | 144426223 | 18.333333 | *MINCR* | 1325 |
| chr8 | 144727552 | 144727683 | -27.425926 | *NAPRT* | 0 |
| chr8 | 32830416 | 32830552 | -24.200000 | *NRG1* | 0 |
| chr9* | 109393254 | 109393594 | 14.075758 | *ANKRD18CP* | 0 |
| chr9 | 110880675 | 110880796 | -17.238095 | *GABBR2* | -81 |
| chr9 | 36280209 | 36280512 | -19.533333 | *GNE* | 0 |
| chr9 | 36280519 | 36280583 | -9.212121 | *GNE* | 0 |
| chr9* | 36280209 | 36280512 | -19.533333 | *LOC124902150* | 0 |
| chr9* | 36280519 | 36280583 | -9.212121 | *LOC124902150* | 0 |
| chr9 | 140907671 | 140907726 | -23.033333 | *PKN3* | 0 |
| chr9 | 147926310 | 147926466 | -28.766667 | *SOHLH1* | 0 |
| chr10 | 118429832 | 118429991 | -33.700000 | *EMX2OS* | 0 |
| chr10 | 134379408 | 134379483 | -33.755556 | *SPRN* | 0 |
| chr10 | 102023676 | 102023778 | -23.433333 | *TLX1NB* | 0 |
| chr11 | 553341 | 553414 | -20.888889 | *RNH1* | -326 |
| chr11 | 93120585 | 93120674 | -8.800000 | *SLC36A4* | 0 |
| chr12* | 127156556 | 127156627 | -16.435897 | *LOC105370062* | 16330 |
| chr13 | 42241918 | 42242032 | -16.633333 | *DNAJC15* | 0 |
| chr13* | 22116489 | 22116677 | -23.700000 | *LINC00621* | 0 |
| chr13* | 22116489 | 22116677 | -23.700000 | *NUS1P2* | 0 |
| chr13* | 93914903 | 93914980 | -25.473684 | *SOX21* | 0 |
| chr14* | 2811861 | 2811947 | 5.589744 | *LOC124907457* | 3301 |
| chr14 | 17084834 | 17085042 | -29.277778 | *REM2* | 0 |
| chr15 | 62877166 | 62877372 | -19.383333 | *RASL12* | 0 |
| chr16 | 3470611 | 3470804 | -20.606061 | *ZNF597* | 0 |
| chr17 | 45411394 | 45411517 | -24.000000 | *FZD2* | 0 |
| chr17 | 29313836 | 29314019 | -23.909091 | *SARM1* | 0 |
| chr17 | 6944370 | 6944464 | -24.766667 | *SLC16A11* | 0 |
| chr18 | 23813782 | 23813925 | -41.194444 | *ANKRD29* | 0 |
| chr19 | 1036067 | 1036171 | 26.976190 | *ARHGAP45* | 0 |
| chr19* | 2471091 | 2471174 | -32.633333 | *LOC124904614* | -101 |
| chr19 | 60711170 | 60711319 | -31.533333 | *ZNF134* | 0 |
| chr20 | 61717774 | 61718057 | -22.840000 | *FAM217B* | 0 |
| chr20 | 60673451 | 60673558 | -26.176471 | *GNAS* | 0 |
| chr20 | 61717774 | 61718057 | -22.840000 | *SYCP2* | 0 |
| chr22 | 24618091 | 24618178 | -30.100000 | *CABIN1* | 0 |
| chr22* | 22931609 | 22931722 | -21.087719 | *IGL* | 0 |
| chr22 | 29141076 | 29141149 | -22.527778 | *TTC28* | 0 |
| chr22 | 42478682 | 42478801 | -21.361111 | *WBP2NL* | 0 |
| chr22 | 22931609 | 22931722 | -21.087719 | *ZNF280B* | 0 |

*removed after filter with Caco-2.

**Table S8.** *Genes not found in the Reactome Pathway Database*

| **CMF-ENZ** | **CNF-TEMPO** | **DIG CMF-ENZ** | **DIG CNF-TEMPO** |
| --- | --- | --- | --- |
| *ELF4* | *ATF7IP2* | *ACRBP* | *BTBD19* |
| *GPR137* | *EHBP1L1* | *C14orf132* | *C8orf82* |
| *GPC1-AS1* | *GPC1-AS1* | *CACNA2D4* | *DNAH11* |
|  | *IAH1* | *CATIP* | *EMX2OS* |
|  | *THNSL2* | *CCDC92B* | *GDF7* |
|  |  | *CFAP45* | *HOXA10-AS* |
|  |  | *CPAMD8* | *MINCR* |
|  |  | *DNAH11* | *REM2* |
|  |  | *ECHDC3* | *RNF32-DT* |
|  |  | *ELF4* | *SLC1A3-AS1* |
|  |  | *EMX2OS* | *TLX1NB* |
|  |  | *FLG-AS1* | *ZNF280B* |
|  |  | *GARIN5A* |  |
|  |  | *HOTAIR* |  |
|  |  | *IAH1* |  |
|  |  | *IGFBPL1* |  |
|  |  | *LEF1-AS1* |  |
|  |  | *LINC02298* |  |
|  |  | *LINC03048* |  |
|  |  | *PPM1N* |  |
|  |  | *REM2* |  |
|  |  | *RNF32-DT* |  |
|  |  | *RTN2* |  |
|  |  | *SDK1-AS1* |  |
|  |  | *SLC1A3-AS1* |  |
|  |  | *SYT13* |  |
|  |  | *TBX2-AS1* |  |
|  |  | *TFAP2E-AS1* |  |
|  |  | *TMDD1* |  |
|  |  | *TUBBP5* |  |
|  |  | *ZNF280B* |  |

**Table S9.** *Enriched pathways (p ≤ 0.05) retrieved from Reactome for CMF-ENZ, CNF-TEMPO, DIG CMF-ENZ and DIG CNF-TEMPO*

| Sample | Pathway identifier | Pathway name | p- Value | FDR | Reactions found | Genes | Found reaction identifiers |
| --- | --- | --- | --- | --- | --- | --- | --- |
| CMF-ENZ | R-HSA-3656237 | Defective EXT2 causes exostoses 2 | 0.030 | 0.535 | 4 | *GPC1* | R-HSA-9036289; R-HSA-3656254; R-HSA-9036290; R-HSA-3656267 |
| CMF-ENZ | R-HSA-3656253 | Defective EXT1 causes exostoses 1, TRPS2 and CHDS | 0.030 | 0.535 | 4 | *GPC1* | R-HSA-9036283; R-HSA-3656261; R-HSA-9036285; R-HSA-3656257 |
| CMF-ENZ | R-HSA-8980692 | RHOA GTPase cycle | 0.035 | 0.535 | 2 | *PLEKHG5*  *ARHGAP45* | R-HSA-8980691; R-HSA-8981637 |
| CMF-ENZ | R-HSA-73780 | RNA Polymerase III Chain Elongation | 0.036 | 0.535 | 3 | *POLR1C* | R-HSA-113446; R-HSA-113705; R-HSA-113451 |
| CMF-ENZ | R-HSA-4420332 | Defective B3GALT6 causes EDSP2 and SEMDJL1 | 0.0396 | 0.535 | 1 | *GPC1* | R-HSA-4420365 |
| CMF-ENZ | R-HSA-3560783 | Defective B4GALT7 causes EDS, progeroid type | 0.040 | 0.535 | 1 | *GPC1* | R-HSA-3560804 |
| CMF-ENZ | R-HSA-388479 | Vasopressin-like receptors | 0.040 | 0.5352 | 1 | *OXTR* | R-HSA-388503 |
| CMF-ENZ | R-HSA-3560801 | Defective B3GAT3 causes JDSSDHD | 0.041 | 0.5352 | 1 | *GPC1* | R-HSA-3560802 |
| CMF-ENZ | R-HSA-5656121 | Translesion synthesis by POLI | 0,041 | 0.5352 | 2 | *FAAP20* | R-HSA-5656158; R-HSA-5656105 |
| CMF-ENZ | R-HSA-5655862 | Translesion synthesis by POLK | 0.043 | 0.535 | 2 | *FAAP20* | R-HSA-5655965; R-HSA-5655835 |
| CNF-TEMPO | R-HSA-418597 | G alpha (z) signalling events | 0.001 | 0.162 | 5 | *GNAS*  *RGS20* | R-HSA-392133; R-HSA-392129; R-HSA-8982018; R-HSA-8982021; R-HSA-8981892 |
| CNF-TEMPO | R-HSA-5660489 | MTF1 activates gene expression | 0.001 | 0.175 | 2 | *CSRP1* | R-HSA-5660503; R-HSA-5660514 |
| CNF-TEMPO | R-HSA-164378 | PKA activation in glucagon signalling | 0.0011 | 0.190 | 1 | *GNAS* | R-HSA-164377 |
| CNF-TEMPO | R-HSA-163359 | Glucagon signalling in metabolic regulation | 0.003 | 0.382 | 4 | *GNAS* | R-HSA-163617; R-HSA-825631; R-HSA-164377; R-HSA-381704 |
| CNF-TEMPO | R-HSA-2022928 | HS-GAG biosynthesis | 0.004 | 0.382 | 13 | *GPC1*  *HS6ST1* | R-HSA-2076383; R-HSA-2076508; R-HSA-2076371; R-HSA-2024108; R-HSA-2022860; R-HSA-2022856; R-HSA-2076392; R-HSA-2022887; R-HSA-2022919; R-HSA-2024100; R-HSA-2076419; R-HSA-2076611; R-HSA-2022851 |
| CNF-TEMPO | R-HSA-420092 | Glucagon-type ligand receptors | 0.004 | 0.382 | 1 | *GNAS* | R-HSA-381612 |
| CNF-TEMPO | R-HSA-381676 | Glucagon-like Peptide-1 (GLP1) regulates insulin secretion | 0.006 | 0.408 | 6 | *GNAS* | R-HSA-381607; R-HSA-422320; R-HSA-381612; R-HSA-9728664; R-HSA-381704; R-HSA-381706 |
| CNF-TEMPO | R-HSA-392851 | Prostacyclin signalling through prostacyclin receptor | 0.006 | 0.408 | 3 | *GNAS* | R-HSA-392852; R-HSA-392870; R-HSA-392874 |
| CNF-TEMPO | R-HSA-9660821 | ADORA2B mediated anti-inflammatory cytokines production | 0.007 | 0.408 | 5 | *GNAS* | R-HSA-9660819; R-HSA-163617; R-HSA-392129; R-HSA-9660824; R-HSA-9660828 |
| CNF-TEMPO | R-HSA-5619072 | Defective SLC35A2 causes congenital disorder of glycosylation 2M (CDG2M) | 0.008 | 0.418 | 1 | *TIMM17B* | R-HSA-5652099 |
| CNF-TEMPO | R-HSA-432040 | Vasopressin regulates renal water homeostasis via Aquaporins | 0.008 | 0.415 | 5 | *GNAS* | R-HSA-163617; R-HSA-432195; R-HSA-422320  R-HSA-432188; R-HSA-164377 |
| CNF-TEMPO | R-HSA-5660526 | Response to metal ions | 0.009 | 0.441 | 2 | *CSRP1* | R-HSA-5660503; R-HSA-5660514 |
| CNF-TEMPO | R-HSA-445717 | Aquaporin-mediated transport | 0.012 | 0.512 | 5 | *GNAS* | R-HSA-163617; R-HSA-432195; R-HSA-422320; R-HSA-432188; R-HSA-164377 |
| CNF-TEMPO | R-HSA-418555 | G alpha (s) signalling events | 0.018 | 0.512 | 14 | *GNAS*  *PDE8B* | R-HSA-379044; R-HSA-163617  R-HSA-392129; R-HSA-9036301; R-HSA-751013; R-HSA-8964252; R-HSA-8964287; R-HSA-9036307; R-HSA-744887; R-HSA-9036308; R-HSA-744886; R-HSA-8964278; R-HSA-164381; R-HSA-418553 |
| CNF-TEMPO | R-HSA-1638091 | Heparan sulfate/heparin (HS-GAG) metabolism | 0.023 | 0.512 | 21 | *GPC1*  *HS6ST1* | R-HSA-2076383; R-HSA-1889981; R-HSA-9638064; R-HSA-2076508; R-HSA-1667005; R-HSA-1889978; R-HSA-2024084; R-HSA-2076371; R-HSA-1878002; R-HSA-2024108; R-HSA-2022860; R-HSA-2022856; R-HSA-2076392; R-HSA-2022887; R-HSA-2022919; R-HSA-2024100; R-HSA-2076419; R-HSA-2076611; R-HSA-2022851; R-HSA-1889955; R-HSA-1678694 |
| CNF-TEMPO | R-HSA-422356 | Regulation of insulin secretion | 0.023 | 0.512 | 7 | *MARCKS*  *GNAS* | R-HSA-381607; R-HSA-422320; R-HSA-381612; R-HSA-9728664; R-HSA-399978; R-HSA-381704; R-HSA-381706 |
| CNF-TEMPO | R-HSA-9634597 | GPER1 signalling | 0.029 | 0.512 | 5 | *GNAS* | R-HSA-163617; R-HSA-392129; R-HSA-9036301; R-HSA-9036307; R-HSA-9036308 |
| CNF-TEMPO | R-HSA-373080 | Class B/2 (Secretin family receptors) | 0.031 | 0.512 | 1 | *GNAS* | R-HSA-381612 |
| CNF-TEMPO | R-HSA-3656253 | Defective EXT1 causes exostoses 1, TRPS2 and CHDS | 0.032 | 0.512 | 4 | *GPC1* | R-HSA-9036283; R-HSA-3656261; R-HSA-9036285; R-HSA-3656257 |
| CNF-TEMPO | R-HSA-3656237 | Defective EXT2 causes exostoses 2 | 0.032 | 0.512 | 4 | *GPC1* | R-HSA-9036289; R-HSA-3656254; R-HSA-9036290; R-HSA-3656267 |
| CNF-TEMPO | R-HSA-73780 | RNA Polymerase III Chain Elongation | 0.037 | 0.512 | 3 | *POLR1C* | R-HSA-113446; R-HSA-113705; R-HSA-113451 |
| CNF-TEMPO | R-HSA-8980692 | RHOA GTPase cycle | 0.038 | 0.512 | 2 | *ARHGEF1*  *ARHGAP45* | R-HSA-8980691; R-HSA-8981637 |
| CNF-TEMPO | R-HSA-3560783 | Defective B4GALT7 causes EDS, progeroid type | 0.041 | 0.512 | 1 | *GPC1* | R-HSA-3560804 |
| CNF-TEMPO | R-HSA-4420332 | Defective B3GALT6 causes EDSP2 and SEMDJL1 | 0.041 | 0.512 | 1 | *GPC1* | R-HSA-4420365 |
| CNF-TEMPO | R-HSA-3560801 | Defective B3GAT3 causes JDSSDHD | 0.043 | 0.512 | 1 | *GPC1* | R-HSA-3560802 |
| CNF-TEMPO | R-HSA-9619229 | Activation of RAC1 downstream of NMDARs | 0.043 | 0.512 | 1 | *CCDC106* | R-HSA-9619376 |
| CNF-TEMPO | R-HSA-163685 | Integration of energy metabolism | 0.049 | 0.512 | 10 | *MARCKS*  *GNAS* | R-HSA-381607; R-HSA-163617; R-HSA-825631; R-HSA-422320; R-HSA-381612; R-HSA-164377; R-HSA-9728664; R-HSA-399978; R-HSA-381704; R-HSA-381706 |
| DIG CMF-ENZ | R-HSA-1963642 | PI3K events in ERBB2 signalling | 0.005 | 0.712 | 4 | *NRG1* | R-HSA-1306979; R-HSA-1250346; R-HSA-1250462; R-HSA-1250189 |
| DIG CMF-ENZ | R-HSA-1306955 | GRB7 events in ERBB2 signalling | 0.007 | 0.712 | 1 | *NRG1* | R-HSA-1306953 |
| DIG CMF-ENZ | R-HSA-2219530 | Constitutive signalling by Aberrant PI3K in Cancer | 0.012 | 0.712 | 1 | *FGF8*  *NRG1* | R-HSA-2400009 |
| DIG CMF-ENZ | R-HSA-373756 | SDK interactions | 0.014 | 0.712 | 1 | *SDK1* | R-HSA-373745 |
| DIG CMF-ENZ | R-HSA-6785631 | ERBB2 Regulates Cell Motility | 0.014 | 0.712 | 2 | *NRG1* | R-HSA-6785636; R-HSA-6785648 |
| DIG CMF-ENZ | R-HSA-8847993 | ERBB2 Activates PTK6 signalling | 0.021 | 0.712 | 2 | *NRG1* | R-HSA-8848005; R-HSA-8847995 |
| DIG CMF-ENZ | R-HSA-977444 | GABA B receptor activation | 0.0226 | 0.712 | 9 | *GABBR2*  *ADCY1* | R-HSA-170674; R-HSA-1013020; R-HSA-420688; R-HSA-170666; R-HSA-1013013; R-HSA-392206; R-HSA-1013012; R-HSA-170686; R-HSA-170671 |
| DIG CMF-ENZ | R-HSA-991365 | Activation of GABAB receptors | 0.028 | 0.712 | 8 | *GABBR2*  *ADCY1* | R-HSA-17067; R-HSA-1013020; R-HSA-170666; R-HSA-1013013; R-HSA-392206; R-HSA-1013012; R-HSA-170686; R-HSA-170671 |
| DIG CMF-ENZ | R-HSA-4085011 | Defective GNE causes sialuria, NK and IBM2 | 0.028 | 0.712 | 2 | *GNE* | R-HSA-4088322; R-HSA-4088338 |
| DIG CMF-ENZ | R-HSA-5602415 | UNC93B1 deficiency - HSE | 0.028 | 0.712 | 1 |  | R-HSA-5607838 |
| DIG CMF-ENZ | R-HSA-9013406 | RHOQ GTPase cycle | 0.035 | 0.712 | 2 | *OBSCN*  *STOM* | R-HSA-9018747; R-HSA-9018746 |
| DIG CMF-ENZ | R-HSA-1358803 | Downregulation of ERBB2:ERBB3 signalling | 0.035 | 0.712 | 2 | *NRG1* | R-HSA-1358798; R-HSA-1358792 |
| DIG CMF-ENZ | R-HSA-8980692 | RHOA GTPase cycle | 0.037 | 0.712 | 2 | *OBSCN*  *STOM*  *ARHGEF1* | R-HSA-9013009; R-HSA-8980691 |
| DIG CMF-ENZ | R-HSA-977443 | GABA receptor activation | 0.042 | 0.712 | 9 | *GABBR2*  *ADCY1* | R-HSA-170674; R-HSA-1013020; R-HSA-420688; R-HSA-170666; R-HSA-1013013; R-HSA-392206; R-HSA-1013012; R-HSA-170686; R-HSA-170671 |
| DIG CMF-ENZ | R-HSA-1483101 | Synthesis of PS | 0.045 | 0.712 | 2 | *STOM* | R-HSA-1483186; R-HSA-1483089 |
| DIG CMF-ENZ | R-HSA-5576890 | Phase 3 - rapid repolarisation | 0.045 | 0.712 | 3 | *KCNE3* | R-HSA-5577237; R-HSA-9613264; R-HSA-5577050 |
| DIG CMF-ENZ | R-HSA-9013026 | RHOB GTPase cycle | 0.047 | 0.712 | 2 | *STOM*  *ARHGEF1* | R-HSA-9013023; R-HSA-9013024 |
| DIG CNF-TEMPO | R-HSA-164378 | PKA activation in glucagon signalling | 0.002 | 0.319 | 1 | *GNAS* | R-HSA-164377 |
| DIG CNF-TEMPO | R-HSA-2243919 | Crosslinking of collagen fibrils | 0.002 | 0.319 | 3 | *LOXL2* | R-HSA-2022141; R-HSA-2395340; R-HSA-2002466 |
| DIG CNF-TEMPO | R-HSA-1306955 | GRB7 events in ERBB2 signalling | 0.002 | 0.319 | 1 | *NRG1* | R-HSA-1306953 |
| DIG CNF-TEMPO | R-HSA-2219530 | Constitutive signalling by aberrant PI3K in Cancer | 0.003 | 0.319 | 2 | *NRG1*  *PIK3R1* | R-HSA-2394007; R-HSA-2400009 |
| DIG CNF-TEMPO | R-HSA-9664565 | Signalling by ERBB2 KD Mutants | 0.004 | 0.35 | 10 | *NRG1*  *PIK3R1* | R-HSA-9664976; R-HSA-9664983; R-HSA-9664567; R-HSA-9664646; R-HSA-9664950; R-HSA-9664933; R-HSA-9664664; R-HSA-9664991; R-HSA-9664940; R-HSA-9664588 |
| DIG CNF-TEMPO | R-HSA-1227990 | Signalling by ERBB2 in Cancer | 0.004 | 0.369 | 20 | *NRG1*  *PIK3R1* | R-HSA-9664976; R-HSA-9634391; R-HSA-9664983; R-HSA-9664567; R-HSA-9664950; R-HSA-9664664; R-HSA-9665407; R-HSA-9664991; R-HSA-9665699; R-HSA-9665697; R-HSA-9665415; R-HSA-9664646; R-HSA-9664933; R-HSA-9665701; R-HSA-9665700; R-HSA-9665705; R-HSA-9665416; R-HSA-9665709; R-HSA-9664940; R-HSA-9664588 |
| DIG CNF-TEMPO | R-HSA-6785631 | ERBB2 Regulates Cell Motility | 0.005 | 0.374 | 2 | *NRG1* | R-HSA-6785636; R-HSA-6785648 |
| DIG CNF-TEMPO | R-HSA-163359 | Glucagon signalling in metabolic regulation | 0.006 | 0.374 | 4 | *GNAS* | R-HSA-163617; R-HSA-825631; R-HSA-164377; R-HSA-381704 |
| DIG CNF-TEMPO | R-HSA-373080 | Class B/2 (Secretin family receptors) | 0.006 | 0.374 | 2 | *FZD2*  *GNAS* | R-HSA-381612; R-HSA-201708 |
| DIG CNF-TEMPO | R-HSA-8847993 | ERBB2 Activates PTK6 signalling | 0.007 | 0.374 | 2 | *NRG1* | R-HSA-8848005; R-HSA-8847995 |
| DIG CNF-TEMPO | R-HSA-420092 | Glucagon-type ligand receptors | 0.007 | 0.374 | 1 | *GNAS* | R-HSA-381612 |
| DIG CNF-TEMPO | R-HSA-8980692 | RHOA GTPase cycle | 0.008 | 0.407 | 2 | *PKN3*  *PIK3R1*  *ARHGAP45* | R-HSA-9013009; R-HSA-8981637 |
| DIG CNF-TEMPO | R-HSA-381676 | Glucagon-like Peptide-1 (GLP1) regulates insulin secretion | 0.0104 | 0.452 | 6 | *GNAS* | R-HSA-381607; R-HSA-422320; R-HSA-381612; R-HSA-9728664; R-HSA-381704; R-HSA-381706 |
| DIG CNF-TEMPO | R-HSA-392851 | Prostacyclin signalling through prostacyclin receptor | 0.011 | 0.452 | 5 | *GNAS*  *FZD2* | R-HSA-9726949; R-HSA-392852; R-HSA-391942; R-HSA-392870; R-HSA-392874 |
| DIG CNF-TEMPO | R-HSA-9660821 | ADORA2B mediated anti-inflammatory cytokines production | 0.011 | 0.452 | 5 | *GNAS* | R-HSA-9660819; R-HSA-163617; R-HSA-392129; R-HSA-9660824; R-HSA-9660828 |
| DIG CNF-TEMPO | R-HSA-1358803 | Downregulation of ERBB2:ERBB3 signalling | 0.013 | 0.452 | 2 | *NRG1* | R-HSA-1358798; R-HSA-1358792 |
| DIG CNF-TEMPO | R-HSA-418597 | G alpha (z) signalling events | 0.013 | 0,452 | 1 | *GNAS* | R-HSA-392129 |
| DIG CNF-TEMPO | R-HSA-4085011 | Defective GNE causes sialuria, NK and IBM2 | 0.013 | 0.465 | 2 | *GNE* | R-HSA-4088322; R-HSA-4088338 |
| DIG CNF-TEMPO | R-HSA-432040 | Vasopressin regulates renal water homeostasis via Aquaporins | 0.0141 | 0.467 | 5 | *GNAS* | R-HSA-163617; R-HSA-432195; R-HSA-422320; R-HSA-432188; R-HSA-164377 |
| DIG CNF-TEMPO | R-HSA-1566948 | Elastic fibre formation | 0.015 | 0.478 | 1 | *LOXL2* | R-HSA-2129375 |
| DIG CNF-TEMPO | R-HSA-5602415 | UNC93B1 deficiency - HSE | 0.016 | 0.478 | 1 | *PIK3R1*  *TRIL* | R-HSA-5607838 |
| DIG CNF-TEMPO | R-HSA-9013026 | RHOB GTPase cycle | 0.0172 | 0.492 | 2 | *PKN3*  *PIK3R1* | R-HSA-9013022; R-HSA-9013024 |
| DIG CNF-TEMPO | R-HSA-1250196 | SHC1 events in ERBB2 signalling | 0.0192 | 0.492 | 4 | *NRG1*  *PIK3R1* | R-HSA-1250195; R-HSA-1250486; R-HSA-1963578; R-HSA-1250463 |
| DIG CNF-TEMPO | R-HSA-1474290 | Collagen formation | 0.0202 | 0.492 | 24 | *COL23A1*  *LOXL2* | R-HSA-8948232; R-HSA-1650808: R-HSA-2002460; R-HSA-8948234; R-HSA-2022141; R-HSA-2022073; R-HSA-8948224; R-HSA-2395340; R-HSA-8948226; R-HSA-8948228; R-HSA-2089971; R-HSA-8948230; R-HSA-1981104; R-HSA-8944230; R-HSA-8948231; R-HSA-8948219; R-HSA-8944220; R-HSA-2152276; R-HSA-1981128; R-HSA-8948222; R-HSA-1980233; R-HSA-1981157; R-HSA-2002466; R-HSA-1981120 |
| DIG CNF-TEMPO | R-HSA-6811558 | PI5P, PP2A and IER3 Regulate PI3K/AKT signalling | 0.020 | 0.492 | 1 | *NRG1*  *PIK3R1* | R-HSA-2316434 |
| DIG CNF-TEMPO | R-HSA-2022090 | Assembly of collagen fibrils and other multimeric structures | 0.021 | 0.492 | 3 | *LOXL2* | R-HSA-2022141; R-HSA-2395340; R-HSA-2002466 |
| DIG CNF-TEMPO | R-HSA-445717 | Aquaporin-mediated transport | 0.021 | 0.492 | 5 | *GNAS* | R-HSA-163617; R-HSA-432195; R-HSA-422320; R-HSA-432188; R-HSA-164377 |
| DIG CNF-TEMPO | R-HSA-9013106 | RHOC GTPase cycle | 0.023 | 0.492 | 2 | *PKN3*  *PIK3R1* | R-HSA-9013111; R-HSA-9013110 |
| DIG CNF-TEMPO | R-HSA-199418 | Negative regulation of the PI3K/AKT network | 0.028 | 0.492 | 1 | *NRG1*  *PIK3R1* | R-HSA-2316434 |
| DIG CNF-TEMPO | R-HSA-9028335 | Activated NTRK2 signals through PI3K | 0.029 | 0.492 | 2 | *PIK3R1* | R-HSA-9028505; R-HSA-9028519 |
| DIG CNF-TEMPO | R-HSA-9665686 | Signalling by ERBB2 TMD/JMD mutants | 0.033 | 0.492 | 6 | *NRG1*  *PIK3R1* | R-HSA-9665699; R-HSA-9665697; R-HSA-9665701; R-HSA-9665700; R-HSA-9665705; R-HSA-9665709 |
| DIG CNF-TEMPO | R-HSA-5140745 | WNT5A-dependent internalization of FZD2, FZD5 and ROR2 | 0.038 | 0.492 | 2 | *FZD2* | R-HSA-5140747; R-HSA-5140741 |
| DIG CNF-TEMPO | R-HSA-9842640 | signalling by LTK in cancer | 0.039 | 0.492 | 1 | *PIK3R1* | R-HSA-9845032 |
| DIG CNF-TEMPO | R-HSA-5603037 | IRAK4 deficiency (TLR5) | 0.0419 | 0.492 | 1 | *ZNF597* | R-HSA-5602472 |
| DIG CNF-TEMPO | R-HSA-187042 | TRKA activation by NGF | 0.0419 | 0.492 | 2 | *PIK3R1* | R-HSA-166538; R-HSA-166542 |
| DIG CNF-TEMPO | R-HSA-422356 | Regulation of insulin secretion | 0.049 | 0.492 | 7 | *MARCKS*  *GNAS* | R-HSA-381607; R-HSA-422320; R-HSA-381612; R-HSA-9728664; R-HSA-399978; R-HSA-381704; R-HSA-381706 |
| DIG CNF-TEMPO | R-HSA-9634597 | GPER1 signalling | 0.049 | 0.492 | 6 | *GNAS*  *PIK3R1* | R-HSA-163617; R-HSA-392129; R-HSA-9036301; R-HSA-9036307; R-HSA-9624014; R-HSA-9036308 |
| DIG CNF-TEMPO | R-HSA-9673767 | Signalling by PDGFRA transmembrane, juxtamembrane and kinase domain mutants | 0.0496 | 0.492 | 2 | *PIK3R1* | R-HSA-9672177; R-HSA-9672172 |
| DIG CNF-TEMPO | R-HSA-9673770 | Signalling by PDGFRA extracellular domain mutants | 0.049 | 0.492 | 2 | *PIK3R1* | R-HSA-9672162; R-HSA-9672178 |
| DIG CNF-TEMPO | R-HSA-1963642 | PI3K events in ERBB2 signalling | 0.001 | 0.023 | 6 | *NRG1*  *PIK3R1* | R-HSA-1306979; R-HSA-1306965; R-HSA-1250346; R-HSA-1250462; R-HSA-1306957; R-HSA-1250189 |
| DIG CNF-TEMPO | R-HSA-1250342 | PI3K events in ERBB4 signalling | 0.001 | 0.285 | 2 | *NRG1*  *PIK3R1* | R-HSA-1250370; R-HSA-1250353 |

FDR- False Discovery rate

**Table S10.** *Enriched terms (p ≤ 0.05) retrieved from GO Biological Process for the two undigested CNMs*

| Go Term ID | Term | CMF-ENZ | | | CNF -TEMPO | | |
| --- | --- | --- | --- | --- | --- | --- | --- |
|  |  | ***p*-value** | **FE** | **Genes** | ***p*-value** | **FE** | **genes** |
| 0006351 | DNA-templated transcription | 0.045 | 4.69 | *PLAC8, HOXA9, POLR1C, GRHL3* | *-* | *-* | *-* |
| 0007275 | multicellular organism development | 0.009 | 2.11 | *NDRG4, OXTR, KDM2B, SYCP2, AATK, ZBTB45, GRHL3, SOX12, NID1, STK3, HOXA9, GPC1, OBSL1* | 0.015 | 1.97 | *KDM2B, CHD7, ZBTB45, NID1, HS6ST1, HHEX, MARCKS, GPC1, S100A6, GNAS, SARM1, ADAM8, OBSL1* |
| 0007389 | pattern specification process | 0.031 | 5.62 | *NDRG4, HOXA9, KDM2B, GRHL3* | *-* | *-* | *-* |
| 0007399 | nervous system development | 0.039 | 2.36 | *OXTR, KDM2B, GPC1, AATK, GRHL3, ZBTB45, SOX12, STK3* | *-* | *-* | *-* |
| 0007417 | central nervous system development | 0.015 | 3.90 | *OXTR, KDM2B, AATK, GRHL3, SOX12, STK3* | *-* | *-* | *-* |
| 0009653 | anatomical structure morphogenesis | 0.014 | 2.59 | *NDRG4, HOXA9, SYCP2, KDM2B, GPC1, GRHL3, SOX12, OBSL1, STK3* | 0.022 | 2.42 | *KDM2B, CSRP1, GPC1, CHD7, S100A6, GNAS, ADAM8, HS6ST1, OBSL1* |
| 0010647 | positive regulation of cell communication | 0.049 | 2.48 | *NDRG4, OXTR, BAD, PLEKHG5, GPR137, NID1, STK3* | *-* | *-* | *-* |
| 0021915 | neural tube development | 0.022 | 12.56 | *KDM2B, GRHL3, STK3* | *-* | *-* | *-* |
| 0023056 | positive regulation of signalling | 0.049 | 2.47 | *NDRG4, OXTR, BAD, PLEKHG5, GPR137, NID1, STK3* | *-* | *-* | *-* |
| 0032502 | developmental process | 0.025 | 1.69 | *NDRG4, OXTR, KDM2B, SYCP2, AATK, ZBTB45, GRHL3, SOX12, NID1, STK3, PLAC8, HOXA9, ELF4, GPC1, OBSL1* | *-* | *-* | *-* |
| 0035295 | tube development | 0.042 | 3.62 | *NDRG4, OXTR, KDM2B, GRHL3, STK3* | *-* | *-* | *-* |
| 0045595 | regulation of cell differentiation | 0.008 | 3.18 | *HOXA9, BAD, GPC1, GPR137, SOX12, NID1, OBSL1, STK3* | *-* | *-* | *-* |
| 0045597 | positive regulation of cell differentiation | 0.010 | 4.32 | *BAD, GPC1, SOX12, NID1, OBSL1, STK3* | *-* | *-* | *-* |
| 0060284 | regulation of cell development | 0.036 | 3.81 | *HOXA9, BAD, GPR137, SOX12, OBSL1* | *-* | *-* | *-* |
| 0048513 | animal organ development | 0.007 | 2.46 | *NDRG4, HOXA9, OXTR, SYCP2, KDM2B, AATK, GRHL3, SOX12, NID1, OBSL1, STK3* | *-* | *-* | *-* |
| 0048518 | positive regulation of biological process | 0.025 | 1.63 | *NDRG4, OXTR, KDM2B, BAD, PLEKHG5, GRHL3, SOX12, NID1, STK3, PLAC8, EHD2, HOXA9, ELF4, GPC1, GPR137, OBSL1* | *-* | *-* | *-* |
| 0048522 | positive regulation of cellular process | 0.010 | 1.81 | *NDRG4, OXTR, KDM2B, BAD, PLEKHG5, GRHL3, SOX12, NID1, STK3, PLAC8, EHD2, HOXA9, ELF4, GPC1, GPR137, OBSL1* | *-* | *-* | *-* |
| 0048562 | embryonic organ morphogenesis | 0.010 | 8.62 | *NDRG4, HOXA9, KDM2B, GRHL3* | *-* | *-* | *-* |
| 0048568 | embryonic organ development | 0.004 | 7.08 | *NDRG4, HOXA9, KDM2B, GRHL3, STK3* | *-* | *-* | *-* |
| 0048598 | embryonic morphogenesis | 0.010 | 5.56 | *NDRG4, HOXA9, KDM2B, GRHL3, STK3* | *-* | *-* | *-* |
| 0048731 | system development | 0.003 | 2.35 | *NDRG4, OXTR, KDM2B, SYCP2, AATK, ZBTB45, GRHL3, SOX12, NID1, STK3, HOXA9, GPC1, OBSL1* | 0.018 | 2.03 | *MARCKS, KDM2B, GPC1, CHD7, S100A6, GNAS, SARM1, ADAM8, ZBTB45, NID1, HS6ST1, OBSL1* |
| 0051128 | regulation of cellular component organization | 0.024 | 2.36 | *EHD2, NDRG4, OXTR, KDM2B, BAD, LRFN1, GRHL3, NID1, OBSL1* | *-* | *-* | *-* |
| 0051239 | regulation of multicellular organismal process | 0.011 | 2.29 | *PLAC8, HOXA9, OXTR, KDM2B, ELF4, BAD, GPR137, ZBTB45, SOX12, OBSL1, STK3* | *-* | *-* | *-* |
| 0060562 | epithelial tube morphogenesis | 0.011 | 8.25 | *NDRG4, KDM2B, GRHL3, STK3* | *-* | *-* | *-* |
| 0001838 | embryonic epithelial tube formation | 0.014 | 16.19 | *KDM2B, GRHL3, STK3* | *-* | *-* | *-* |
| 0001841 | neural tube formation | 0.010 | 19.45 | *KDM2B, GRHL3, STK3* | *-* | *-* | *-* |
| 0002009 | morphogenesis of an epithelium | 0.030 | 5.64 | *NDRG4, KDM2B, GRHL3, STK3* | *-* | *-* | *-* |
| 0016331 | morphogenesis of embryonic epithelium | 0.018 | 13.67 | *KDM2B, GRHL3, STK3* | *-* | *-* | *-* |
| 0035148 | tube formation | 0.019 | 13.67 | *KDM2B, GRHL3, STK3* | *-* | *-* | *-* |
| 0072175 | epithelial tube formation | 0.016 | 15.21 | *KDM2B, GRHL3, STK3* | *-* | *-* | *-* |
| 1903706 | regulation of hemopoiesis | 0.025 | 6.12 | *HOXA9, BAD, GPR137, SOX12* | *-* | *-* | *-* |
| 2000241 | regulation of reproductive process | 0.039 | 9.15 | *OXTR, SYCP2, STK3* | *-* | *-* | *-* |
| 2001135 | regulation of endocytic recycling | 0.03 | 58.63 | *EHD2, NDRG4* | *-* | *-* | *-* |
| 0009966 | regulation of signal transduction | *-* | *-* | *-* | 0.024 | 2.10 | *LRRC14, HHEX, GPC1, GNAS, SARM1, RGS20, ARHGEF1, ADAM8, NID1, PDE8B, ARHGAP45* |
| 0010646 | regulation of cell communication | - | *-* | *-* | 0.019 | 2.02 | *LRRC14, HHEX, GPC1, CHD7, GNAS, SARM1, RGS20, ARHGEF1, ADAM8, NID1, PDE8B, ARHGAP45* |
| 0010927 | cellular component assembly involved in morphogenesis | *-* | *-* | *-* | 0.017 | 14.67 | *CSRP1, GPC1, OBSL1* |
| 0023051 | regulation of signalling | *-* | *-* | *-* | 0.019 | 2.10 | *LRRC14, HHEX, GPC1, CHD7, GNAS, SARM1, RGS20, ARHGEF1, ADAM8, NID1, PDE8B, ARHGAP45* |
| 0032501 | multicellular organismal process | *-* | *-* | *-* | 0.043 | 1.55 | *KDM2B, CHD7, ZBTB45, NID1, HS6ST1, FABP1, HHEX, MARCKS, CSRP1, GPC1, S100A6, GNAS, SARM1, ADAM8, PDE8B, OBSL1* |
| 0032989 | cellular anatomical entity morphogenesis | *-* | *-* | *-* | 0.017 | 14.67 | *CSRP1, GPC1, OBSL1* |
| 0034122 | negative regulation of toll-like receptor signalling pathway | *-* | *-* | *-* | 0.033 | 57.34 | *LRRC14, SARM1* |
| 0048646 | anatomical structure formation involved in morphogenesis | *-* | *-* | *-* | 0.006 | 4.43 | *KDM2B, CSRP1, GPC1, GNAS, ADAM8, HS6ST1, OBSL1* |
| 0050793 | regulation of developmental process | *-* | *-* | *-* | 0.041 | 2.15 | *HHEX, KDM2B, GPC1, CHD7, GNAS, SARM1, ADAM8, NID1, OBSL1* |

FE – Fold enrichment

**Table S11.** *Enriched terms (p ≤ 0.05) retrieved from GO Biological Process for the two digested CNMs*

| Go Term ID | Term | DIG CMF-ENZ | | | DIG CNF -TEMPO | | |
| --- | --- | --- | --- | --- | --- | --- | --- |
|  |  | ***p*-value** | **FE** | **Genes** | ***p*-value** | **FE** | **genes** |
| 0000902 | cell morphogenesis | 0.016 | 3.01 | *SDK1, FGF8, NFIA, BCL6, S100A6, CNTN4, ADCY1, VAX2* | *-* | *-* | *-* |
| 0001501 | skeletal system development | 0.009 | 3.82 | *HOXA9, BMP2, FGF8, NFIA, PTH1R, HOXC11, BMPR1A* | *-* | *-* | *-* |
| 0001654 | eye development | 0.014 | 4.25 | *SDK1, NFIA, KDM2B, CPAMD8, VAX2, SCAPER* | *-* | *-* | *-* |
| 0043010 | camera-type eye development | 0.034 | 4.01 | *SDK1, NFIA, KDM2B, VAX2, SCAPER* | *-* | *-* | *-* |
| 0060041 | retina development in camera-type eye | 0.020 | 6.87 | *SDK1, NFIA, VAX2, SCAPER* | *-* | *-* | *-* |
| 0090596 | sensory organ morphogenesis | 0.019 | 4.87 | *SDK1, FZD2, FGF8, KDM2B, VAX2* | *-* | *-* | *-* |
| 0150063 | visual system development | 0.013 | 4.21 | *SDK1, NFIA, KDM2B, CPAMD8, VAX2, SCAPER* | *-* | *-* | *-* |
| 0003156 | regulation of animal organ formation | 0.006 | 29.32 | *BMP2, FGF8, HOXC11* | *-* | *-* | *-* |
| 0001759 | organ induction | 0.004 | 38.64 | *BMP2, FGF8, HOXC11* | *-* | *-* | *-* |
| 0042487 | regulation of odontogenesis of dentin-containing tooth | 0.041 | 47.23 | *BMP2, FGF8* | *-* | *-* | *-* |
| 0110110 | positive regulation of animal organ morphogenesis | 0.007 | 23.62 | *BMP2, FGF8, HOXC11* | *-* | *-* | *-* |
| 0003197 | endocardial cushion development | 0.016 | 16.67 | *BMP2, FGF8, BMPR1A* | *-* | *-* | *-* |
| 0048762 | mesenchymal cell differentiation | 0.023 | 6.44 | *BMP2, FGF8, NRG1, BMPR1A* | *-* | *-* | *-* |
| 0072132 | mesenchyme morphogenesis | 0.016 | 15.18 | *BMP2, FGF8, BMPR1A* | *-* | *-* | *-* |
| 0003272 | endocardial cushion formation | 0.003 | 34.01 | *BMP2, FGF8, BMPR1A* | *-* | *-* | *-* |
| 0003203 | endocardial cushion morphogenesis | 0.009 | 21.25 | *BMP2, FGF8, BMPR1A* | *-* | *-* | *-* |
| 0006357 | regulation of transcription by RNA polymerase II | 0.020 | 1.80 | *MESP2, XRCC6, TFAP2E, KDM2B, OTP, VAX2, HOXC11, ZNF75A, HOXA9, BMP2, NFIA, BCL6, ELF4, ZNF280B, ZNF664, BMPR1A, ZNF662* | *-* | *-* | *-* |
| 0006355 | regulation of DNA-templated transcription | 0.032 | 1.59 | *MESP2, XRCC6, FZD2, TFAP2E, KDM2B, NRG1, OTP, ADCY1, VAX2, HOXC11, ZNF75A, HOXA9, BMP2, NFIA, BCL6, ELF4, ZNF280B, ZNF664, BMPR1A, ZNF662* | *-* | *-* | *-* |
| 2001141 | regulation of RNA biosynthetic process | 0.034 | 1.58 | *MESP2, XRCC6, FZD2, TFAP2E, KDM2B, NRG1, OTP, ADCY1, VAX2, HOXC11, ZNF75A, HOXA9, BMP2, NFIA, BCL6, ELF4, ZNF280B, ZNF664, BMPR1A, ZNF662* | *-* | *-* | *-* |
| 0007154 | cell communication | 0.035 | 1.42 | *MESP2, SHC3, TRIL, DIXDC1, ADCY1, PTH1R, NID1, LTB4R, FGF8, TMDD1, GABBR2, FZD2, BNIP3, NRG1, SYT13, DGKZ, VAX2, RAB33A, CHFR, BMP2, BCL6, NFIA, S100A6, SARM1, ARHGEF1, BMPR1A, LY6E* | *-* | *-* | *-* |
| 0007165 | signal transduction | 0.028 | 1.47 | *MESP2, SHC3, TRIL, DIXDC1, ADCY1, PTH1R, NID1, LTB4R, FGF8, TMDD1, GABBR2, FZD2, BNIP3, NRG1, DGKZ, VAX2, RAB33A, CHFR, BMP2, BCL6, NFIA, S100A6, SARM1, ARHGEF1, BMPR1A, LY6E* | *-* | *-* | *-* |
| 0007166 | cell surface receptor signalling pathway | 0.035 | 1.82 | *MESP2, FZD2, SHC3, TRIL, DIXDC1, NRG1, PTH1R, NID1, VAX2, BMP2, FGF8, NFIA, BMPR1A, LY6E* | *-* | *-* | *-* |
| 0007409 | axonogenesis | 0.015 | 4.08 | *SDK1, FGF8, S100A6, CNTN4, ADCY1, VAX2* | *-* | *-* | *-* |
| 0030182 | neuron differentiation | 0.002 | 2.95 | *SDK1, BMP2, FZD2, FGF8, NFIA, BCL6, S100A6, OTP, CNTN4, ADCY1, VAX2, BMPR1A* | *-* | *-* | *-* |
| 0048667 | cell morphogenesis involved in neuron differentiation | 0.030 | 3.39 | *SDK1, FGF8, S100A6, CNTN4, ADCY1, VAX2* | *-* | *-* | *-* |
| 0048699 | generation of neurons | 0.003 | 2.76 | *SDK1, BMP2, FZD2, FGF8, NFIA, BCL6, S100A6, OTP, CNTN4, ADCY1, VAX2, BMPR1A* | *-* | *-* | *-* |
| 0048812 | neuron projection morphogenesis | 0.040 | 3.14 | *SDK1, FGF8, S100A6, CNTN4, ADCY1, VAX2* | *-* | *-* | *-* |
| 0048858 | cell projection morphogenesis | 0.043 | 3.08 | *SDK1, FGF8, S100A6, CNTN4, ADCY1, VAX2* | *-* | *-* | *-* |
| 0061564 | axon development | 0.024 | 3.60 | *SDK1, FGF8, S100A6, CNTN4, ADCY1, VAX2* | *-* | *-* | *-* |
| 0120039 | plasma membrane bounded cell projection morphogenesis | 0.0416 | 3.11 | *SDK1, FGF8, S100A6, CNTN4, ADCY1, VAX2* | *-* | *-* | *-* |
| 0007417 | central nervous system development | 0.003 | 3.00 | *SDK1, BMP2, FGF8, SHC3, KDM2B, BNIP3, DIXDC1, OTP, CNTN4, VAX2, BMPR1A* | *-* | *-* | *-* |
| 0007420 | brain development | 0.001 | 3.71 | *SDK1, BMP2, FGF8, KDM2B, BNIP3, DIXDC1, OTP, CNTN4, VAX2, BMPR1A* | *-* | *-* | *-* |
| 0021537 | telencephalon development | 0.017 | 5.06 | *BMP2, FGF8, KDM2B, BNIP3, DIXDC1* | *-* | *-* | *-* |
| 0007423 | sensory organ development | 0.001 | 4.16 | *SDK1, BMP2, FZD2, FGF8, NFIA, KDM2B, CPAMD8, VAX2, SCAPER* | *-* | *-* | *-* |
| 0007492 | endoderm development | 0.035 | 10.00 | *FGF8, HOXC11, BMPR1A* | *-* | *-* | *-* |
| 0007507 | heart development | 0.017 | 3.33 | *MESP2, BMP2, DNAH11, FZD2, FGF8, NRG1, BMPR1A* | *-* | *-* | *-* |
| 0007548 | sex differentiation | 0.029 | 4.23 | *HOXA9, FGF8, SYCP2, SCAPER, BMPR1A* | *-* | *-* | *-* |
| 0008283 | cell population proliferation | 0.001 | 3.78 | *BMP2, FGF8, NFIA, BCL6, ELF4, DIXDC1, OTP, NRG1, PTH1R, BMPR1A* | *-* | *-* | *-* |
| 0008284 | positive regulation of cell population proliferation | 0.026 | 2.484 | *BMP2, FGF8, BCL6, S100A6, OTP, NRG1, EMC10, PTH1R, BMPR1A* | *-* | *-* | *-* |
| 0009790 | embryo development | 0.013 | 2.60 | *MESP2, HOXA9, BMP2, FZD2, FGF8, KDM2B, PTH1R, HOXC11, VAX2, BMPR1A* | *-* | *-* | *-* |
| 0009798 | axis specification | 0.050 | 8.25 | *MESP2, VAX2, BMPR1A* | *-* | *-* | *-* |
| 0009950 | dorsal/ventral axis specification | 0.048 | 40.48 | *VAX2, BMPR1A* | *-* | *-* | *-* |
| 0009953 | dorsal/ventral pattern formation | 0.038 | 9.66 | *FGF8, VAX2, BMPR1A* | *-* | *-* | *-* |
| 0021536 | diencephalon development | 0.003 | 13.66 | *BMP2, FGF8, OTP, BMPR1A* | *-* | *-* | *-* |
| 0022008 | neurogenesis | 0.003 | 2.59 | *FZD2, BNIP3, OTP, ADCY1, VAX2, SDK1, BMP2, FGF8, NFIA, BCL6, S100A6, CNTN4, BMPR1A* | *-* | *-* | *-* |
| 0022414 | reproductive process | 0.021 | 2.13 | *HOXA9, DNAH11, FGF8, SYCP2, KDM2B, BCL6, TEX15, ACRBP, SCAPER, CFAP45, CHFR, BMPR1A* | 0.049 | 2.52 | *ZDBF2, DNAH11, SYCP2, SOHLH1, WBP2NL, GNAS, GDF7* |
| 0022603 | regulation of anatomical structure morphogenesis | 0.016 | 2.73 | *BMP2, FZD2, FGF8, BCL6, BNIP3, SARM1, EMC10, HOXC11, BMPR1A* | *-* | *-* | *-* |
| 0023052 | signaling | 0.032 | 1.44 | *MESP2, SHC3, TRIL, DIXDC1, ADCY1, PTH1R, NID1, LTB4R, FGF8, TMDD1, GABBR2, FZD2, BNIP3, NRG1, SYT13, DGKZ, VAX2, RAB33A, CHFR, BMP2, BCL6, NFIA, S100A6, SARM1, ARHGEF1, BMPR1A, LY6E* | *-* | *-* | *-* |
| 0030154 | cell differentiation | 0.018 | 1.70 | *FZD2, SYCP2, BNIP3, NRG1, OTP, ADCY1, PTH1R, TEX15, ACRBP, VAX2, SDK1, HOXA9, BMP2, OBSCN, FGF8, NFIA, BCL6, ELF4, S100A6, SARM1, CNTN4, BMPR1A* | *-* | *-* | *-* |
| 0030509 | BMP signalling pathway | 0.036 | 9.89 | *BMP2, NFIA, BMPR1A* | *-* | *-* | *-* |
| 0071773 | cellular response to BMP stimulus | 0.047 | 8.50 | *BMP2, NFIA, BMPR1A* | *-* | *-* | *-* |
| 0030917 | midbrain-hindbrain boundary development | 0.024 | 80.97 | *FGF8, KDM2B* | *-* | *-* | *-* |
| 0021903 | rostrocaudal neural tube patterning | 0.038 | 51.53 | *FGF8, KDM2B* | *-* | *-* | *-* |
| 0031128 | developmental induction | 0.006 | 25.76 | *BMP2, FGF8, HOXC11* | *-* | *-* | *-* |
| 0032501 | multicellular organismal process | 0.003 | 1.52 | *MESP2, SHC3, SYCP2, KCNE3, DIXDC1, EMC10, OTP, ADCY1, PTH1R, NID1, HOXC11, LTB4R, HOXA9, FGF8, CPAMD8, ST3GAL4, SCAPER, CFAP45, DNAH11, FZD2, KDM2B, BNIP3, NRG1, CACNA2D4, DGKZ, VAX2, SDK1, BMP2, BCL6, NFIA, ELF4, S100A6, SARM1, CNTN4, BMPR1A* | *-* | *-* | *-* |
| 0032502 | developmental process | 0.001 | 1.61 | *MESP2, SHC3, SYCP2, DIXDC1, EMC10, OTP, ADCY1, PTH1R, NID1, HOXC11, ACRBP, HOXA9, FGF8, CPAMD8, SCAPER, CFAP45, DNAH11, FZD2, KDM2B, TFAP2E, BNIP3, NRG1, TEX15, VAX2, SDK1, BMP2, OBSCN, BCL6, NFIA, ELF4, S100A6, SARM1, CNTN4, BMPR1A* | *-* | *-* | *-* |
| 0035108 | limb morphogenesis | 0.002 | 8.97 | *HOXA9, FGF8, NFIA, HOXC11, BMPR1A* | *-* | *-* | *-* |
| 0030326 | embryonic limb morphogenesis | 0.010 | 8.87 | *HOXA9, FGF8, HOXC11, BMPR1A* | *-* | *-* | *-* |
| 0035107 | appendage morphogenesis | 0.002 | 8.97 | *HOXA9, FGF8, NFIA, HOXC11, BMPR1A* | *-* | *-* | *-* |
| 0035113 | embryonic appendage morphogenesis | 0.010 | 8.86 | *HOXA9, FGF8, HOXC11, BMPR1A* | *-* | *-* | *-* |
| 0060173 | limb development | 0.005 | 7.27 | *HOXA9, FGF8, NFIA, HOXC11, BMPR1A* | *-* | *-* | *-* |
| 0035270 | endocrine system development | 0.011 | 8.46 | *BMP2, FGF8, OTP, BMPR1A* | *-* | *-* | *-* |
| 0035295 | tube development | 0.043 | 2.43 | *BMP2, FZD2, FGF8, NFIA, KDM2B, EMC10, SCAPER, BMPR1A* | *-* | *-* | *-* |
| 0043009 | chordate embryonic development | 0.002 | 3.81 | *MESP2, HOXA9, BMP2, FZD2, FGF8, KDM2B, PTH1R, HOXC11, BMPR1A* | *-* | *-* | *-* |
| 0009792 | embryo development ending in birth or egg hatching | 0.003 | 3.70 | *MESP2, HOXA9, BMP2, FZD2, FGF8, KDM2B, PTH1R, HOXC11, BMPR1A* | *-* | *-* | *-* |
| 0048562 | embryonic organ morphogenesis | 0.005 | 5.43 | *HOXA9, FZD2, FGF8, KDM2B, HOXC11, VAX2* | *-* | *-* | *-* |
| 0048568 | embryonic organ development | 0.006 | 4.170 | *HOXA9, FZD2, FGF8, KDM2B, HOXC11, VAX2, BMPR1A* | *-* | *-* | *-* |
| 0048598 | embryonic morphogenesis | 0.005 | 3.72 | *MESP2, HOXA9, FZD2, FGF8, KDM2B, HOXC11, VAX2, BMPR1A* | *-* | *-* | *-* |
| 0048704 | embryonic skeletal system morphogenesis | 0.044 | 8.856 | *HOXA9, FGF8, HOXC11* | *-* | *-* | *-* |
| 0045597 | positive regulation of cell differentiation | 0.044 | 2.42 | *XRCC6, BMP2, FGF8, BCL6, OTP, NRG1, NID1, BMPR1A* | *-* | *-* | *-* |
| 0045664 | regulation of neuron differentiation | 0.032 | 5.73 | *BMP2, BCL6, DIXDC1, CNTN4* | *-* | *-* | *-* |
| 0046622 | positive regulation of organ growth | 0.016 | 15.46 | *FGF8, NRG1, BMPR1A* | *-* | *-* | *-* |
| 0046661 | male sex differentiation | 0.003 | 8.01 | *HOXA9, FGF8, SYCP2, SCAPER, BMPR1A* | *-* | *-* | *-* |
| 0048232 | male gamete generation | 0.038 | 2.78 | *HOXA9, SYCP2, KDM2B, BCL6, TEX15, ACRBP, SCAPER* | *-* | *-* | *-* |
| 0003006 | developmental process involved in reproduction | 0.033 | 2.36 | *HOXA9, FGF8, SYCP2, KDM2B, BCL6, TEX15, ACRBP, SCAPER, BMPR1A* | *-* | *-* | *-* |
| 0048382 | mesendoderm development | 0.034 | 56.68 | *FGF8, BMPR1A* | *-* | *-* | *-* |
| 0048468 | cell development | 0.013 | 1.93 | *FZD2, SYCP2, NRG1, ADCY1, PTH1R, ACRBP, VAX2, SDK1, HOXA9, BMP2, OBSCN, FGF8, BCL6, S100A6, CNTN4, BMPR1A* | *-* | *-* | *-* |
| 0048513 | animal organ development | 0.001 | 2.07 | *MESP2, DNAH11, FZD2, KDM2B, SYCP2, BNIP3, DIXDC1, NRG1, OTP, PTH1R, NID1, VAX2, HOXC11, SDK1, HOXA9, BMP2, FGF8, NFIA, CPAMD8, CNTN4, SCAPER, BMPR1A* | *-* | *-* | *-* |
| 0048646 | anatomical structure formation involved in morphogenesis | 0.002 | 3.13 | *MESP2, SDK1, BMP2, OBSCN, FZD2, FGF8, KDM2B, BCL6, EMC10, ACRBP, BMPR1A* | *-* | *-* | *-* |
| 0048729 | tissue morphogenesis | 0.017 | 3.33 | *MESP2, BMP2, FZD2, FGF8, KDM2B, NRG1, BMPR1A* | *-* | *-* | *-* |
| 0048732 | gland development | 0.019 | 3.83 | *HOXA9, BMP2, FGF8, OTP, NRG1, BMPR1A* | *-* | *-* | *-* |
| 0048736 | appendage development | 0.005 | 7.27 | *HOXA9, FGF8, NFIA, HOXC11, BMPR1A* | *-* | *-* | *-* |
| 0048856 | anatomical structure development | 0.001 | 1.66 | *MESP2, SHC3, SYCP2, DIXDC1, EMC10, OTP, ADCY1, PTH1R, NID1, HOXC11, ACRBP, HOXA9, FGF8, CPAMD8, SCAPER, CFAP45, DNAH11, FZD2, KDM2B, TFAP2E, BNIP3, NRG1, VAX2, SDK1, BMP2, OBSCN, BCL6, NFIA, S100A6, SARM1, CNTN4, BMPR1A* | *-* | *-* | *-* |
| 0048869 | cellular developmental process | 0.0179 | 1.66 | *FZD2, SYCP2, BNIP3, NRG1, OTP, ADCY1, PTH1R, TEX15, ACRBP, VAX2, SDK1, HOXA9, BMP2, OBSCN, FGF8, NFIA, BCL6, ELF4, S100A6, SARM1, CNTN4, BMPR1A* | *-* | *-* | *-* |
| 0048880 | sensory system development | 0.014 | 4.15 | *SDK1, NFIA, KDM2B, CPAMD8, VAX2, SCAPER* | *-* | *-* | *-* |
| 0050789 | regulation of biological process | 0.002 | 1.27 | *MESP2, SYCP2, TRIL, DIXDC1, OTP, EMC10, PTH1R, HOXC11, LTB4R, HOXA9, FGF8, ZNF280B, CFAP45, GARIN5A, KDM2B, NRG1, TEX15, DGKZ, RAB33A, ZNF75A, OBSCN, ELF4, S100A6, ARHGEF1, LY6E, RTN2, SHC3, KCNE3, PPM1N, ADCY1, NID1, PPM1E, IGFBPL1, ST3GAL4, STOM, TMDD1, ZNF664, SRSF12, ZNF662, GABBR2, XRCC6, DNAH11, FZD2, TFAP2E, BNIP3, SYT13, DNAJC15, VAX2, CHFR, SDK1, BMP2, NFIA, BCL6, SARM1, ECHDC3, CNTN4, BMPR1A* | *-* | *-* | *-* |
| 0050793 | regulation of developmental process | 0.003 | 2.04 | *XRCC6, FZD2, KDM2B, SYCP2, BNIP3, DIXDC1, NRG1, EMC10, OTP, NID1, HOXC11, SDK1, HOXA9, BMP2, FGF8, BCL6, SARM1, CNTN4, BMPR1A* | 0.028 | 2.17 | *RNH1, FZD2, SYCP2, GNAS, SARM1, NRG1, CNTN4, PIK3R1, LOXL2, GDF7* |
| 0051093 | negative regulation of developmental process | 0.029 | 2.43 | *HOXA9, BMP2, FGF8, SYCP2, BCL6, BNIP3, DIXDC1, CNTN4, BMPR1A* | *-* | *-* | *-* |
| 0051094 | positive regulation of developmental process | 0.010 | 2.37 | *XRCC6, BMP2, FGF8, KDM2B, BCL6, BNIP3, OTP, NRG1, EMC10, NID1, HOXC11, BMPR1A* | *-* | *-* | *-* |
| 0051146 | striated muscle cell differentiation | 0.0460 | 4.93 | *BMP2, OBSCN, NRG1, BMPR1A* | *-* | *-* | *-* |
| 0055007 | cardiac muscle cell differentiation | 0.043 | 8.95 | *BMP2, NRG1, BMPR1A* | *-* | *-* | *-* |
| 0051147 | regulation of muscle cell differentiation | 0.022 | 6.59 | *BMP2, NRG1, NID1, BMPR1A* | *-* | *-* | *-* |
| 0051216 | cartilage development | 0.026 | 6.16 | *BMP2, NFIA, PTH1R, BMPR1A* | *-* | *-* | *-* |
| 0002062 | chondrocyte differentiation | 0.049 | 9.24 | *BMP2, PTH1R, BMPR1A* | *-* | *-* | *-* |
| 0051240 | positive regulation of multicellular organismal process | 0.039 | 1.93 | *XRCC6, BMP2, GARIN5A, FGF8, KDM2B, BCL6, ST3GAL4, OTP, NRG1, EMC10, HOXC11, BMPR1A* | *-* | *-* | *-* |
| 0051252 | regulation of RNA metabolic process | 0.045 | 1.51 | *MESP2, XRCC6, FZD2, TFAP2E, KDM2B, NRG1, OTP, ADCY1, VAX2, HOXC11, ZNF75A, HOXA9, BMP2, NFIA, BCL6, ELF4, ZNF280B, ZNF664, BMPR1A, SRSF12, ZNF662* | *-* | *-* | *-* |
| 0051716 | cellular response to stimulus | 0.032 | 1.36 | *MESP2, SHC3, TRIL, DIXDC1, ADCY1, PTH1R, NID1, PPM1E, LTB4R, FGF8, IGFBPL1, TMDD1, GABBR2, XRCC6, FZD2, BNIP3, NRG1, TEX15, SYT13, DNAJC15, DGKZ, VAX2, RAB33A, CHFR, BMP2, BCL6, NFIA, S100A6, SARM1, ARHGEF1, BMPR1A, LY6E* | *-* | *-* | *-* |
| 0060128 | corticotropin hormone secreting cell differentiation | 0.010 | 188.93 | *BMP2, FGF8* | *-* | *-* | *-* |
| 0060129 | thyroid-stimulating hormone-secreting cell differentiation | 0.021 | 94.46 | *BMP2, FGF8* | *-* | *-* | *-* |
| 0060287 | epithelial cilium movement involved in determination of left/right asymmetry | 0.048 | 40.48 | *DNAH11, CFAP45* | *-* | *-* | *-* |
| 0060322 | head development | 0.002 | 3.48 | *SDK1, BMP2, FGF8, KDM2B, BNIP3, DIXDC1, OTP, CNTN4, VAX2, BMPR1A* | *-* | *-* | *-* |
| 0060411 | cardiac septum morphogenesis | 0.002 | 14.72 | *DNAH11, FZD2, FGF8, BMPR1A* | *-* | *-* | *-* |
| 0003151 | outflow tract morphogenesis | 0.032 | 10.50 | *FZD2, FGF8, BMPR1A* | *-* | *-* | *-* |
| 0003279 | cardiac septum development | 0.008 | 9.77 | *DNAH11, FZD2, FGF8, BMPR1A* | *-* | *-* | *-* |
| 0060415 | muscle tissue morphogenesis | 0.002 | 15.11 | *BMP2, FZD2, NRG1, BMPR1A* | 0.007 | 23.00 | *FZD2, MYL3, NRG1* |
| 0003222 | ventricular trabecula myocardium morphogenesis | 0.048 | 40.48 | *NRG1, BMPR1A* | *-* | *-* | *-* |
| 0048644 | muscle organ morphogenesis | 0.003 | 13.82 | *BMP2, FZD2, NRG1, BMPR1A* | 0.008 | 21.04 | *FZD2, MYL3, NRG1* |
| 0055008 | cardiac muscle tissue morphogenesis | 0.020 | 13.71 | *BMP2, NRG1, BMPR1A* | *-* | *-* | *-* |
| 0061101 | neuroendocrine cell differentiation | 0.001 | 53.14 | *BMP2, FGF8, OTP* | *-* | *-* | *-* |
| 0060563 | neuroepithelial cell differentiation | 0.006 | 25.76 | *BMP2, FGF8, OTP* | *-* | *-* | *-* |
| 0061351 | neural precursor cell proliferation | 0.006 | 10.59 | *FGF8, NFIA, DIXDC1, OTP* | *-* | *-* | *-* |
| 0065007 | biological regulation | 0.002 | 1.24 | *MESP2, SYCP2, TRIL, DIXDC1, OTP, EMC10, PTH1R, HOXC11, LTB4R, HOXA9, FGF8, ZNF280B, CFAP45, GARIN5A, KDM2B, NRG1, TEX15, DGKZ, RAB33A, ZNF75A, OBSCN, ELF4, S100A6, ARHGEF1, LY6E, RTN2, SHC3, KCNE3, PPM1N, ADCY1, NID1, PPM1E, IGFBPL1, ST3GAL4, STOM, TMDD1, ZNF664, SRSF12, ZNF662, GABBR2, XRCC6, DNAH11, FZD2, TFAP2E, ATP8B2, BNIP3, SYT13, DNAJC15, VAX2, CHFR, SDK1, BMP2, NFIA, BCL6, SARM1, ECHDC3, CNTN4, BMPR1A* | *-* | *-* | *-* |
| 0071772 | response to BMP | 0.048 | 8.50 | *BMP2, NFIA, BMPR1A* | *-* | *-* | *-* |
| 0072001 | renal system development | 0.027 | 4.35 | *BMP2, FGF8, NFIA, NID1, HOXC11* | *-* | *-* | *-* |
| 0003205 | cardiac chamber development | *-* | *-* | *-* | 0.003 | 12.92 | *DNAH11, FZD2, MYL3, NRG1* |
| 0003007 | heart morphogenesis | *-* | *-* | *-* | 0.020 | 8.40 | *DNAH11, FZD2, MYL3, NRG1* |
| 0003206 | cardiac chamber morphogenesis | *-* | *-* | *-* | 0.001 | 17.30 | *DNAH11, FZD2, MYL3, NRG1* |
| 0003231 | cardiac ventricle development | *-* | *-* | *-* | 0.021 | 13.17 | *FZD2, MYL3, NRG1* |
| 0009653 | anatomical structure morphogenesis | *-* | *-* | *-* | 0.037 | 2.21 | *DNAH11, FZD2, SYCP2, MYL3, GNAS, NRG1, CNTN4, LOXL2, GDF7* |
| 0009887 | animal organ morphogenesis | *-* | *-* | *-* | 0.030 | 3.32 | *DNAH11, FZD2, SYCP2, MYL3, NRG1, GDF7* |
| 0021915 | neural tube development | *-* | *-* | *-* | 0.03 | 10.72 | *MARCKS, FZD2, GDF7* |
| 0033365 | protein localization to organelle | *-* | *-* | *-* |  |  | *DNAH11, SARM1, PIK3R1, DNAJC15, SPRN* |

FE – Fold enrichment

**Table S12.** *Enriched terms (p ≤ 0.05) retrieved from GO Cellular Components for the two undigested CNMs*

| Go Term ID | Term |  |  | CMF-ENZ |  |  | CNF -TEMPO |
| --- | --- | --- | --- | --- | --- | --- | --- |
|  |  | ***p*-value** | **FE** | **Genes** | ***p*-value** | **FE** | **genes** |
| 0005773 | vacuole | 0.036 | 3.80 | *PLAC8, GPC1, NAPRT, GPR137, ARHGAP45* | - | - | *-* |
| 0005775 | vacuolar lumen | 0.002 | 15.40 | *PLAC8, GPC1, NAPRT, ARHGAP45* | 0.033 | 10.19 | *GPC1, NAPRT, ARHGAP45* |
| 0000323 | lytic vacuole | 0.025 | 4.290 | *PLAC8, GPC1, NAPRT, GPR137, ARHGAP45* | - | - | *-* |
| 0005764 | lysosome | 0.025 | 4.290 | *PLAC8, GPC1, NAPRT, GPR137, ARHGAP45* | - | - | *-* |
| 0005766 | primary lysosome | 0.020 | 13.33 | *PLAC8, NAPRT, ARHGAP45* | - | - | *-* |
| 0005829 | cytosol | 0.035 | 1.69 | *NDRG4, BAD, PLEKHG5, FAAP20, FAM217B, CABIN1, STK3, ARHGAP45, EHD2, GPC1, NAPRT, POLR1C, BCAT1, OBSL1* | 0.035 | 9.86 | *LRRC14, CCDC106, FAM217B, CABIN1, ARHGAP45, FABP1, GPC1, NAPRT, POLR1C, S100A6, GNAS, SARM1, ARHGEF1, PDE8B, OBSL1, NINL* |
| 0031974 | membrane-enclosed lumen | 0.014 | 1.75 | *KDM2B, SYCP2, ZBTB45, GRHL3, SOX12, FAAP20, FAM217B, CABIN1, ARHGAP45, PLAC8, HOXA9, ELF4, GPC1, NAPRT, POLR1C, CHGB* | - | - | *-* |
| 0035578 | azurophil granule lumen | 0.007 | 22.86 | *PLAC8, NAPRT, ARHGAP45* | - | - | *-* |
| 0042582 | azurophil granule | 0.020 | 13.33 | *PLAC8, NAPRT, ARHGAP45* | - | - | *-* |
| 0043226 | organelle | 0.042 | 1.21 | *NDRG4, SYCP2, ZBTB45, NID1, FAAP20, STK3, CABIN1, PLAC8, HOXA9, GPC1, NAPRT, GPR137, CHGB, KDM2B, BAD, PLEKHG5, GRHL3, SOX12, FAM217B, ARHGAP45, MYO1D, EHD2, ELF4, LRFN1, POLR1C, BCAT1, OBSL1* | - | - | *-* |
| 0043227 | membrane-bounded organelle | 0.041 | 1.23 | *NDRG4, SYCP2, ZBTB45, NID1, FAAP20, STK3, CABIN1, PLAC8, HOXA9, GPC1, NAPRT, GPR137, CHGB, KDM2B, BAD, PLEKHG5, GRHL3, SOX12, FAM217B, ARHGAP45, MYO1D, EHD2, ELF4, POLR1C, BCAT1, OBSL1* | - | - | *-* |
| 0043231 | intracellular membrane-bounded organelle | 0.027 | 1.29 | *NDRG4, SYCP2, ZBTB45, FAAP20, STK3, CABIN1, PLAC8, HOXA9, GPC1, NAPRT, GPR137, CHGB, KDM2B, BAD, PLEKHG5, GRHL3, SOX12, FAM217B, ARHGAP45, MYO1D, EHD2, ELF4, POLR1C, BCAT1, OBSL1* | - | - | *-* |
| 0070013 | intracellular organelle lumen | 0.014 | 1.75 | *KDM2B, SYCP2, ZBTB45, GRHL3, SOX12, FAAP20, FAM217B, CABIN1, ARHGAP45, PLAC8, HOXA9, ELF4, GPC1, NAPRT, POLR1C, CHGB* | - | - | *-* |
| 0043233 | organelle lumen | 0.014 | 1.75 | *KDM2B, SYCP2, ZBTB45, GRHL3, SOX12, FAAP20, FAM217B, CABIN1, ARHGAP45, PLAC8, HOXA9, ELF4, GPC1, NAPRT, POLR1C, CHGB* | - | - | *-* |
| 0098590 | plasma membrane region | 0.035 | 3.12 | *MYO1D, EHD2, NDRG4, OXTR, LRFN1, ARHGAP45* | - | - | *-* |
| 0001726 | ruffle | - | - | - | 0.021 | 1.71 | *S100A6, GNAS, ARHGAP45* |

FE – Fold enrichment

**Table S13.** *Enriched terms (p ≤ 0.05) retrieved from GO Cellular Components for the two digested CNMs*

| Go Term ID | Term |  |  | DIG CMF-ENZ |  |  | DIG CNF -TEMPO |
| --- | --- | --- | --- | --- | --- | --- | --- |
|  |  | ***p*-value** | **FE** | **Genes** | ***p*-value** | **FE** | **genes** |
| 0005576 | extracellular region | 0.015 | 1.99 | *XRCC6, DNAH11, NRG1, EMC10, NID1, ACRBP, BMP2, FGF8, ADAMTSL5, CPAMD8, S100A6, ST3GAL4, CNTN4, CFAP45, LY6E* | - | - | - |
| 0005886 | plasma membrane | 0.042 | 1.39 | *RASL12, SHC3, KCNE3, ADCY1, PTH1R, NID1, LTB4R, CATIP, CPAMD8, STOM, REM2, GABBR2, FZD2, ATP8B2, COL23A1, NRG1, SYT13, DGKZ, RAB33A, SNUPN, SDK1, BMP2, OBSCN, S100A6, ARHGEF1, CNTN4, BMPR1A, LY6E* | - | - | - |
| 0097728 | 9+0 motile cilium | 0.018 | 111.40 | *DNAH11, CFAP45* | - | - | - |

FE – Fold enrichment

**Table S14.** *Enriched terms (p ≤ 0.05) retrieved from GO Molecular Functions for the two undigested CNMs*

| Go Term ID | Term | CMF-ENZ | | | CNF -TEMPO | | |
| --- | --- | --- | --- | --- | --- | --- | --- |
|  |  | ***p*-value** | **FE** | ***Genes*** | ***p*-value** | **FE** | **genes** |
| 0000978 | RNA polymerase II cis-regulatory region sequence-specific DNA binding | 0.035 | 3.12 | *HOXA9, KDM2B, ELF4, GRHL3, ZBTB45, SOX12* | - | - | - |
| 0000987 | cis-regulatory region sequence-specific DNA binding | 0.037 | 3.06 | *HOXA9, KDM2B, ELF4, GRHL3, ZBTB45, SOX12* | - | - | - |
| 0001216 | DNA-binding transcription activator activity | 0.032 | 5.49 | *HOXA9, ELF4, GRHL3, SOX12* | - | - | - |
| 0001228 | DNA-binding transcription activator activity, RNA polymerase II-specific | 0.031 | 5.55 | *HOXA9, ELF4, GRHL3, SOX12* | - | - | - |
| 0043236 | laminin binding | 0.041 | 45.58 | *GPC1, NID1* | 0.049 | 38.68 | GPC1, NID1 |
| 0005515 | protein binding | - | - | *-* | 0.007 | 1.25 | *LRRC14, RASL12, CHD7, ZBTB45, CCDC106, NID1, HS6ST1, CABIN1, HHEX, CSRP1, GPC1, NAPRT, TIMM17B, RGS20, NINL, IAH1, KDM2B, THNSL2, BRI3BP, FAM217B, ARHGAP45, FABP1, MARCKS, POLR1C, S100A6, TCEA2, GNAS, SARM1, ADAM8, ARHGEF1, OBSL1* |
| 0036094 | small molecule binding | - | - | *-* | 0.048 | 1.50 | *RASL12, KDM2B, CHD7, ZBTB45, NID1, THNSL2, ARHGAP45, FABP1, CSRP1, GPC1, NAPRT, S100A6, TCEA2, GNAS, ADAM8, PDE8B, NINL* |
| 0043167 | ion binding | - | - | *-* | 0.036 | 1.55 | *RASL12, KDM2B, CHD7, ZBTB45, NID1, THNSL2, ARHGAP45, FABP1, CSRP1, GPC1, NAPRT, S100A6, TCEA2, GNAS, ADAM8, PDE8B, NINL* |
| 0043169 | cation binding | - | - | *-* | 0.029 | 1.76 | *KDM2B, ZBTB45, NID1, THNSL2, ARHGAP45, CSRP1, GPC1, NAPRT, S100A6, TCEA2, GNAS, ADAM8, PDE8B, NINL* |
| 0046914 | transition metal ion binding |  |  |  | 0.046 | 2.92 | *KDM2B, CSRP1, GPC1, S100A6, TCEA2, ADAM8* |

FE – Fold enrichment

**Table S15.** *Enriched terms (p ≤ 0.05) retrieved from GO Molecular Functions for the two digested CNMs*

| Go Term ID | Term | DIG CMF-ENZ | | | DIG CNF -TEMPO | | |
| --- | --- | --- | --- | --- | --- | --- | --- |
|  |  | ***p*-value** | **FE** | **Genes** | ***p*-value** | **FE** | **genes** |
| 0000977 | RNA polymerase II transcription regulatory region sequence-specific DNA binding | 0.002 | 2.55 | *MESP2, TFAP2E, KDM2B, OTP, VAX2, HOXC11, ZNF75A, HOXA9, NFIA, BCL6, ELF4, ZNF280B, ZNF664, ZNF662* | - | - | - |
| 0000976 | transcription cis-regulatory region binding | 0.002 | 2.54 | *MESP2, XRCC6, TFAP2E, KDM2B, OTP, VAX2, HOXC11, ZNF75A, HOXA9, NFIA, BCL6, ELF4, ZNF280B, ZNF664, ZNF662* | - | - | - |
| 0000978 | RNA polymerase II cis-regulatory region sequence-specific DNA binding | 0.008 | 2.46 | *MESP2, HOXA9, TFAP2E, NFIA, KDM2B, BCL6, ELF4, ZNF280B, HOXC11, VAX2, ZNF664, ZNF662* | - | - | - |
| 0000987 | cis-regulatory region sequence-specific DNA binding | 0.009 | 2.41 | *MESP2, HOXA9, TFAP2E, NFIA, KDM2B, BCL6, ELF4, ZNF280B, HOXC11, VAX2, ZNF664, ZNF662* | - | - | - |
| 1990837 | sequence-specific double-stranded DNA binding | 0.002 | 2.45 | *MESP2, XRCC6, TFAP2E, KDM2B, OTP, VAX2, HOXC11, ZNF75A, HOXA9, NFIA, BCL6, ELF4, ZNF280B, ZNF664, ZNF662* | - | - | - |
| 0000981 | DNA-binding transcription factor activity, RNA polymerase II-specific | 0.007 | 2.39 | *MESP2, TFAP2E, OTP, VAX2, HOXC11, ZNF75A, HOXA9, NFIA, BCL6, ELF4, ZNF280B, ZNF664, ZNF662* | - | - | - |
| 0003700 | DNA-binding transcription factor activity | 0.009 | 2.30 | *MESP2, TFAP2E, OTP, VAX2, HOXC11, ZNF75A, HOXA9, NFIA, BCL6, ELF4, ZNF280B, ZNF664, ZNF662* | - | - | - |
| 0001067 | transcription regulatory region nucleic acid binding | 0.002 | 2.53 | *MESP2, XRCC6, TFAP2E, KDM2B, OTP, VAX2, HOXC11, ZNF75A, HOXA9, NFIA, BCL6, ELF4, ZNF280B, ZNF664, ZNF662* | - | - | - |
| 0001161 | intronic transcription regulatory region sequence-specific DNA binding | 0.040 | 49.02 | *BCL6, VAX2* | - | - | - |
| 0003690 | double-stranded DNA binding | 0.004 | 2.30 | *MESP2, XRCC6, TFAP2E, KDM2B, OTP, VAX2, HOXC11, ZNF75A, HOXA9, NFIA, BCL6, ELF4, ZNF280B, ZNF664, ZNF662* | - | - | - |
| 0043565 | sequence-specific DNA binding | 0.004 | 2.29 | *MESP2, XRCC6, TFAP2E, KDM2B, OTP, VAX2, HOXC11, ZNF75A, HOXA9, NFIA, BCL6, ELF4, ZNF280B, ZNF664, ZNF662* | - | - | - |
| 0005515 | protein binding | 0.025 | 1.14 | *MESP2, TRIL, DIXDC1, OTP, PTH1R, HOXC11, HOXA9, CATIP, FGF8, ADAMTSL5, SCAPER, NINL, CFAP45, IAH1, KDM2B, COL23A1, LMNTD2, NRG1, DGKZ, RAB33A, ZNF75A, OBSCN, ELF4, S100A6, ARHGEF1, GNE, LY6E, RTN2, RASL12, SHC3, KCNE3, ADCY1, NID1, PPM1E, IGFBPL1, STOM, ZNF664, REM2, CLHC1, SRSF12, ANKRD29, GABBR2, XRCC6, DNAH11, FZD2, TFAP2E, ATP8B2, BNIP3, SYT13, DNAJC15, VAX2, CHFR, SNUPN, FAM217B, SDK1, BMP2, NFIA, BCL6, SARM1, CNTN4, BMPR1A* | - | - | - |
| 0097159 | organic cyclic compound binding | 0.040 | 1.33 | *MESP2, RASL12, SYCP2, OTP, ADCY1, HOXC11, LTB4R, HOXA9, ZNF280B, ZNF664, SCAPER, REM2, CFAP45, SRSF12, ZNF662, XRCC6, DNAH11, KDM2B, TFAP2E, ATP8B2, DGKZ, VAX2, ZNF75A, RAB33A, SNUPN, CHFR, OBSCN, BCL6, NFIA, ELF4, ARHGEF1, BMPR1A, GNE* | - | - | - |
| 0097367 | carbohydrate derivative binding | 0.023 | 1.81 | *RASL12, XRCC6, DNAH11, ATP8B2, TRIL, COL23A1, ADCY1, NID1, DGKZ, RAB33A, OBSCN, ADAMTSL5, REM2, CFAP45, GNE, BMPR1A* | - | - | - |
| 0140110 | transcription regulator activity | 0.016 | 1.96 | *MESP2, TFAP2E, KDM2B, NRG1, OTP, VAX2, HOXC11, ZNF75A, HOXA9, NFIA, BCL6, ELF4, ZNF280B, ZNF664, ZNF662* | - | - | - |
| 0005159 | insulin-like growth factor receptor binding | - | - | - | 0.030 | 62.97 | *GNAS, PIK3R1* |
| 0043125 | ErbB-3 class receptor binding | - | - | - | 0.010 | 201.5 | *NRG1, PIK3R1* |
| 0098772 | molecular function regulator activity | - | - | - | 0.023 | 2.26 | *RASL12, RNH1, GNAS, NRG1, PIK3R1, DNAJC15, REM2, CABIN1, ARHGAP45, GDF7* |

FE – Fold enrichment
